# Extensive load of somatic CNVs in the human placenta

**DOI:** 10.1038/srep08342

**Published:** 2015-02-10

**Authors:** Laura Kasak, Kristiina Rull, Pille Vaas, Pille Teesalu, Maris Laan

**Affiliations:** 1 Human Molecular Genetics Research Group, Institute of Molecular and Cell Biology, University of Tartu, Riia St. 23, Tartu 51010, Estonia; 2 Department of Obstetrics and Gynaecology, University of Tartu, Puusepa St. 8, Tartu 51014, Estonia; 3 Women's Clinic of Tartu University Hospital, Puusepa St. 8, Tartu 51014, Estonia

## Abstract

Placenta is a temporary, but indispensable organ in mammalian pregnancy. From its basic nature, it exhibits highly invasive tumour-like properties facilitating effective implantation through trophoblast cell proliferation and migration, and a critical role in pregnancy success. We hypothesized that similarly to cancer, somatic genomic rearrangements are promoted in the support of placental function. Here we present the first profiling of copy number variations (CNVs) in human placental genomes, showing an extensive load of somatic CNVs, especially duplications and suggesting that this phenomenon may be critical for normal gestation. Placental somatic CNVs were significantly enriched in genes involved in cell adhesion, immunity, embryonic development and cell cycle. Overrepresentation of imprinted genes in somatic duplications suggests that amplified gene copies may represent an alternative mechanism to support parent-of-origin specific gene expression. Placentas from pregnancy complications exhibited significantly altered CNV profile compared to normal gestations, indicative to the clinical implications of the study.

Placenta is a unique, evolutionary young and temporary organ in mammalian pregnancy. On one hand, it mediates the transport of oxygen, nutrients and metabolic wastes across the maternal-fetal barrier. On the other hand, there is increasing evidence of placenta to act as an extra-embryonic organ with important contribution to the growth, programming and development of the fetus, as well as to the maternal gestational metabolism^1^. Rapid development of placenta among mammals has been explained by the plasticity of the placental genome modulated by two evolutionary mechanisms – co-option and adaptive evolution of ancient genes involved in growth and metabolism^2^,^3^ and emergence of novel placental genes by locus duplication events^4^,^5^.

From its basic nature, placenta is a highly invasive tumour-like structure facilitating effective implantation during a short time-span. Only human and great ape pregnancies are characterized by deep invasion of extravillous cytotrophoblasts into the uterus and spiral arteries in the placental bed, leading to their extensive remodelling and transformation into large vessels of low resistance^6^. The process of intensive trophoblast invasion resembles the aggressive behaviour of malignancies and there are striking similarities between the molecular mechanisms responsible for the proliferative, migratory and invasive properties of placental cells and those of cancer cells^7^,^8^. Tumour progression has been directly linked with the occurrence of spontaneous structural genomic rearrangements in cancerous cells leading to the high heterogeneity of somatic copy number variants (CNVs; deletions, duplications)^9^,^10^. Although there is an abundance of data on the role of somatic CNVs in cancer progression, it is unknown whether and to what extent genomic rearrangements contribute to the trophoblast invasion in pregnancy, essential for normal implantation and placental function. Studies focusing on structural genomic variants in the placental genome in human have been restricted to two reports in small patient groups addressing rare CNVs in cases of recurrent miscarriage and unexplained stillbirths^11^,^12^. However, a recent study has shown that trophoblast giant cells (TGC) of the mouse placenta contain 47 regions (totalling 6% of the genome) where genomic copies are underrepresented and it was suggested that large scale CNVs might represent a normal feature of the mammalian placental genome^13^.

We hypothesized that similarly to cancer progression, placental development and function need large and simultaneously occurring genomic rearrangements to guarantee gene expression patterns facilitating active proliferation, migration and finally deep trophoblast invasion required for human pregnancy. This study addresses for the first time the profile of CNVs in the human placental genome.

## Results

### High load of CNVs in the placental genomes

**Figure 1:**
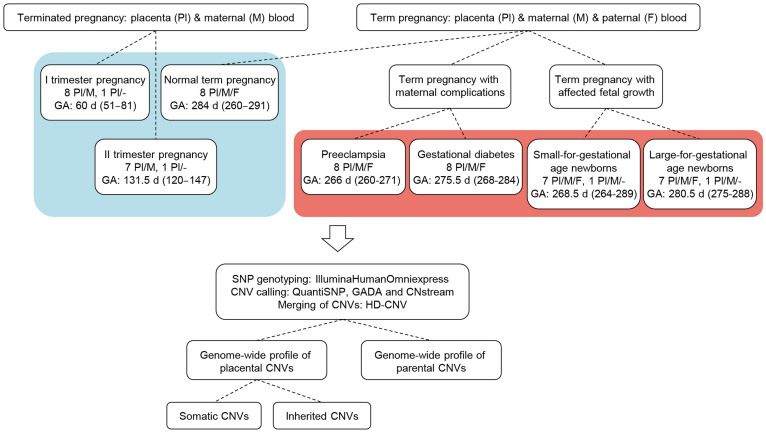
Study design. The sample set consists of three trimesters of normal pregnancy (blue background) and four groups of complicated pregnancies (red background). Genotyping and whole-genome profiling of copy number variations (CNVs) with three parallel algorithms was performed. CNVs were dissected into parental, and placental inherited and somatic structural genomic variants. Pl/M, placenta/mother duos; Pl, placenta; Pl/M/F, placenta/mother/father trios; GA, gestational age, median (range) at placental sampling within the study group is given; d, days; GADA, Genome Alteration Detection Algorithm; HD-CNV, Hotspot Detector for Copy Number Variants.

We analysed genome-wide profile of autosomal CNVs in 38 family trios comprised of placental and maternal-paternal blood DNA, 17 placenta-mother duos and two singleton placental samples (Fig. 1). The placental DNA samples represented all three trimesters of normal pregnancies (n = 9, 8 and 8, respectively), and term cases of late gestational complications (each, n = 8) of the mother (severe preeclampsia, PE; gestational diabetes, GD) or the newborn (small-for-gestational age, SGA; large-for-gestational age, LGA) (Table 1). We performed CNV calling based on genome-wide genotyping dataset (Illumina HumanOmniExpress-12-v1/24-v1 BeadChip) by applying three algorithms, QuantiSNP^14^, GADA^15^ (Genome Alteration Detection Algorithm) and CNstream^16^ in parallel. The acquired CNV calls were merged with HD-CNV^17^ (Hotspot Detector for Copy Number Variants) program and only the CNVs predicted by at least two programs were considered in subsequent analysis.

**Figure 2:**
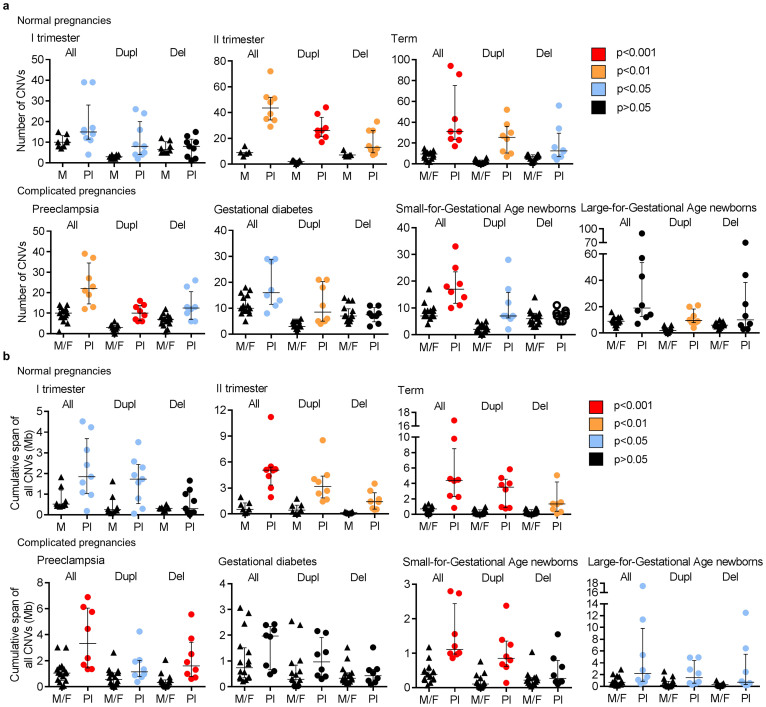
Increased load of CNVs in the genome of placenta compared to parental blood. (a) Number of CNVs detected in the 1^st^ and 2^nd^ trimester placenta-mother (Pl-M) and term placenta-mother-father (Pl-M-F) samples across normal gestation and in pregnancy complications at term. (b) Cumulative burden of CNVs (in Mb) in the placental genome exceeds total CNV load in the parental genome. P-values for the differences among the groups were calculated by Welch two-sample t-test/Wilcoxon rank sum test. Error bars show median values with interquartile range. All, duplication and deletion CNVs; Dupl, duplications; Del, deletions.

All analysed placental samples (n = 57) exhibited a significant enrichment in CNVs compared to the respective parental samples (Table 2, Fig. 2a, Supplementary Tables 1–2, Supplementary Tables 3a,b;4). We determined a threefold excess in the amount of CNVs in the placental compared to the parental blood DNA (mean no of CNVs/genome: 28.5 vs 9.5; *P* < 2.2 × 10^−16^) (Table 2). The mean placental/parental ratio for the number of identified duplications per genome (15.3/2.7) was as high as 5.7 (*P* < 2.2 × 10^−16^). Normal pregnancy exhibited a significant gradient of increasing fraction of CNVs, particularly duplications, from the first trimester towards term. Notably, the major enrichment in CNVs occurs between the 1^st^ and 2^nd^ trimesters of the pregnancy (mean number of CNVs/placental DNA: 18.6 vs 45.0, respectively; *P* = 0.006; Supplementary Fig. 1, Supplementary Table 1).

Among all identified placental CNVs (n = 1,624) the average loss/gain ratio was 0.9, which represents a strong deviation from the expectation of 2–3 fold excess of deletions compared to duplications based on previously published data on other human tissues^18^,^19^. Duplications in the placental genome were on average 0.7-fold shorter (mean 183.8 vs 130.8 kb, respectively; *P* > 0.05) and deletions 2.4-fold longer (49.8 vs 117.6 kb; *P* < 2.2 × 10^−16^) compared to the parental genome. Overall burden of deletions and duplications in the placental genome exceeded >4 times the cumulative span of CNVs in the parental genome (mean 3.6 vs 0.8 Mb; *P* = 9.1 × 10^−14^; Table 2, Fig. 2b).

Two placental DNAs were subjected to external experimental validation to confirm the extensive amount of CNVs in the placenta implemented by a commercial service provider (Atlas Biolabs GmbH; Berlin, Germany) using an alternative platform for CNV profiling (Agilent SurePrint G3 Human CGH 2 × 400 K array). Samples selected for validation had exhibited the top total load of CNVs among the placentas representing normal term pregnancies (vaginal delivery; XY karyotype) and LGA newborns (caesarean section; XY karyotype). The aCGH-based analysis confirmed a high load of genomic rearrangements in the two analysed samples and validated 56% (53/94) and 49% (28/57) of all CNVs identified by the SNP array (Supplementary Fig. 2). The detection of all existing CNVs by both approaches has its limits due to principal differences in methodology, probe distribution (715,000 vs 400,000 markers; 2.1 vs 5.3 kb spacing) and analysis (e.g. CNV calling principles; reference DNA used in aCGH etc)^20^. Many of the CNV calls detected by only one platform may be true positives and were likely missed by either of the methods due to stringent criteria (e.g. using three different algorithms for CNV calling from SNP array data).

The analysed maternal (n = 55) and paternal (n = 38) DNA samples (in total, 879 CNVs) did not differ for the mean number (9.5 and 9.3, respectively), cumulative span (both 0.8 Mb), and mean deletion/duplication ratio (2.9, 2.1) of CNVs per genome (Supplementary Table 4).

### Profiling of inherited and somatic placental CNVs

**Figure 3:**
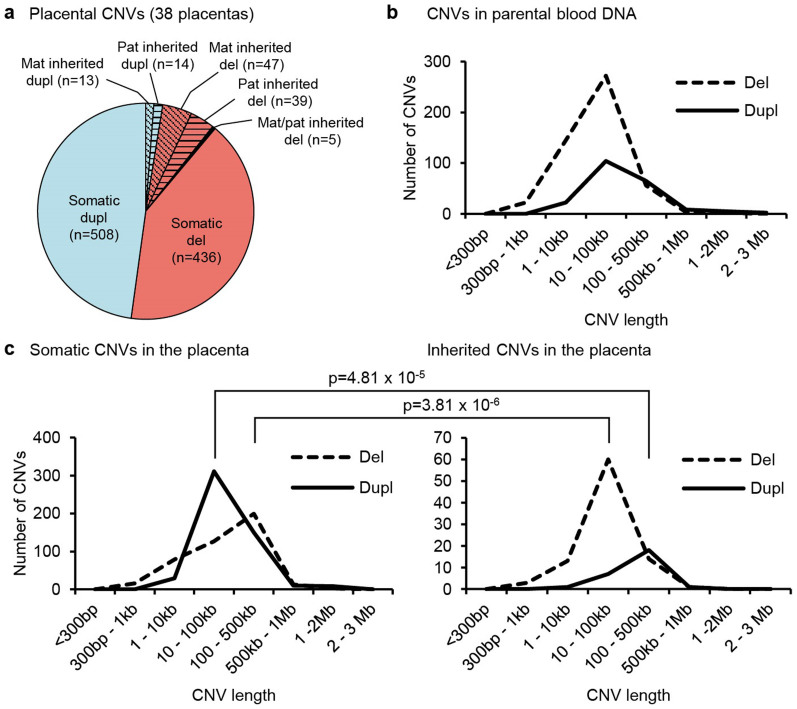
Distribution of placental and parental CNVs. (a) Distribution of somatic and inherited placental CNVs. (b) Length distribution of parental blood CNVs. (c) Length distribution of placental CNVs. P-values were calculated by Wilcoxon rank sum test. Dupl, duplications; Del, deletions; Mat, maternal; Pat, paternal.

The generated dataset for family trios (n = 38) comprising of term placental and maternal-paternal blood DNA samples enabled dissection of placental CNVs into inherited and somatic structural genomic variants. Identified inherited CNVs (n = 118, 11.1%) had been transmitted equally from the mother and the father (Fig. 3a) and their overall load, size and loss/gain profile was similar to the parental genome (Fig. 3b,c). However, the majority of the CNVs identified in the placental genome were classified as somatic, not identified in either of the parental genomes (n = 944; 88.9%; Fig. 3a). Somatic deletions were significantly longer (mean 144.6 vs 63.9 kb; *P* = 3.81 × 10^−6^) and duplications shorter (mean 125.0 vs 194.1 kb; *P* = 4.81 × 10^−5^) compared to the size distribution of inherited CNVs (Fig. 3c). This contradicts a typical CNV profile in the human genome, where large deletions are tolerated worse than duplications of long segments^21^.

We carried out functional enrichment analysis implemented by g:Profiler software^22^ for the somatic and inherited placental CNVs. Somatic placental duplications were significantly enriched in genes belonging to biological pathways relevant to fetal development and placental function, such as ‘anterior/posterior pattern specification’ (GO:0009952; 10.4% of genes rearranged in the pathway; *P* = 1.34 × 10^−5^, corrected for multiple testing), ‘homophilic cell adhesion’ (GO:0007156; 12.2% of genes; *P* = 3.48 × 10^−5^), and ‘blood microparticle processes’ (GO:0072562; 11.3% of genes; *P* = 7.35 × 10^−4^) (Table 3, Supplementary Table 5a). In addition, we found enrichment for genes targeted by microRNA mir-210 (34 genes; *P* = 3.9 × 10^−3^), linked to trophoblast migration and invasion process and significantly upregulated in preeclamptic placentas^23^.

Analysis of genes under placental somatic deletions identified ‘cell cycle process’ (GO:0022402; 5.2% of genes; *P* = 3.49 × 10^−5^) and ‘purine ribonucleoside triphosphate binding’ (GO:0022402; 4.5% of genes; *P* = 4.6 × 10^−5^) as top affected biological functions, and ‘microtubule-based process’ (GO:0007017; 6.4% of genes; *P* = 1.87 × 10^−3^) and ‘mitosis’ (GO:0007067; 7.1% of genes; *P* = 3.04 × 10^−3^) as more specific pathways. Consistently, the absence of mitosis in the placental syncytiotrophoblast resulting in multinucleated cells was reported already in 1962^24^.

Functional profiling of placental CNVs by g:Profiler software was validated by WebGestalt^25^ analysis. The alternative approach resulted in concordant output highlighting ‘anterior/posterior patter specification’ (GO:0009952; *P* = 2.32 × 10^−6^) and ‘homophilic cell adhesion’ (GO:0007156; *P* = 1.13 × 10^−5^) as top biological pathways for the genes involved in somatic duplications and cell cycle related pathways for the genes under somatic deletions (Supplementary Table 6).

The inherited placental CNVs as well as parental blood CNVs represented mostly benign, polymorphic structural variants of multi-copy loci belonging to e.g. biological pathways ‘amylase activity’, ‘olfactory receptor activity’ (Table 3, Supplementary Table 5b,e) or the *Pregnancy-specific Glycoprotein* (*PSG*) gene cluster^26^ (GO:0007565 ‘female pregnancy’).

### Genomic distribution of somatic placental CNVs

Although chromosomal mapping of placental somatic CNVs revealed their distribution across the entire genome, some genomic hotpots were clearly identifiable (Supplementary Fig. 3). We found 91 CNV regions that were rearranged in at least three term placentas (Supplementary Table 7). Almost two-thirds of these recurrent somatic rearrangements were duplications and 75 regions included genes. The largest recurrent CNV region represented 117 duplicated loci from the *immunoglobulin heavy-chain* (*IGH*) gene cluster at 14q32.33 (623 kb; 6/38 placentas; including one validation sample, confirmed by aCGH). The *IGH* locus controls antibody heavy-chain biosynthesis, which is essential for the adaptive immune response. The copy number differences of IGH variable genes (*IGHV*) in regulatory sequences seem to be a principle component of individual differences in *IGHV* gene usage in expressed antibody repertoires^27^. Most frequent somatic duplications involved the *EPHA7* (6q16.1; 152 kb CNV; 12 placentas), the *C2CD5* (12p12.1; 29 kb CNV; n = 11) and the *CSMD1* (8p23.2; 19.4 and 5.7 kb duplications, n = 11 and 3, respectively) genes. The most prevalent somatic deletions involved a pericentromeric region at 11p11.2-p11.12 (182.4 kb, uncharacterized RNA-coding genes; 21/38 placentas) and a region comprised of *VPRBP* and *RAD54L2* genes (3p21.2; 51 kb; n = 11; confirmed by aCGH for both validation samples), involved in cell proliferation through chromatin remodelling^28^,^29^. Interestingly, five of the recurrent somatic CNV regions identified in the human placenta overlapped with respective syntenic regions in the mouse genome described with decreased copy number in murine placental polyploid TGC, involving e.g. the genes *EPHA7*, *CSMD3*, *COL11A1*, *DPYD* and *GRIK2*^13^ (Supplementary Table 8).

All recurrent rearrangements coincided with known somatic CNVs in various cancers (http://cancer.sanger.ac.uk/cosmic/conan/search). Genome structural rearrangements represent a critical part in cancer progression and the data support their similar role in placental biology.

### Enrichment of imprinted genes in somatic duplications

The placenta is notable for its high and prolific expression of imprinted genes^30^. Improper expression of imprinted genes may lead to abnormalities in placental function and embryonic development^31^. We screened the Geneimprint database (208 imprinted human genes; http://www.geneimprint.com/) and a recently published list of novel imprinted loci^32^ for the genes disrupted by the identified somatic placental CNVs. We identified a highly significant enrichment of imprinted genes involved in the placental somatic duplications (21/1,180 duplicated genes vs 214/57,952 Ensembl genes; *P* = 2.67 × 10^−13^) (Table 4). Majority of these genes are maternally expressed and important in placental and embryonic development. For example *CTNNA3* (duplicated in 5 placentas) promotes the invasiveness of extravillous trophoblasts as it is essential for the formation of cell–cell adhesion complexes^33^.

Also some of the somatic deletions in the placental genome involved imprinted genes: paternally expressed *FAM59A* (deleted in 1 placenta) and *SNRPN* (2 placentas), maternally expressed *NTM* (1 placenta).

### Altered profile of somatic duplications in pregnancy complications

The highest number of somatic duplications and the largest cumulative span of all somatic CNVs were identified in the placental genomes from normal term pregnancies (Supplementary Table 1). The placentas representing maternal or fetal pregnancy complications were characterized by significantly lower number of somatic duplications compared to normal term pregnancy (mean 11.0 vs 25.0, respectively; *P* = 7.83 × 10^−3^; Fig. 2, 4a). The lowest total number and cumulative span of CNVs was detected in the SGA group (mean CNVs/sample: 18.3 vs 43.6 in normal term). Normal and complicated term pregnancies did not differ for the number of somatic deletions. No statistical difference in the distribution of CNVs in the parental genomes representing the cases of normal and complicated pregnancies was detected (Supplementary Table 2).

**Figure 4:**
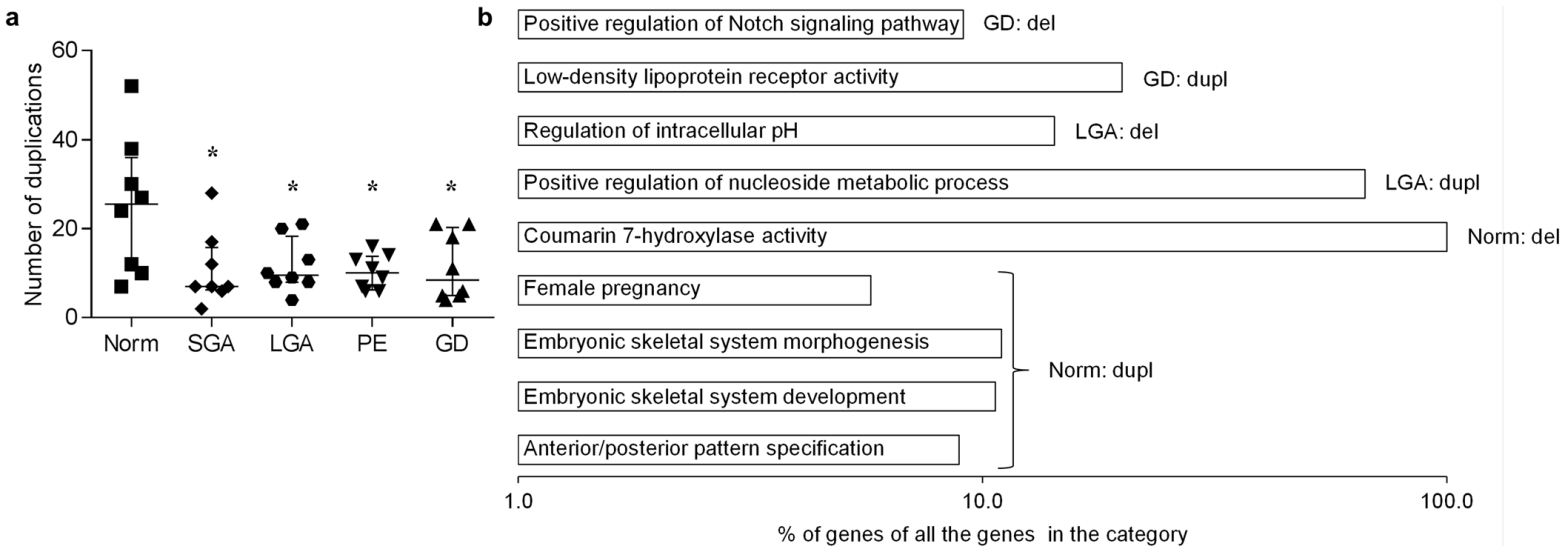
Differential profile of somatic CNVs in normal and complicated pregnancies. (a) Load of duplications in placental genomes from normal term compared to complicated pregnancies; *p < 0.05 (Welch two-sample t-test/Wilcoxon rank sum test). (b) Functional categories exhibiting significant (FDR < 0.05) enrichment among the genes underlying the placental somatic CNVs and involving >5% of genes in the pathway (shown in log scale). Norm, normal term; GD, gestational diabetes; PE, preeclampsia; SGA and LGA, small- and large-for-gestational age newborns; Dupl, duplication; Del, deletion.

We stratified all somatic placental CNVs into specific and shared variants for the five term pregnancy groups (n = 8 in each group). For the genomic rearrangements detected in only one patient group, further functional enrichment analysis was performed to identify biological pathways enriched in each pregnancy curriculum. Placentas from normal, uncomplicated pregnancies confirmed highly significant enrichment of duplicated genes in the biological pathways involved in normal fetal development (Fig. 4b, Supplementary Table 5c). Notably, somatic duplications specific to the placentas from gestational diabetes (GD) cases clustered in the functional category ‘low-density lipoprotein receptor activity’ (GO:0005041; *P* = 2.87 × 10^−2^; Supplementary Table 5d). One of the involved genes, *CD36*, is highly relevant in the context of GD. Placenta exhibits one of the highest *CD36* gene expression levels (The Human Protein Atlas; http://www.proteinatlas.org/) and circulating CD36 has been identified as a biomarker for type 2 diabetes^34^. Notably, majority of the genes under this molecular function category have been previously linked to diabetes (7/10 genes) and/or placental function (7/10) (Supplementary Table 9).

## Discussion

We report an extensive load of somatic CNVs, especially duplications, in the human placental genomes across gestation and suggest that this phenomenon may be important for placental development and function to guarantee the normal progression and maintenance of pregnancy. In our study, the investigated whole-placental material did not allow equivocal determination, whether the existence of somatic CNVs is a common feature to the entire organ or it is specific to only certain placental cell types. However, as the analysed first-trimester samples represented purified chorionic villi containing cyto- and syncytiotrophoblasts, the data confirms the presence of somatic genomic rearrangements in these, solely placenta-specific cell types. Supportingly, human trophoblasts have been shown to undergo endoreduplication leading to the amplification of specific chromosomal regions and consequently, their enhanced gene expression^35^. This process enables rapid cell growth and differentiation without disturbing the cytoskeleton or cell adhesion by mitosis^24^,^36^. Amplification of genes involved in cellular adhesion (e.g. *PCDHA*) and regulation of immune function (e.g. *C4A-C4B, IGH* clusters) may contribute to the processes of trophoblast invasion to maternal decidua^37^ and modulation of the maternal immune tolerance in pregnancy. Duplication of genes involved in the embryonic development (e.g. *HOXA*, *HOXC*^38^) may enhance the role of placenta as an extra-embryonic organ contributing to the fetal programming^39^.

Consistent to our findings, Sher *et al*. has reported that the genomes of mouse TGCs are uniformly duplicated and that multiple genes required for mitosis and cytokinesis are transcriptionally repressed^40^. Hannibal *et al*. showed that certain regions of the TGC genome are under-replicated (UR), whereas some genes, including those linked to cell adhesion, were enriched^13^. Importantly, several of the murine genes in the UR domains coincide with the genes disrupted by the human placental somatic CNVs in this study, e.g. *EPHA7*, *EPHA5*, *CDH19* and *PTPRD* (Supplementary Table 8). The phenomenon may have evolved as a universal mechanism in the mammalian placenta guaranteeing rapid implantation and maximized execution of its function within the limited timeframe of pregnancy.

Excessive amount of imprinted genes disrupted by placental somatic CNVs discovered in current work confirms the importance of DNA methylation in the regulation of placental and embryonic development. We suggest that evolution towards parent-of-origin gene expression in the placenta may have included evolution of two complementary strategies – through silencing of one parental copy but also via creating additional gene copies through somatic duplication on the other parental chromosome. For several papers, the imprinting status has been suggested mainly based on studies on allelic expression in the placenta^41^,^42^. However, this approach is unable to distinguish whether the detection failure of transcript from one parental allele is due to silencing by imprinting or due to proportionally much lower expression compared to the in consort expression of duplicated gene copies on the other parental chromosome. Deletions of otherwise imprinted genes may represent alternative mechanism to guarantee the silencing of the respective parental allele.

Our findings have also important clinical implications. Firstly, we suggest that sporadic gestational complications may arise during the burst of somatic genome rearrangements in early placental development, involving genes critical in gestational metabolism. Identification of genes underlying placental somatic CNVs in pregnancy complications may provide novel biomarkers and therapeutic targets. The current study identified duplication of *CD36* encoding sCD36 detectable in maternal circulation. As sCD36 has been identified as a marker for insulin resistance^34^, it may also represent an excellent novel candidate biomarker for altered gestational metabolism. Overall, our findings highlight a novel metabolic pathway ‘low-density lipoprotein receptor activity’ potentially implicated in the development of GD. Among the members of this pathway, increased placental expression of *CD36*^43^ has been shown in gestational obesity, elevated placental OLR1 (Oxidized low-density lipoprotein receptor 1) and LDLR (LDL receptor) levels were reported in GD pregnancies^44^,^45^ and SNPs in the *LRP8* gene (encoding ApoE receptor) have been associated with fetal growth^46^. Whether the placental somatic duplications involving this pathway may promote sporadic cases of altered gestational metabolism deserves further studies.

Secondly, whereas the similarities in DNA methylation patterns among the placenta and cancerous tissues are well acknowledged^8^, this is the first report showing that the two genomes may also share common mechanisms to promote selective somatic rearrangements. Although cancer and trophoblastic cells use similar mechanisms to attain their proliferative, migratory and invasive properties^7^, placenta has developed the ability to limit its invasion as the pregnancy reaches term. Therefore placental research can make an important contribution not only to pathologic pregnancies, but also to cancer therapy development.

Additional clinical implication of our findings relates to the increasing interest in non-invasive prenatal testing based on the analysis of cell-free DNA (cfDNA) shed into the maternal circulation by the placenta. The confined placental mosaicism for aneuploidies is a commonly acknowledged phenomenon in the cytogenetic analysis of the first trimester chorionic villus samples (prevalence of 1–2%^47^). Whereas cfDNA-based prenatal screening using next generation sequencing was shown highly reliable to detect common aneuploidies^48^, extensive somatic genomic rearrangements and mosaicism in the placenta may interfere with the reliable detection of fetal CNV profile, and increase the rate of false-positive predictions.

## Methods

### Ethics statements

The study was approved by the Ethics Review Committee of Human Research of the University of Tartu, Estonia (permissions no 117/9, 16.06.2003; 146/18, 27.02.2006; 150/33, 18.06.2006; 158/80, 26.03.2007; 180/M-15, 23.03.2009) and it was carried out in compliance with the Helsinki Declaration. A written informed consent to participate in the study was obtained from each individual prior to recruitment. All study participants were recruited and the study material was collected at the Women's Clinic of Tartu University Hospital, Estonia in 2003–2011. All participants were of white European ancestry and living in Estonia. All methods were carried out in accordance with approved guidelines.

### Study groups representing the first and the second trimesters of pregnancy

Placental and maternal blood samples were obtained from females who underwent (a) elective (surgical) termination of pregnancy during first trimester (9 cases); (b) therapeutic medically induced abortion during second trimester due to maternal medical risks of pregnancy, where no fetal anomalies were detected (8 cases). Details are provided in Fig. 1 and Table 1. Normal male or female karyotype was confirmed in maternal and placental samples by routine cytogenetic analysis (United Laboratories, Tartu University Hospital).

### Study material representing normal and complicated term pregnancies

Term pregnancy cases were selected from the REPROgrammed fetal and/or maternal METAbolism (REPROMETA) study^39^,^49^,^50^,^51^. Participants (family trios/duos) of the REPROMETA study have been recruited at the delivery. The collected study material includes clinical and epidemiological data and biological samples from normal and complicated singleton pregnancies at term (gestational weeks 37–42). The biological sampling included placenta, maternal and paternal blood samples, and umbilical cord blood serum. Information about mother's diseases, smoking, somatometric data, and childbirth history was obtained from medical records during the course of pregnancy and after birth. Fetal outcome data from delivery included weeks of gestation, birth weight, birth length, head and abdominal circumferences, and placental weight. Cases with documented fetal anomalies, chromosomal abnormalities, families with history of inherited diseases and patients with known pre-existing diabetes mellitus, chronic hypertension and chronic renal disease were excluded.

The REPROMETA participants represent clinical subgroups based on the birth weight of a newborn and the absence/presence of maternal pregnancy-specific complications. The control group comprises of uncomplicated pregnancies resulting in the birth of newborn with the weight appropriate for-gestational age (normal term, birth-weight between 10–90 percentiles). Study groups of disturbed fetal growth comprise of newborns born as (i) small- (SGA, birth weight <10^th^ percentile) and (ii) large-for-gestational age (LGA, birth weight >90^th^ percentile). The weight percentiles for defining SGA and LGA were calculated on the basis of data from Estonian Medical Birth Registry^52^. Study groups of maternal pregnancy complications include maternal (i) preeclampsia (PE) and (ii) gestational diabetes (GD). All PE cases represented the severe form of late-onset preeclamptic pregnancies and were diagnosed in the presence of hypertension (systolic blood pressure ≥160 mmHg and/or diastolic blood pressure ≥110 mmHg) and/or proteinuria of ≥5 g in 24 hours. GD was diagnosed when 75 g oral glucose tolerance test performed at 24–28 weeks of gestation revealed either a fasting venous plasma glucose level of >5.1 mmol/l, and/or at 1 h and 2 h plasma glucose level of >10 mmol/l and >8.5 mmol/l glucose, respectively.

In the current study, each subgroup (normal term, PE, GD, SGA, LGA pregnancies) comprised of 8 representative families. Detailed characteristics of REPROMETA trio (n = 38) and duo (n = 2) samples included into the study are given in Fig. 1 and Table 1.

### Placental sampling

First trimester samples were obtained immediately after elective (surgical) termination of pregnancy. The samples were washed with solution containing 15 ml Dulbecco's Phosphate Buffered Saline (PBS), 0.3 ml penicillin-streptomycin solution 10000 U/10000 μg/ml and 2 drops of heparine, 5000 U/ml. The maternal cells were removed under a stereomicroscope (Discovery V8, Zeiss) and chorionic villi containing both cyto- and syncytiotrophoblast cells of fetal origin were placed into dry tube and stored at −80°C without any further manipulation (cell sorting, culturing).

The full-thickness block of 2 cm from mid- pregnancies (17–21 gestational weeks) and term pregnancies (37–42 gestational weeks) were taken from a middle region of placenta (kept at +4°C) within 1 h after medically induced abortion, caesarean section or vaginal delivery. Collected tissue samples were washed with 1× PBS to remove contamination of maternal blood and placed immediately into dry cryovial and stored at −80°C for subsequent DNA extraction. All samples were collected by the same medical personnel. In all biopsy/autopsy samples histological examination was carried out to confirm the non-malignancy of the tissues. Karyotyping of the first trimester samples confirmed normal male or female karyotype in all cases.

### Genome-wide SNP genotyping and CNV detection

Placental and blood genomic DNA was genotyped using Illumina HumanOmniExpress-12-v1/24-v1 BeadChips (>715,000 markers with median spacing 2.1 kb) at the institutional genotyping core facility (Estonian Genome Center; http://www.geenivaramu.ee/en). Samples were genotyped with an average overall call rate of 99.6% (median 99.7%). For each sample, calling of CNVs from the resulting genome-wide genotyping data was performed in parallel with three algorithms. Normalized signal intensity data was obtained through Illumina GenomeStudio software. Information on Log2 R ratios, B allele frequencies, markers and chromosomal coordinates from each sample were used for CNV identification with Hidden Markov Model-based algorithm QuantiSNP 2.3^14^ and a sparse Bayesian learning approach GADA (Genome Alteration Detection Analysis)^15^. QuantiSNP was run with default settings, the adjustment for ‘genomic waves’ in signal intensities^53^ was turned on with the ‘--gcdir’ key in addition to the default calling parameters. GADA was run using the options “–T 5 –M 3”. CNstream^16^ algorithm was used in parallel to estimate CNVs from X and Y channel intensities loaded from Illumina GenomeStudio. CNVs were calculated under the following parameters: number of probes per segment ( = 3), minimum number of probes in one segment that must exceed the threshold for identifying an amplification/deletion ( = 3), CNV frequency threshold ( = 0). CNVs with QuantiSNP log Bayes Factor value <5 and/or rearrangements shorter than 350 bp were excluded from the resulting list of CNVs. In this project we analysed only autosomal CNVs.

HD-CNV^17^ (Hotspot Detector for Copy Number Variants) was used to merge CNV regions called by alternative computational algorithms, QuantiSNP, GADA and CNstream. HD-CNV requires CNV calls as an input to detect overlapping regions among calls. A criterion of 40% reciprocal overlap between parallel CNV calls was used to define two calls as identifying the same event. All CNVs called by at least two algorithms for the same individual in the same genomic loci were considered in the subsequent global analysis. As demonstrated previously, the advantage of using the CNV predictions by more than one algorithm effectively minimizes the number of false positive calls^54^,^55^,^56^. QuantiSNP program has shown high accuracy in copy number estimation in our previous study^19^. The advantage of GADA is the speed of data processing as it is able to analyse data within a few minutes and furthermore its application within R^54^. CNstream is also fully implemented in R and is specifically designed for Illumina microarrays^16^.

### Array comparative genomic hybridization

Two placental DNAs were subjected to experimental validation of the CNV profile identified by the SNP array. Validation experiments were performed blindly by Atlas Biolabs GmbH (Berlin, Germany) using an alternative technology platform for CNV detection, Agilent SurePrint G3 Human CGH 2 × 400 K array. The validation samples were selected based on the highest CNV load within a subgroup and represented male placentas from vaginal delivery (1 normal term pregnancy) and caesarean section (1 pregnancy with an LGA newborn). The reference genomic DNA for aCGH represented an anonymous DNA sample available by the service provider. Array hybridizations, quality control and CNV calling from aCGH dataset followed the established pipeline by the commercial service provider. Briefly, the CNV profile was generated with Genomic Workbench version 7.0 (Agilent). Minimum average absolute Log Ratio for duplication ≥0.25; minimum average absolute Log Ratio for deletion ≥0.25 and minimum size of region for duplication/deletion ≥0.0 were applied. CNVs were called for the segments with at least 3 consecutive probes.

### Functional enrichment analysis

In order to acquire the up-to-date genomic annotation data for functional enrichment analysis, the latest version of the human reference sequence breakpoint coordinates were used (GRCh37/hg19). Functional enrichment analysis of CNVs was carried out separately for placental somatic and inherited CNVs and parental CNVs using g:Profiler gGOSt web-based software (http://biit.cs.ut.ee/gprofiler/)^22^,^57^. Considering all genes in user-provided gene lists or chromosomal regions, g:Profiler performs statistical gene set enrichment analysis to find which functional groups and/or biological pathways are significantly overrepresented among user-provided genes, often helping with biological interpretation of high-throughput experiments. Results of Gene Ontology (GO) and miRBase microRNA (MI) datasets with moderate hierarchical filtering were taken into account. The list of genes within the identified CNV regions was acquired using g:Profiler gConvert tool which uses information in Ensembl databases to handle hundreds of types of IDs for genes, proteins, transcripts, microarray probesets, etc, for many species, experimental platforms and biological databases^22^,^57^. Additional functional enrichment analysis of subsequent gene sets was carried out separately for somatic and inherited CNVs using WebGestalt (WEB-based GEne SeT AnaLysis Toolkit)^25^,^58^ (Supplementary Table 6). Results of Gene Ontology (GO) Analysis were taken into account. Enrichment for functional terms was considered significant for both software if the multiple testing corrected enrichment (FDR) *P*-value was <0.05.

### Statistical analysis

All statistical analyses were performed using R Statistical Software version 2.15.2 (http://www.r-project.org/). Data was tested for normality with Shapiro-Wilk normality test. By reference to the normality test results Welch Two Sample t-test or non-parametric Wilcoxon rank sum test was used. Pearson's Chi-squared test with Yates' continuity correction was applied to test the significance of number of imprinted genes among placental somatic duplication CNVs. Results with P-values <0.05 were considered significant.

## Additional information

**Accession codes**: The CNVs detected in the blood DNA have been submitted to the Database of Genomic Variants archive (DGVa; http://www.ebi.ac.uk/dgva/), accession estd216.

## Supplementary Material

Supplementary Information Supplementary Material

## Figures and Tables

**Table 1:** Parental and offspring characteristics of study samples. Data are given as means ± SD. I trimester samples consist of elective abortions; II trimester samples are medically induced abortions; normal term, SGA (small-for-gestational age), LGA (large-for-gestational age), PE (preeclampsia) and GD (gestational diabetes) samples represent term pregnancies. Parity, number of deliveries before current pregnancy; n.a, not available/not applicable. f, female; m, male

		I trimester	II trimester	Normal term	SGA	LGA	PE	GD
Mother	Age (years)	26.2 ± 5.91	27.1 ± 8.95	29.3 ± 7.85	24.5 ± 3.51	30.9 ± 5.06	27.4 ± 7.39	30.3 ± 5.15
	BMI before pregnancy (kg/m^2^)	22.9 ± 3.47	21.5 ± 2.67	23.9 ± 3.77	20.6 ± 2.92	24.83 ± 4.61	26.3 ± 5.00	26.3 ± 7.85
	Gestational weight gain (kg)	n.a	n.a	17.2 ± 3.52	13.3 ± 3.47	21.7 ± 7.25	10.9 ± 3.36	14.8 ± 5.76
	Parity (0/1/≧2)	2/5/2	5/1/2	3/4/1	7/1/0	2/2/4	6/1/1	3/3/2
	Gestational age at the placental sampling (days)	63.2 ± 11.14	131.6 ± 9.30	278.6 ± 11.54	272 ± 9.46	281.6 ± 4.41	266.1 ± 3.94	275.5 ± 5.98
	Caesarean section/vaginal	n.a	n.a	3/5	2/6	5/3	6/2	5/3
Father	Age (years)	n.a	n.a	31.8 ± 5.99	27.14 ± 5.64	35.8 ± 8.28	32.8 ± 8.94	33.5 ± 7.05
	BMI (kg/m^2^)	n.a	n.a	25.1 ± 3.83	22.7 ± 2.04	29.9 ± 5.65	28.8 ± 6.34	27.8 ± 4.34
Offspring	Birth weight (g)	n.a	n.a	3,703 ± 392	2,442 ± 235	4,726 ± 208	2,794 ± 488	4,269 ± 238
	Birth lenght (cm)	n.a	n.a	51.3 ± 1.89	46.3 ± 1.04	53.4 ± 1.18	47.6 ± 1.51	52.4 ± 1.30
	Placental weight (g)	n.a	n.a	571.3 ± 115.8	397.5 ± 89.9	816.2 ± 116.0	476.9 ± 119.9	658.1 ± 185.2
	Gender (f/m)	5/4	4/4	3/5	5/3	4/4	4/4	5/3

**Table 2:** Comparative profile of parental and placental CNVs. Data are given as means (medians), except when indicated otherwise. All, pooled duplication and deletion CNVs; n.a, not applicable; n.s, non-significant

		Parental blood DNA (n = 93)	Placental DNA (n = 57)	Placental/Parental genome ratio	Wilcoxon rank sum test *P*-value
Number of CNVs per sample	All	9.5 (9.0)	28.5 (23.0)	3.0 (2.6)	<2.2 × 10^−16^
	Duplication	2.7 (3.0)	15.3 (12.0)	5.7 (4.0)	<2.2 × 10^−16^
	Deletion	6.8 (7.0)	13.2 (9.0)	1.9 (1.3)	5.6 × 10^−5^
Cumulative span of all CNVs per sample, Mb	All	0.8 (0.6)	3.6 (2.4)	4.5 (4.0)	9.1 × 10^−14^
	Duplication	0.5 (0.3)	2.0 (1.4)	4.0 (4.7)	7.6 × 10^−13^
	Deletion	0.3 (0.3)	1.5 (0.7)	5.0 (2.3)	1.1 × 10^−7^
CNV length across samples, kb	All	87.8 (23.0)	124.7 (70.4)	1.4 (3.1)	<2.2 × 10^−16^
	Duplication	183.8 (68.3)	130.8 (74.9)	0.7 (1.1)	n.s
	Deletion	49.8 (16.5)	117.6 (63.8)	2.4 (3.9)	<2.2 × 10^−16^
CNV size range, kb	All	0.3; 2392.4	0.3; 1814.4	n.a	n.a
	Duplication	2.2; 2392.4	2.2; 1814.4	n.a	n.a
	Deletion	0.3; 1629.9	0.3; 1480.7	n.a	n.a
Ratio (loss/gain)		2.5 (2.3)	0.9 (0.8)	0.4 (0.3)	2.9 × 10^−14^

**Table 3:** Pathway analysis of placental CNVs with g:Profiler^22^. GO, Gene Ontology functional categories; MI, miRBase

Significantly overrepresented functional categories			Enrichment analysis	
Type	ID	Name	Pathway genes (%)	FDR *P*-value
*Somatic duplication CNVs*				
GO	0009952	anterior/posterior pattern specification	10.4	1.34 × 10^−5^
	0007156	homophilic cell adhesion	12.2	3.48 × 10^−5^
	0048706	embryonic skeletal system development	12.3	2.33 × 10^−4^
	0005509	calcium ion binding	5.7	2.43 × 10^−4^
	0072562	blood microparticle	11.3	7.35 × 10^−4^
	0048704	embryonic skeletal system morphogenesis	13.2	2.13 × 10^−3^
	0006956	complement activation	14.1	3.00 × 10^−2^
MI	hsa-miR-210	hsa-miR-210 regulated genes	5.2	3.90 × 10^−3^
*Somatic deletion CNVs*				
GO	0022402	cell cycle process	5.2	3.49 × 10^−5^
	0035639	purine ribonucleoside triphosphate binding	4.5	4.60 × 10^−5^
	1902589	single-organism organelle organization	4.5	1.15 × 10^−4^
	0032550	purine ribonucleoside binding	4.4	2.33 × 10^−4^
	0007017	microtubule-based process	6.4	1.87 × 10^−3^
	0043231	intracellular membrane-bounded organelle	2.9	2.14 × 10^−3^
	0007067	mitosis	7.1	3.04 × 10^−3^
	0005829	cytosol	3.8	4.51 × 10^−3^
	0005874	microtubule	6.6	2.79 × 10^−2^
*Inherited duplication CNVs*				
	no functional categories significantly enriched			
*Inherited deletion CNVs*				
GO	0007565	female pregnancy	5.2	1.54 × 10^−6^
	0016160	amylase activity	50	8.06 × 10^−4^
	0008389	coumarin 7-hydroxylase activity	100	1.61 × 10^−2^
	0004970	ionotropic glutamate receptor activity	16.7	3.22 × 10^−2^
	0005576	extracellular region	0.6	3.27 × 10^−2^
	0005234	extracellular-glutamate-gated ion channel activity	15.8	3.81 × 10^−2^
	0046226	coumarin catabolic process	66.7	4.82 × 10^−2^
MI	hsa-miR-19a	hsa-miR-19a regulated genes	1.2	1.29 × 10^−2^

**Table 4:** Imprinted genes disrupted by placental CNVs. Data modified from http://www.geneimprint.com/; * from Court *et al*. 2014. SGA, small-for-gestational age; GD, gestational diabetes; norm, normal term; PE, preeclampsia; LGA, large-for-gestational age

Gene	Location	Status	Expressed Allele	No of carriers	Groups
*Genes disrupted by somatic duplication CNVs*					
*ALDH1L1*	3q21.3 *AS*	Predicted	Maternal	3	SGA, GD, norm
*CTNNA3*	10q22.2 *AS*	Provisional Data	Maternal	5	2 GD, 1 PE, SGA, norm
*CYP1B1*	2p21 *AS*	Predicted	Paternal	3	2 PE, 1 GD
*DVL1*	1p36 *AS*	Predicted	Maternal	1	LGA
*FGFRL1*	4p16	Predicted	Maternal	1	norm
*HOXA11*	7p15-p14 *AS*	Predicted	Maternal	1	norm
*HOXA2*	7p15-p14 *AS*	Predicted	Maternal	1	norm
*HOXA3*	7p15-p14 *AS*	Predicted	Maternal	1	norm
*HOXA4*	7p15-p14 *AS*	Predicted	Maternal	1	norm
*HOXA5*	7p15-p14 *AS*	Predicted	Maternal	1	norm
*HOXC4*	12q13.3	Predicted	Maternal	1	norm
*HOXC9*	12q13.3	Predicted	Maternal	1	norm
*KCNQ1*	11p15.5	Imprinted	Maternal	1	norm
*KCNQ1OT1*	11p15	Imprinted	Paternal	1	norm
*NTM*	11q25	Imprinted	Maternal	1	SGA
*PEX10*	1p36.32 *AS*	Predicted	Maternal	2	LGA
*PKP3*	11p15	Predicted	Maternal	1	norm
*PRDM16*	1p36.23-p33	Predicted	Paternal	2	LGA
*SLC22A3*	6q26-q27	Imprinted	Maternal	6	2 PE, norm, 1 LGA, GD
*TP73*	1p36.3	Imprinted	Maternal	2	1 LGA, norm
*WDR27*	6q27	Provisional Data*	Paternal	1	SGA
*Genes disrupted by somatic deletion CNVs*					
*FAM59A*	18q12.1 *AS*	Predicted	Paternal	3	LGA, PE, norm
*NTM*	11q25	Imprinted	Maternal	1	PE
*SNRPN*	15q11.2	Imprinted	Paternal	2	norm
*Genes disrupted by inherited duplication CNVs*					
*NKX6-2*	10q26 *AS*	Predicted	Maternal	1	norm
*Genes disrupted by inherited deletion CNVs*					
*CTNNA3*	10q22.2 *AS*	Provisional Data	Maternal	1	LGA
*DGCR6*	22q11.21	Imprinted	Random	1	norm
