# Supplementary material for: Extensive load of somatic CNVs in the human placenta
Source: Sci Rep. 2015 Feb 10;5:8342. doi: 10.1038/srep08342 (PMC4914949; doi:10.1038/srep08342)
Supplement: Supplementary Information — Supplementary Material [file srep08342-s1.pdf]

Supplementary Material

Extensive load of somatic CNVs in the human placenta

Laura Kasak[1], Kristiina Rull[1,2,3], Pille Vaas[2,3], Pille Teesalu[2,3], Maris Laan[1*]

[1]Human Molecular Genetics Research Group, Institute of Molecular and Cell Biology, University of Tartu, Riia 23 St., Tartu 51010, Estonia

[2]Department of Obstetrics and Gynaecology, University of Tartu, Puusepa St. 8, Tartu 51014, Estonia

[3]Women's Clinic of Tartu University Hospital, Puusepa St. 8, Tartu 51014, Estonia

[*]Corresponding author: maris.laan@ut.ee

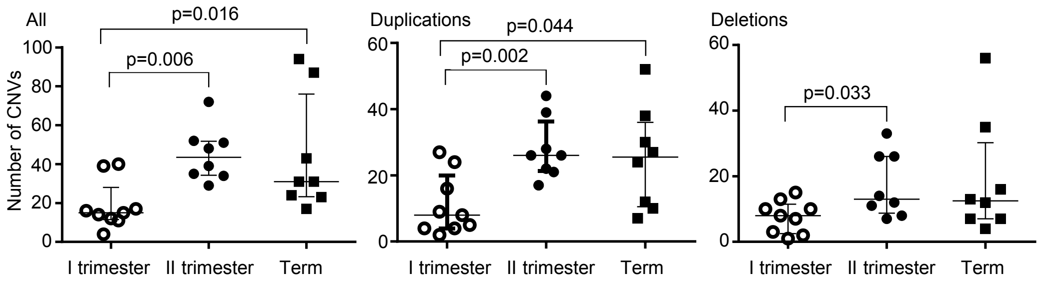

Supplementary Figure 1. Gradient of increasing number of CNVs from the first trimester towards term pregnancy. P-values were calculated by Welch two-sample t-test/ Wilcoxon rank sum test. Error bars show medians with interquartile range.

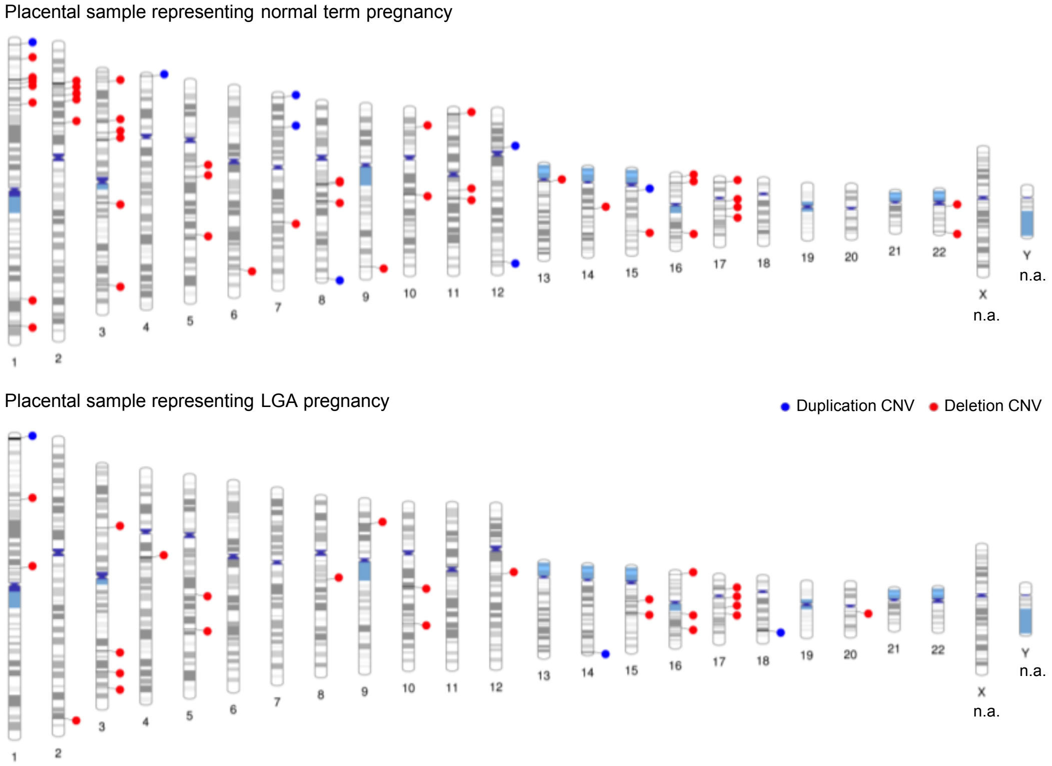

Supplementary Figure 2. Experimental validation of CNVs determined by SNP array (Illumina HumanOmniExpress Beadchip) using DNA hybridization-based aCGH (Agilent SurePrint G3 Human CGH 2x400K array). Blue (duplications) and red (deletions) dots indicate genomic locations of the CNVs identified by both methodological approaches in the placental genome for the two validation samples representing a normal term (vaginal delivery; XY karyotype) and an LGA (large-for-gestational age) pregnancy (caesarean section; XY karyotype). aCGH confirmed 56% (53/94) and 49% (28/57) of all CNVs including all inherited CNVs (2 and 3, respectively) identified by the SNP array in the discovery analysis. n.a, not analysed.

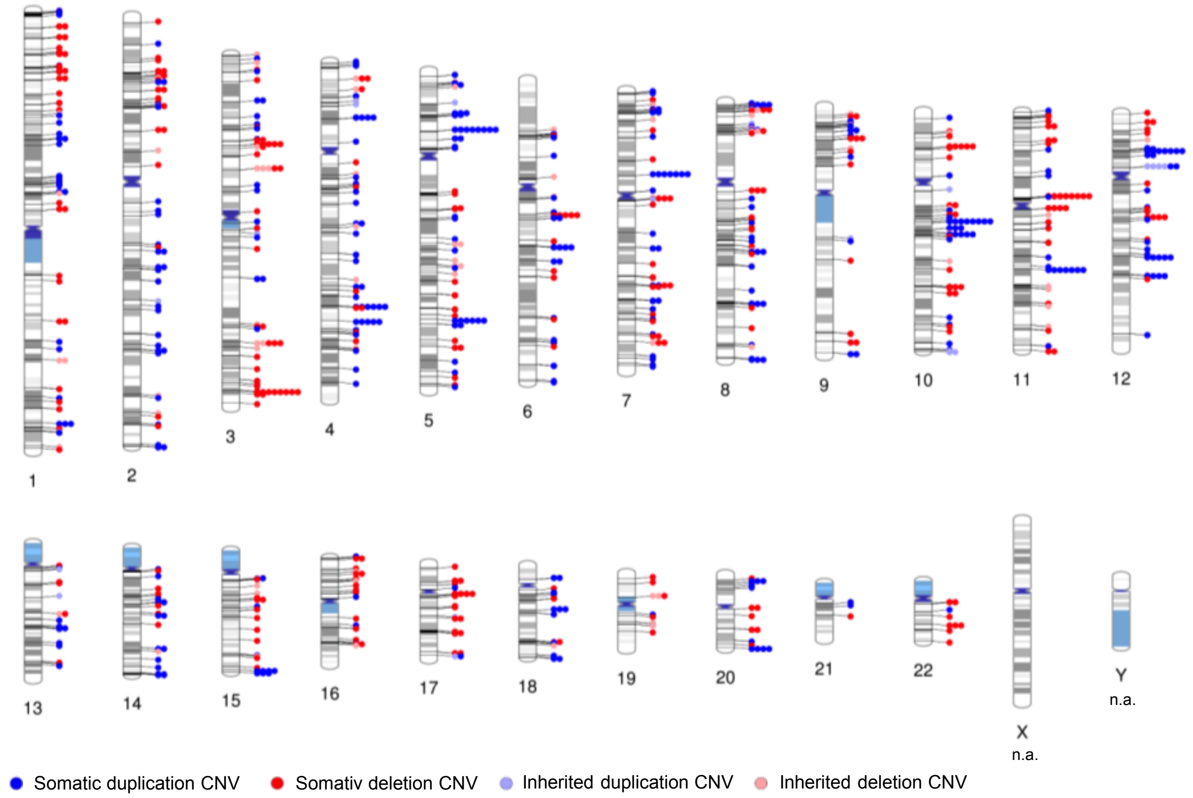

Supplementary Figure 3. Chromosomal distribution of all identified placental CNVs. Blue (somatic duplications), red (somatic deletions), light blue (inherited duplications) and light red (inherited deletions) dots indicate genomic locations of all placental CNVs detected by SNP array; http://visualization.ritchielab.psu.edu/phenograms/plot. n.a, not analysed.

Supplementary Table 1. CNVs detected in placental DNA samples

		I trimester	II trimester	Term<br>Normal term	SGA	LGA	PE	GD	Total
**No of samples**		9	8	8	8	8	8	8	57
**No of CNVs per sample**	all	18.6 (15.0)	45.0 (43.5)	43.6 (31.0)	18.3 (17.0)	32.6 (19.0)	23.9 (22.0)	18.8 (16.0)	28.5 (23.0)
	duplication	10.9 (8.0)	27.9 (26.0)	25.0 (25.5)	10.8 (7.0)	11.6 (9.5)	10.3 (10.0)	11.4 (8.5)	15.3 (12.0)
	deletion	7.7 (8.0)	17.1 (13.0)	18.6 (12.5)	7.5 (7.5)	21.0 (10.0)	13.6 (12.5)	7.4 (7.5)	13.2 (9.0)
**Cumulative span of all CNVs per sample, Mb**	all	2.2 (1.8)	5.2 (5.1)	5.7 (4.4)	1.5 (1.1)	5.1 (2.2)	3.7 (3.3)	1.6 (2.0)	3.6 (2.4)
	duplication	1.6 (1.7)	3.6 (3.2)	3.0 (3.5)	1.0 (0.9)	2.1 (1.5)	1.6 (1.2)	1.1 (1.0)	2.0 (1.4)
	deletion	0.6 (0.3)	1.6 (1.4)	2.7 (1.3)	0.5 (0.3)	3.0 (0.7)	2.2 (1.6)	0.5 (0.4)	1.5 (0.7)
**CNV length across samples, kb**	all	119.4 (54.9)	114.8 (64.3)	130.5 (79.9)	83.1 (52.8)	156.6 (97.5)	156.2 (85.3)	85.8 (42.5)	124.7 (70.4)
	duplication	150.4 (80.3)	129.7 (67.4)	120.7 (74.5)	94.7 (73.4)	183.6 (81.8)	152.9 (84.1)	95.2 (58.4)	130.8 (74.9)
	deletion	75.5 (26.9)	90.6 (58.8)	143.5 (90.7)	66.4 (26.5)	141.6 (106.9)	158.8 (93.3)	71.3 (13.3)	117.6 (63.8)
**CNV size range, kb**	all	0.7; 1,494.6	0.9; 1,700.1	0.3; 1,814.4	0.6; 464.0	0.5; 1,798.8	0.3; 1,670.6	0.9; 1,480.7	0.3; 1,814.4
	duplication	8.5; 1,494.6	4.0; 1,700.1	2.2; 1,814.4	2.6; 413.9	3.6; 1,798.8	6.2; 1,670.6	2.4; 815.1	2.2; 1,814.4
	deletion	0.7; 537.5	0.9; 482.4	0.3; 784.5	0.6; 464.0	0.5; 729.4	0.3; 1,105.6	0.9; 1,480.7	0.3; 1,480.7
**Ratio (loss/gain)**		0.7 (1.0)	0.6 (0.5)	0.7 (0.5)	0.7 (1.1)	1.8 (1.1)	1.3 (1.3)	0.6 (0.9)	0.9 (0.8)

Data are given as means (medians), except when indicated otherwise. No, number; SGA, small-for-gestational age; LGA, large-for-gestational age; PE, preeclampsia; GD, gestational diabetes.
Refer to Figure 2 for statistical testing of CNVs in placental compared to parental blood DNA samples.

Supplementary Table 2. CNVs detected in parental blood DNA samples

		I trimester (mother)	II trimester (mother)	Term (mother + father) Normal term	SGA	LGA	PE	GD	Total
**No of samples**		8	7	16	15	15	16	16	93
**No of CNVs per sample**	all	10.4 (10.0)	9.1 (9.0)	8.6 (9.5)	8.5 (7.0)	9.1 (9.0)	9.4 (10.0)	11.3 (10.0)	9.5 (9.0)
	duplication	3.0 (3.0)	2.0 (2.0)	2.3 (2.0)	2.1 (2.0)	2.7 (2.0)	2.8 (3.0)	3.5 (3.0)	2.7 (3.0)
	deletion	7.4 (6.5)	7.1 (7.0)	6.3 (7.0)	6.3 (6.0)	6.4 (6.0)	6.6 (7.0)	7.8 (7.0)	6.8 (7.0)
**Cumulative span of all CNVs per sample, Mb**	all	0.8 (0.5)	0.8 (0.5)	0.6 (0.7)	0.4 (0.4)	0.9 (0.7)	1.1 (1.1)	1.1 (0.7)	0.8 (0.6)
	duplication	0.5 (0.2)	0.6 (0.4)	0.3 (0.2)	0.2 (0.1)	0.6 (0.2)	0.7 (0.6)	0.6 (0.3)	0.5 (0.3)
	deletion	0.3 (0.3)	0.2 (0.2)	0.3 (0.2)	0.3 (0.2)	0.3 (0.3)	0.4 (0.3)	0.5 (0.3)	0.3 (0.3)
**CNV length across samples, kb**	all	74.2 (30.3)	87.8 (16.5)	71.5 (21.5)	50.6 (22.8)	99.9 (30.7)	120.9 (22.4)	95.5 (23.5)	87.8 (23.0)
	duplication	155.5 (75.3)	318.5 (187.4)	134.2 (83.2)	81.3 (40.0)	220.6 (73.2)	248.7 (69.7)	174.6 (51.4)	183.8 (68.3)
	deletion	41.2 (16.5)	23.2 (12.1)	48.3 (16.2)	40.2 (21.1)	48.4 (15.6)	66.7 (16.1)	59.8 (19.3)	49.8 (16.5)
**CNV size range, kb**	all	1.4; 1,298.5	0.3; 1,289.1	0.3; 539.5	1.3; 610.6	0.3; 2,039.5	0.3; 2,392.4	0.3; 1,420.2	0.3; 2,392.4
	duplication	6.7; 1,298.5	28.4; 1,289.1	2.2; 539.5	10.3; 610.6	2.5; 2,039.5	2.2; 2,392.4	2.5; 1,420.2	2.2; 2,392.4
	deletion	1.4; 327.9	0.3; 131.0	0.3; 477.2	1.3; 267.0	0.3; 948.6	0.3; 1,629.9	0.3; 1,391.2	0.3; 1,629.9
**Ratio (loss/gain)**		2.5 (2.2)	3.6 (3.5)	2.7 (3.5)	3.0 (3.0)	2.3 (3.0)	2.4 (2.3)	2.2 (2.3)	2.5 (2.3)

Data are given as means (medians), except when indicated otherwise. No, number; SGA, small-for-gestational age; LGA, large-for-gestational age; PE, preeclampsia; GD, gestational diabetes.
Refer to Figure 2 for statistical testing of CNVs in placental compared to parental blood DNA samples.

Supplementary Table 3. Copy number variants identified based on Illumina HumanOmniExpress array genotyping and CNV calling in placental (n=57), maternal and paternal blood DNA (n=93). Genomic coordinates are given according to hg19.

a. **Placental CNVs**

Chr	Start	End	Length	Group	Type	Inherited/somatic
1	1138912	1288823	149911	LGA	Dupl	somatic
1	1647686	1664019	16333	LGA	Del	somatic
1	1852484	3651318	1798834	LGA	Dupl	somatic
1	1967953	3668004	1700051	II_trimester	Dupl	somatic or paternally inherited*
1	1987802	3531594	1543792	LGA	Dupl	somatic
1	2307481	3654746	1347265	II_trimester	Dupl	somatic or paternally inherited*
1	2769542	3657759	888217	II_trimester	Dupl	somatic or paternally inherited*
1	3423467	3691528	268061	normal term	Dupl	somatic
1	9970779	10094313	123534	II_trimester	Del	somatic or paternally inherited*
1	9983329	10257166	273837	normal term	Del	somatic
1	9983329	10338598	355269	LGA	Del	somatic
1	9983329	10094313	110984	II_trimester	Del	somatic or paternally inherited*
1	9995102	10338598	343496	PE	Del	somatic
1	10008221	10193821	185600	PE	Del	somatic
1	10009925	10120793	110868	LGA	Del	somatic
1	10009925	10265606	255681	GD	Del	somatic
1	10651460	10874522	223062	II_trimester	Dupl	somatic or paternally inherited*
1	12911200	12951774	40574	SGA	Del	somatic or paternally inherited*
1	12911200	12951774	40574	LGA	Del	somatic or paternally inherited*
1	15783771	15796808	13037	normal term	Del	maternally inherited
1	16089356	16212219	122863	LGA	Del	somatic
1	16089356	16207862	118506	PE	Del	somatic
1	22054427	22127991	73564	LGA	Del	somatic
1	24206984	24264243	57259	LGA	Del	somatic
1	24901120	25121934	220814	normal term	Del	somatic
1	25598275	25655537	57262	II_trimester	Del	maternally inherited
1	25598275	25655537	57262	I_trimester	Del	maternally inherited
1	25598275	25655537	57262	normal term	Del	somatic
1	25598275	25655537	57262	normal term	Del	somatic
1	25598275	25655537	57262	II_trimester	Del	somatic or paternally inherited*
1	29004745	29038609	33864	I_trimester	Del	somatic or paternally inherited*
1	31504962	31682312	177350	II_trimester	Del	somatic or paternally inherited*
1	32031671	32266025	234354	LGA	Dupl	somatic
1	32277198	32595114	317916	normal term	Del	somatic
1	32904610	33092931	188321	normal term	Del	somatic
1	32993521	33100814	107293	LGA	Del	somatic
1	35107269	35110442	3173	SGA	Del	somatic
1	35107269	35110442	3173	GD	Del	somatic
1	35674310	35695273	20963	normal term	Del	somatic
1	39350742	39705874	355132	normal term	Del	somatic
1	39350742	39528071	177329	LGA	Del	somatic

Chr	Start	End	Length	Group	Type	Inherited/somatic
1	39492462	39545269	52807	II_trimester	Del	somatic or paternally inherited*
1	39498491	39550621	52130	II_trimester	Del	somatic or paternally inherited*
1	47794591	47806157	11566	normal term	Del	somatic
1	53135933	53459180	323247	normal term	Del	somatic
1	53173052	53218380	45328	normal term	Del	somatic
1	53180565	53374666	194101	LGA	Del	somatic
1	57057316	57088604	31288	normal term	Del	somatic
1	59007636	59086221	78585	SGA	Del	maternally inherited
1	60244033	60338776	94743	SGA	Dupl	somatic
1	64320736	64462582	141846	normal term	Dupl	somatic
1	70571471	70587360	15889	normal term	Dupl	somatic
1	72993809	73096321	102512	GD	Dupl	somatic
1	73012228	73096321	84093	II_trimester	Dupl	parental data not available
1	73378037	73482160	104123	normal term	Dupl	somatic
1	73378037	73482160	104123	normal term	Dupl	somatic
1	73386470	73482160	95690	normal term	Dupl	somatic
1	73390986	73482160	91174	II_trimester	Dupl	somatic or paternally inherited*
1	76342180	76383297	41117	SGA	Dupl	somatic
1	81212467	81386925	174458	II_trimester	Dupl	somatic or paternally inherited*
1	86402185	86404202	2017	I_trimester	Del	somatic or paternally inherited*
1	94352380	94372877	20497	SGA	Dupl	somatic
1	94867056	94986888	119832	LGA	Dupl	somatic
1	96321437	96490962	169525	normal term	Dupl	somatic
1	96463024	96490962	27938	II_trimester	Dupl	somatic or paternally inherited*
1	96469814	96490962	21148	II_trimester	Dupl	somatic or paternally inherited*
1	96469814	96490962	21148	I_trimester	Dupl	somatic or paternally inherited*
1	97542236	97583471	41235	II_trimester	Dupl	somatic or paternally inherited*
1	97542797	97588913	46116	normal term	Dupl	somatic
1	97870315	98357743	487428	II_trimester	Dupl	somatic or paternally inherited*
1	98057444	98075762	18318	I_trimester	Dupl	somatic or paternally inherited*
1	98069671	98348884	279213	normal term	Dupl	somatic
1	98203820	98357743	153923	PE	Dupl	somatic
1	98229591	98371448	141857	PE	Dupl	somatic
1	98292912	98316285	23373	normal term	Dupl	somatic
1	98292912	98316285	23373	II_trimester	Dupl	somatic or paternally inherited*
1	99770075	99809451	39376	normal term	Dupl	somatic
1	99783335	99809451	26116	SGA	Dupl	somatic
1	99783335	99800813	17478	II_trimester	Dupl	somatic or paternally inherited*
1	103161803	103508275	346472	SGA	Dupl	somatic
1	103165230	103467615	302385	normal term	Dupl	somatic
1	103328723	103548496	219773	normal term	Dupl	somatic
1	103328723	103508275	179552	SGA	Dupl	somatic
1	103342391	103551498	209107	GD	Dupl	somatic
1	103342391	103467615	125224	I_trimester	Dupl	somatic or paternally inherited*
1	103348100	103379917	31817	II_trimester	Dupl	somatic or paternally inherited*
1	104146557	104211046	64489	normal term	Del	maternally inherited
1	104146557	104155944	9387	I_trimester	Del	maternally inherited

Chr	Start	End	Length	Group	Type	Inherited/somatic
1	109503656	109605378	101722	II_trimester	Del	somatic or paternally inherited*
1	109549512	109605378	55866	LGA	Del	somatic
1	113023979	113040420	16441	normal term	Del	somatic
1	113023979	113046394	22415	II_trimester	Del	somatic or paternally inherited*
1	113026556	113040420	13864	normal term	Del	somatic
1	113026556	113040420	13864	GD	Del	somatic
1	149039031	149388389	349358	normal term	Del	somatic
1	149039031	149376652	337621	II_trimester	Del	somatic or paternally inherited*
1	152231425	152259742	28317	PE	Del	somatic
1	153674703	153696665	21962	II_trimester	Del	somatic or paternally inherited*
1	153989774	154128128	138354	II_trimester	Del	somatic or paternally inherited*
1	172106805	172185028	78223	I_trimester	Dupl	somatic or paternally inherited*
1	174797867	174801639	3772	PE	Del	somatic
1	174797867	174801639	3772	PE	Del	somatic
1	186281400	186441585	160185	SGA	Dupl	somatic
1	190416297	190476831	60534	normal term	Dupl	somatic
1	190416297	190492543	76246	LGA	Dupl	somatic or paternally inherited*
1	194299382	194334907	35525	II_trimester	Dupl	somatic or paternally inherited*
1	194299382	194334907	35525	I_trimester	Dupl	somatic or paternally inherited*
1	196823299	196855303	32004	SGA	Del	paternally inherited
1	196823299	196843710	20411	LGA	Del	paternally inherited
1	196823299	196855303	32004	I_trimester	Del	somatic or paternally inherited*
1	201239651	201301111	61460	II_trimester	Dupl	somatic or paternally inherited*
1	212928156	213088494	160338	normal term	Del	somatic
1	212986554	213020221	33667	SGA	Del	paternally inherited
1	212997385	213011811	14426	I_trimester	Del	maternally inherited
1	212997385	213020221	22836	normal term	Del	somatic
1	218003361	218019292	15931	normal term	Dupl	somatic
1	220197624	220314945	117321	LGA	Del	somatic
1	224273311	224570283	296972	LGA	Del	somatic
1	224273311	224570283	296972	II_trimester	Del	somatic or paternally inherited*
1	224278988	224570283	291295	PE	Del	somatic
1	232575539	232607762	32223	normal term	Dupl	somatic
1	232575539	232584061	8522	PE	Dupl	somatic
1	232575539	232604178	28639	PE	Dupl	somatic
1	232575539	232607762	32223	II_trimester	Dupl	somatic or paternally inherited*
1	235248412	235854739	606327	LGA	Del	somatic
1	235268017	235656128	388111	normal term	Del	somatic
1	236984561	237128380	143819	LGA	Del	maternally inherited
1	237584925	237614434	29509	LGA	Dupl	somatic
1	237584925	237617757	32832	I_trimester	Dupl	somatic or paternally inherited*
1	245636915	245647726	10811	normal term	Del	paternally inherited
1	247145690	247179076	33386	LGA	Del	somatic
2	4213378	4222144	8766	normal term	Del	somatic
2	16726739	16752999	26260	LGA	Dupl	somatic
2	24662502	24672179	9677	LGA	Del	somatic
2	26117455	26385849	268394	PE	Del	somatic

Chr	Start	End	Length	Group	Type	Inherited/somatic
2	32112652	32758353	645701	PE	Del	somatic
2	32145414	32969116	823702	PE	Del	somatic
2	32145414	32611512	466098	PE	Del	somatic
2	32148619	32933095	784476	normal term	Del	somatic
2	32148619	32612647	464028	SGA	Del	somatic or paternally inherited*
2	34042593	34048462	5869	LGA	Del	somatic
2	34678045	34726904	48859	normal term	Del	somatic
2	34678045	34726904	48859	LGA	Del	somatic
2	34678045	34726904	48859	I_trimester	Del	somatic or paternally inherited*
2	34699812	34726904	27092	II_trimester	Del	parental data not available
2	34699812	34726904	27092	SGA	Del	somatic
2	34699812	34726904	27092	LGA	Del	somatic
2	34699812	34726904	27092	II_trimester	Del	somatic or paternally inherited*
2	34699812	34726904	27092	II_trimester	Del	somatic or paternally inherited*
2	35819686	36087642	267956	SGA	Del	paternally inherited
2	37122552	37290658	168106	normal term	Del	somatic
2	38295493	38303094	7601	PE	Dupl	somatic
2	38295493	38303094	7601	GD	Dupl	somatic
2	38296889	38303094	6205	PE	Dupl	somatic
2	40839463	40896385	56922	II_trimester	Dupl	somatic or paternally inherited*
2	40846875	40884332	37457	II_trimester	Dupl	somatic or paternally inherited*
2	42615496	42895487	279991	normal term	Del	somatic
2	42690479	42881534	191055	LGA	Del	somatic
2	42690479	42881534	191055	PE	Del	somatic
2	47494799	47629897	135098	PE	Del	somatic
2	47494799	47643456	148657	II_trimester	Del	somatic or paternally inherited*
2	47563573	47643456	79883	normal term	Del	somatic
2	49535856	49537795	1939	GD	Del	somatic
2	51123048	51212751	89703	normal term	Dupl	somatic
2	51136802	51212751	75949	GD	Dupl	somatic
2	51279394	51294096	14702	GD	Dupl	somatic
2	51889503	51928206	38703	GD	Dupl	somatic
2	51911211	51928206	16995	normal term	Dupl	somatic
2	51926599	51926904	305	PE	Del	maternally/paternally inherited
2	51926599	51926904	305	normal term	Del	somatic
2	52324160	52434608	110448	SGA	Dupl	somatic
2	52326591	52397517	70926	II_trimester	Dupl	parental data not available
2	52326591	52369737	43146	I_trimester	Dupl	somatic or paternally inherited*
2	52338106	52369737	31631	PE	Dupl	somatic
2	52338106	52369737	31631	I_trimester	Dupl	somatic or paternally inherited*
2	54187868	54280129	92261	II_trimester	Del	somatic or paternally inherited*
2	65328694	65480620	151926	normal term	Del	somatic
2	65328694	65490659	161965	LGA	Del	somatic
2	76941049	76949101	8052	LGA	Del	maternally inherited
2	82188494	82351371	162877	II_trimester	Dupl	somatic or paternally inherited*
2	85131734	85339839	208105	LGA	Del	somatic
2	105690136	105711904	21768	I_trimester	Dupl	parental data not available

Chr	Start	End	Length	Group	Type	Inherited/somatic
2	105690136	105711904	21768	GD	Dupl	somatic
2	105690136	105711904	21768	II_trimester	Dupl	somatic or paternally inherited*
2	105690136	105711904	21768	II_trimester	Dupl	somatic or paternally inherited*
2	105690136	105711904	21768	I_trimester	Dupl	somatic or paternally inherited*
2	110863908	110958329	94421	II_trimester	Dupl	somatic or paternally inherited*
2	110885620	110958329	72709	normal term	Dupl	somatic
2	110885620	110905664	20044	I_trimester	Dupl	somatic or paternally inherited*
2	110889108	110958329	69221	I_trimester	Dupl	parental data not available
2	113041461	113053061	11600	GD	Dupl	somatic
2	123801083	123878592	77509	I_trimester	Dupl	maternally inherited
2	128388861	128410169	21308	normal term	Dupl	somatic
2	129641434	129645321	3887	II_trimester	Del	somatic or paternally inherited*
2	129777555	129785399	7844	PE	Del	somatic
2	129784722	129785399	677	I_trimester	Del	somatic or paternally inherited*
2	132343339	132509621	166282	GD	Dupl	somatic
2	132343339	132514741	171402	GD	Dupl	somatic
2	132343339	132509621	166282	I_trimester	Dupl	somatic or paternally inherited*
2	132343339	132496366	153027	I_trimester	Dupl	somatic or paternally inherited*
2	132352058	132509621	157563	II_trimester	Dupl	parental data not available
2	132352058	132649834	297776	II_trimester	Dupl	somatic or paternally inherited*
2	141125405	141154077	28672	PE	Dupl	somatic
2	141125405	141154077	28672	GD	Dupl	somatic
2	142592559	142639631	47072	II_trimester	Dupl	parental data not available
2	142592559	142668576	76017	GD	Dupl	somatic
2	142794718	142868146	73428	SGA	Dupl	somatic
2	149164048	149170115	6067	LGA	Del	somatic
2	149206943	149276546	69603	PE	Dupl	somatic
2	149206943	149283070	76127	II_trimester	Dupl	somatic or paternally inherited*
2	154701808	155115686	413878	I_trimester	Dupl	parental data not available
2	160291593	160569276	277683	PE	Dupl	maternally inherited
2	163005157	163243108	237951	I_trimester	Dupl	somatic or paternally inherited*
2	163194668	163243108	48440	PE	Dupl	somatic
2	165526338	165588818	62480	GD	Dupl	somatic
2	176769442	176809605	40163	II_trimester	Dupl	somatic or paternally inherited*
2	179390647	179668850	278203	normal term	Dupl	somatic
2	179479741	179668850	189109	II_trimester	Dupl	somatic or paternally inherited*
2	179490954	179668850	177896	normal term	Dupl	somatic
2	179490954	179668850	177896	II_trimester	Dupl	somatic or paternally inherited*
2	184797826	184802609	4783	I_trimester	Del	somatic or paternally inherited*
2	185428946	185564834	135888	SGA	Dupl	somatic
2	188156578	188684507	527929	II_trimester	Dupl	somatic or paternally inherited*
2	188256157	188554359	298202	PE	Dupl	somatic
2	188256157	188554359	298202	GD	Dupl	somatic
2	188256157	188554359	298202	II_trimester	Dupl	somatic or paternally inherited*
2	188388310	188545602	157292	GD	Dupl	somatic
2	189757531	189874459	116928	PE	Dupl	somatic
2	189833034	189874459	41425	II_trimester	Dupl	somatic or paternally inherited*

Chr	Start	End	Length	Group	Type	Inherited/somatic
2	191749035	191800904	51869	II_trimester	Dupl	somatic or paternally inherited*
2	198603121	198631713	28592	II_trimester	Del	somatic or paternally inherited*
2	199166382	199185440	19058	I_trimester	Dupl	parental data not available
2	208355808	208357837	2029	II_trimester	Del	somatic or paternally inherited*
2	213187034	213191389	4355	II_trimester	Del	maternally inherited
2	213635052	213649587	14535	LGA	Del	paternally inherited
2	214884986	214895245	10259	SGA	Dupl	somatic
2	223563574	223588671	25097	GD	Del	paternally inherited
2	223563574	223588671	25097	II_trimester	Del	somatic or paternally inherited*
2	225473263	225553128	79865	LGA	Del	somatic
2	229915119	229922254	7135	LGA	Dupl	somatic
2	230763690	230866297	102607	LGA	Del	somatic
2	230822296	230959540	137244	LGA	Del	somatic
2	241491444	241541639	50195	normal term	Dupl	somatic
2	242634818	243048760	413942	SGA	Dupl	somatic or paternally inherited*
2	242710662	243048760	338098	PE	Dupl	somatic
2	242710662	243048760	338098	PE	Dupl	somatic
2	242710662	243048760	338098	II_trimester	Dupl	somatic or paternally inherited*
2	242710935	243048760	337825	LGA	Dupl	somatic
2	242795350	243048760	253410	LGA	Dupl	somatic
3	857325	1067598	210273	GD	Del	maternally inherited
3	3189279	3208732	19453	GD	Dupl	somatic
3	5400168	5438374	38206	normal term	Del	maternally inherited
3	9540323	9660064	119741	normal term	Del	somatic
3	10382638	10512428	129790	normal term	Dupl	somatic
3	15371003	15450399	79396	LGA	Del	somatic
3	26843377	26881106	37729	GD	Dupl	somatic
3	26843377	26898377	55000	GD	Dupl	somatic
3	26843377	26881106	37729	II_trimester	Dupl	somatic or paternally inherited*
3	29633157	29693244	60087	II_trimester	Dupl	somatic or paternally inherited*
3	29638641	29700225	61584	II_trimester	Dupl	parental data not available
3	35678494	35762320	83826	normal term	Dupl	somatic
3	37979882	37986249	6367	II_trimester	Del	maternally inherited
3	40223615	40249048	25433	GD	Dupl	somatic
3	40223615	40249048	25433	II_trimester	Dupl	somatic or paternally inherited*
3	40223615	40249048	25433	II_trimester	Dupl	somatic or paternally inherited*
3	40223615	40249244	25629	I_trimester	Dupl	somatic or paternally inherited*
3	41920334	42067669	147335	normal term	Del	somatic
3	42676265	42726064	49799	normal term	Dupl	somatic
3	48606701	48628689	21988	normal term	Dupl	somatic
3	49074502	49129625	55123	normal term	Del	somatic
3	49074502	49129625	55123	LGA	Del	somatic
3	51457332	51616334	159002	normal term	Del	somatic
3	51457332	51616334	159002	normal term	Del	somatic
3	51457332	51616334	159002	LGA	Del	somatic
3	51457332	51697493	240161	LGA	Del	somatic
3	51457332	51616334	159002	II_trimester	Del	somatic or paternally inherited*

Chr	Start	End	Length	Group	Type	Inherited/somatic
3	51457332	51655856	198524	II_trimester	Del	somatic or paternally inherited*
3	51457332	51655856	198524	II_trimester	Del	somatic or paternally inherited*
3	51457332	51598254	140922	I_trimester	Del	somatic or paternally inherited*
3	51482527	51616334	133807	PE	Del	somatic
3	51482527	51577966	95439	SGA	Del	somatic
3	51482527	51616334	133807	SGA	Del	somatic
3	51482527	51616334	133807	LGA	Del	somatic
3	51482527	51623547	141020	PE	Del	somatic
3	51482527	51598254	115727	II_trimester	Del	somatic or paternally inherited*
3	51482527	51704721	222194	I_trimester	Del	somatic or paternally inherited*
3	51527117	51697493	170376	LGA	Del	somatic
3	51527117	51598254	71137	PE	Del	somatic
3	53033295	53035044	1749	LGA	Del	maternally/paternally inherited
3	53033295	53035044	1749	LGA	Del	somatic
3	57219344	57706177	486833	normal term	Del	somatic
3	65191847	65214685	22838	normal term	Del	maternally inherited
3	65191847	65214685	22838	PE	Del	paternally inherited
3	65191847	65202420	10573	GD	Del	maternally/paternally inherited
3	65191847	65215804	23957	II_trimester	Del	maternally inherited
3	65191847	65214685	22838	II_trimester	Del	maternally inherited
3	65191847	65215804	23957	I_trimester	Del	maternally inherited
3	65191847	65215804	23957	normal term	Del	somatic
3	65191847	65215804	23957	SGA	Del	somatic
3	65196123	65214685	18562	GD	Del	maternally inherited
3	74548437	74577921	29484	normal term	Dupl	somatic
3	77444432	77739202	294770	II_trimester	Dupl	somatic or paternally inherited*
3	77565186	77650799	85613	normal term	Dupl	somatic
3	77565186	77650799	85613	II_trimester	Dupl	somatic or paternally inherited*
3	77565186	77650799	85613	II_trimester	Dupl	somatic or paternally inherited*
3	83130081	83305448	175367	II_trimester	Dupl	somatic or paternally inherited*
3	83130081	83305448	175367	I_trimester	Dupl	somatic or paternally inherited*
3	89384565	89417171	32606	I_trimester	Del	maternally inherited
3	89384565	89417171	32606	LGA	Del	somatic
3	95146114	95310843	164729	GD	Dupl	somatic
3	98835121	98902911	67790	GD	Dupl	somatic
3	98946071	98947275	1204	SGA	Del	somatic
3	100351696	100441716	90020	PE	Dupl	paternally inherited
3	102529319	102609928	80609	SGA	Dupl	somatic
3	102529319	102651365	122046	II_trimester	Dupl	somatic or paternally inherited*
3	103097277	103126594	29317	LGA	Dupl	somatic or paternally inherited*
3	103097277	103140700	43423	II_trimester	Dupl	somatic or paternally inherited*
3	108962866	109182586	219720	normal term	Del	somatic
3	125886358	125973658	87300	II_trimester	Dupl	parental data not available
3	125887641	125978418	90777	I_trimester	Dupl	parental data not available
3	125887641	125978418	90777	normal term	Dupl	somatic
3	125887641	125978418	90777	GD	Dupl	somatic
3	125887641	125970734	83093	I_trimester	Dupl	somatic or paternally inherited*

Chr	Start	End	Length	Group	Type	Inherited/somatic
3	125887641	125970734	83093	I_trimester	Dupl	somatic or paternally inherited*
3	125897537	125973658	76121	SGA	Dupl	somatic
3	151514590	151546041	31451	SGA	Del	maternally inherited
3	152737318	152872542	135224	LGA	Del	somatic
3	152737318	152894141	156823	PE	Del	somatic
3	154054799	154191674	136875	GD	Dupl	somatic
3	162130691	162142475	11784	SGA	Del	maternally inherited
3	162130691	162142475	11784	PE	Del	paternally inherited
3	162130691	162142475	11784	LGA	Del	maternally inherited
3	162130691	162156517	25826	SGA	Del	somatic
3	162130691	162142475	11784	PE	Del	somatic
3	162130691	162142475	11784	PE	Del	somatic
3	162507286	162650452	143166	I_trimester	Dupl	somatic or paternally inherited*
3	165041116	165073006	31890	PE	Del	maternally/paternally inherited
3	169838000	170128750	290750	LGA	Del	somatic
3	169857007	170128750	271743	LGA	Del	somatic
3	176758477	177127453	368976	normal term	Del	somatic
3	183393037	183503633	110596	LGA	Del	somatic
3	183432982	183503633	70651	LGA	Del	somatic
3	185234542	185314612	80070	normal term	Del	somatic
3	186461157	186494421	33264	normal term	Del	somatic
3	189364423	189366762	2339	GD	Del	somatic
3	189738195	189739056	861	PE	Del	somatic
3	189738195	189739056	861	normal term	Del	somatic
3	189738195	189739056	861	normal term	Del	somatic
3	189738195	189739056	861	normal term	Del	somatic
3	189738195	189739056	861	SGA	Del	somatic
3	189738195	189739056	861	SGA	Del	somatic
3	189738195	189739056	861	PE	Del	somatic
3	189738195	189739056	861	GD	Del	somatic
3	189738195	189739056	861	II_trimester	Del	somatic or paternally inherited*
3	189738195	189739056	861	I_trimester	Del	somatic or paternally inherited*
3	189738195	189739056	861	I_trimester	Del	somatic or paternally inherited*
3	191065392	191069983	4591	II_trimester	Del	parental data not available
3	191065392	191069983	4591	normal term	Del	somatic
3	191065392	191069983	4591	GD	Del	somatic
3	191065392	191069983	4591	I_trimester	Del	somatic or paternally inherited*
3	196533319	196617207	83888	normal term	Del	somatic
3	196533319	196641105	107786	II_trimester	Del	somatic or paternally inherited*
4	821490	1054867	233377	normal term	Dupl	somatic
4	2235690	2304007	68317	LGA	Dupl	somatic
4	3186993	3875234	688241	normal term	Dupl	somatic
4	5074816	5110140	35324	I_trimester	Dupl	somatic or paternally inherited*
4	10396709	10400156	3447	normal term	Del	maternally inherited
4	10396709	10400156	3447	SGA	Del	somatic
4	10396709	10400156	3447	PE	Del	somatic
4	16413099	16428830	15731	SGA	Del	maternally inherited

Chr	Start	End	Length	Group	Type	Inherited/somatic
4	16413099	16428830	15731	PE	Del	somatic
4	20271773	20458688	186915	SGA	Dupl	somatic
4	20362716	20455955	93239	I_trimester	Dupl	parental data not available
4	20362716	20458688	95972	GD	Dupl	somatic
4	22962472	23563103	600631	GD	Dupl	maternally inherited
4	25028158	25475510	447352	GD	Dupl	maternally inherited
4	32345711	32558700	212989	II_trimester	Dupl	somatic or paternally inherited*
4	32398607	32558700	160093	normal term	Dupl	somatic
4	32428642	32558700	130058	normal term	Dupl	somatic
4	32428642	32558700	130058	SGA	Dupl	somatic
4	32428642	32558700	130058	SGA	Dupl	somatic
4	32428642	32558700	130058	GD	Dupl	somatic
4	32428642	32577729	149087	I_trimester	Dupl	somatic or paternally inherited*
4	32446724	32495165	48441	II_trimester	Dupl	parental data not available
4	32446724	32495165	48441	normal term	Dupl	somatic
4	34520176	34910052	389876	I_trimester	Dupl	somatic or paternally inherited*
4	45982196	46055477	73281	SGA	Dupl	somatic
4	57690713	57766052	75339	PE	Del	somatic
4	64138341	64150666	12325	GD	Del	maternally inherited
4	66110164	66340967	230803	LGA	Dupl	somatic
4	69678678	69706272	27594	normal term	Dupl	somatic
4	70592242	70600256	8014	PE	Dupl	somatic
4	70795699	71069004	273305	II_trimester	Dupl	parental data not available
4	70917031	71045118	128087	II_trimester	Dupl	somatic or paternally inherited*
4	71552305	72040650	488345	LGA	Del	somatic
4	74254678	74313193	58515	normal term	Dupl	somatic
4	80539001	80645990	106989	LGA	Dupl	somatic
4	92151187	92185714	34527	GD	Dupl	somatic
4	92151187	92185714	34527	GD	Dupl	somatic
4	93740408	93969165	228757	GD	Del	paternally inherited
4	96255088	96456602	201514	normal term	Dupl	somatic
4	96262520	96456602	194082	I_trimester	Dupl	somatic or paternally inherited*
4	99534928	99617267	82339	II_trimester	Dupl	parental data not available
4	100326402	100334943	8541	I_trimester	Dupl	parental data not available
4	102094764	102199215	104451	II_trimester	Dupl	somatic or paternally inherited*
4	102714886	102988188	273302	II_trimester	Dupl	somatic or paternally inherited*
4	108145936	108239396	93460	PE	Dupl	somatic
4	108195600	108239396	43796	PE	Dupl	somatic
4	108195600	108239396	43796	II_trimester	Dupl	somatic or paternally inherited*
4	111734136	111739292	5156	SGA	Dupl	somatic
4	122283522	122289862	6340	GD	Del	paternally inherited
4	122283522	122289862	6340	II_trimester	Del	somatic or paternally inherited*
4	122283522	122289862	6340	I_trimester	Del	somatic or paternally inherited*
4	126252650	126447899	195249	normal term	Dupl	somatic
4	126252650	126447899	195249	GD	Dupl	somatic
4	126252650	126280006	27356	II_trimester	Dupl	somatic or paternally inherited*
4	128723458	129029653	306195	LGA	Del	somatic

Chr	Start	End	Length	Group	Type	Inherited/somatic
4	131539495	131561136	21641	II_trimester	Dupl	somatic or paternally inherited*
4	132051076	132257490	206414	normal term	Dupl	somatic
4	137542931	137718455	175524	normal term	Dupl	somatic
4	137542931	137720486	177555	normal term	Dupl	somatic
4	137542931	137718455	175524	normal term	Dupl	somatic
4	137542931	137698875	155944	SGA	Dupl	somatic
4	137542931	137828866	285935	PE	Dupl	somatic
4	137542931	137714170	171239	GD	Dupl	somatic
4	137542931	137718455	175524	II_trimester	Dupl	somatic or paternally inherited*
4	137542931	137720486	177555	II_trimester	Dupl	somatic or paternally inherited*
4	137542931	137718455	175524	I_trimester	Dupl	somatic or paternally inherited*
4	137542931	137718455	175524	I_trimester	Dupl	somatic or paternally inherited*
4	137567044	137720486	153442	II_trimester	Dupl	parental data not available
4	138093809	138097449	3640	PE	Del	somatic
4	138093809	138097449	3640	GD	Del	somatic
4	146025645	146073143	47498	normal term	Dupl	somatic
4	146025645	146139896	114251	normal term	Dupl	somatic
4	146025645	146055117	29472	SGA	Dupl	somatic
4	146025645	146061560	35915	SGA	Dupl	somatic
4	146025645	146061560	35915	GD	Dupl	somatic
4	146025645	146082942	57297	II_trimester	Dupl	somatic or paternally inherited*
4	150634191	150845428	211237	PE	Dupl	somatic
4	151901433	152039963	138530	normal term	Del	somatic
4	152790580	152792791	2211	normal term	Del	somatic
4	152790580	152792791	2211	II_trimester	Del	somatic or paternally inherited*
4	153229867	153388854	158987	normal term	Dupl	somatic
4	156963692	156967385	3693	SGA	Del	somatic
4	161058055	161071059	13004	GD	Del	maternally inherited
4	161058055	161071059	13004	I_trimester	Del	somatic or paternally inherited*
4	161881000	161884036	3036	LGA	Del	somatic
4	162350385	162458688	108303	normal term	Dupl	somatic
4	168366518	168391333	24815	II_trimester	Dupl	somatic or paternally inherited*
4	172943891	172957428	13537	normal term	Dupl	somatic
4	180982097	181108510	126413	normal term	Dupl	somatic
4	182377628	182413696	36068	II_trimester	Dupl	somatic or paternally inherited*
5	3163438	3194690	31252	normal term	Dupl	somatic
5	3166087	3194690	28603	II_trimester	Dupl	parental data not available
5	3166087	3181008	14921	normal term	Dupl	somatic
5	7178644	7191074	12430	LGA	Del	paternally inherited
5	7179551	7191074	11523	normal term	Del	paternally inherited
5	7843211	7920843	77632	LGA	Dupl	somatic
5	7843211	7946262	103051	I_trimester	Dupl	somatic or paternally inherited*
5	7859433	7927160	67727	normal term	Dupl	somatic
5	7885226	7920843	35617	PE	Dupl	somatic
5	8836536	8862668	26132	normal term	Dupl	somatic
5	8836536	8860434	23898	LGA	Dupl	somatic
5	9902403	9924597	22194	PE	Del	maternally inherited

Chr	Start	End	Length	Group	Type	Inherited/somatic
5	9902403	9924597	22194	SGA	Del	maternally inherited
5	18799045	18895407	96362	normal term	Dupl	maternally inherited
5	24779565	24806889	27324	II_trimester	Dupl	parental data not available
5	24779565	24806889	27324	normal term	Dupl	somatic
5	24779565	24806889	27324	SGA	Dupl	somatic
5	24779565	24806889	27324	GD	Dupl	somatic
5	25676606	25737864	61258	GD	Dupl	somatic
5	25676606	25739983	63377	GD	Dupl	somatic
5	25687036	25723615	36579	normal term	Dupl	somatic
5	25687036	25739983	52947	SGA	Dupl	somatic
5	25698030	25723615	25585	LGA	Dupl	somatic or paternally inherited*
5	25976806	26196559	219753	GD	Dupl	somatic
5	34052480	34402152	349672	LGA	Dupl	somatic
5	34052480	34389859	337379	LGA	Dupl	somatic
5	34107121	34428293	321172	normal term	Dupl	somatic
5	34107121	34389859	282738	SGA	Dupl	somatic
5	34107121	34389859	282738	SGA	Dupl	somatic
5	34107121	34389859	282738	LGA	Dupl	somatic
5	34107121	34190148	83027	PE	Dupl	somatic
5	34107121	34452193	345072	PE	Dupl	somatic
5	34107121	34190148	83027	PE	Dupl	somatic
5	34107121	34428293	321172	PE	Dupl	somatic
5	34107121	34389859	282738	I_trimester	Dupl	somatic or paternally inherited*
5	34107121	34389859	282738	I_trimester	Dupl	somatic or paternally inherited*
5	39105593	39132459	26866	SGA	Dupl	somatic
5	39105593	39124016	18423	I_trimester	Dupl	somatic or paternally inherited*
5	39113637	39132459	18822	normal term	Dupl	somatic
5	39113637	39124016	10379	normal term	Dupl	somatic
5	40865863	40974004	108141	I_trimester	Del	somatic or paternally inherited*
5	43704556	44517392	812836	LGA	Dupl	somatic
5	44313150	44517392	204242	normal term	Dupl	somatic
5	44351153	44517392	166239	II_trimester	Dupl	somatic or paternally inherited*
5	44499291	44517392	18101	GD	Dupl	somatic
5	44499291	44517392	18101	LGA	Dupl	somatic or paternally inherited*
5	44499291	44517392	18101	I_trimester	Dupl	somatic or paternally inherited*
5	44513809	44517392	3583	LGA	Dupl	somatic
5	56215752	56259853	44101	I_trimester	Dupl	somatic or paternally inherited*
5	66348322	66403500	55178	II_trimester	Dupl	somatic or paternally inherited*
5	66348322	66406143	57821	I_trimester	Dupl	somatic or paternally inherited*
5	68826788	70307464	1480676	GD	Del	somatic
5	70305696	70307464	1768	normal term	Del	somatic
5	78421958	78532658	110700	normal term	Del	somatic
5	78421958	78629346	207388	PE	Del	somatic
5	78829154	78909324	80170	II_trimester	Del	somatic or paternally inherited*
5	78848000	78909324	61324	normal term	Del	somatic
5	78853921	78909324	55403	LGA	Del	somatic
5	83224173	83271937	47764	II_trimester	Dupl	parental data not available

Chr	Start	End	Length	Group	Type	Inherited/somatic
5	83224173	83260938	36765	PE	Dupl	somatic
5	84177145	84322004	144859	GD	Dupl	somatic
5	84177145	84281179	104034	I_trimester	Dupl	somatic or paternally inherited*
5	93788056	93864514	76458	II_trimester	Dupl	parental data not available
5	94191436	94239189	47753	normal term	Dupl	somatic
5	97047653	97111523	63870	LGA	Del	maternally inherited
5	97048466	97099320	50854	LGA	Del	maternally inherited
5	97048466	97111523	63057	GD	Del	paternally inherited
5	97048466	97099320	50854	II_trimester	Del	maternally inherited
5	97048466	97099320	50854	I_trimester	Del	maternally inherited
5	97933888	97939616	5728	I_trimester	Del	maternally inherited
5	97933888	97939616	5728	II_trimester	Del	somatic or paternally inherited*
5	99597529	99627951	30422	LGA	Del	maternally inherited
5	100604495	100611918	7423	normal term	Dupl	somatic
5	104025707	104845257	819550	I_trimester	Dupl	somatic or paternally inherited*
5	106211190	106256408	45218	SGA	Del	paternally inherited
5	109358981	109384306	25325	SGA	Del	maternally inherited
5	109358981	109384306	25325	SGA	Del	paternally inherited
5	110427347	110467237	39890	normal term	Dupl	somatic
5	113874136	113986223	112087	PE	Del	maternally inherited
5	118225616	118534517	308901	LGA	Del	somatic
5	124843659	124871683	28024	II_trimester	Dupl	somatic or paternally inherited*
5	124843659	124875058	31399	I_trimester	Dupl	somatic or paternally inherited*
5	125879549	126095011	215462	normal term	Del	somatic
5	125879549	126161748	282199	II_trimester	Del	somatic or paternally inherited*
5	125897227	126130100	232873	LGA	Del	somatic
5	125943743	126172194	228451	LGA	Del	somatic
5	125946011	126135382	189371	normal term	Del	somatic
5	133741881	134258183	516302	normal term	Del	somatic
5	133959630	134189243	229613	PE	Del	somatic
5	137537724	137631073	93349	PE	Del	somatic
5	140165568	140264639	99071	normal term	Dupl	somatic
5	140165568	140246287	80719	normal term	Dupl	somatic
5	140165568	140246511	80943	normal term	Dupl	somatic
5	140165568	140264639	99071	SGA	Dupl	somatic
5	140165568	140253787	88219	SGA	Dupl	somatic
5	140165568	140253787	88219	PE	Dupl	somatic
5	142762977	142780485	17508	normal term	Dupl	somatic
5	142767739	142786700	18961	LGA	Dupl	somatic
5	142767739	142786700	18961	PE	Dupl	somatic
5	142770134	142786700	16566	PE	Dupl	somatic
5	151514956	151518809	3853	GD	Del	somatic
5	155477866	155488438	10572	LGA	Del	somatic
5	155477866	155488438	10572	GD	Del	somatic
5	163348798	163357967	9169	LGA	Dupl	somatic
5	163348798	163357967	9169	SGA	Dupl	somatic or paternally inherited*
5	169460787	169472788	12001	PE	Dupl	somatic

Chr	Start	End	Length	Group	Type	Inherited/somatic
5	172395581	172562142	166561	LGA	Del	somatic
5	176541936	176700884	158948	LGA	Del	somatic
5	176569187	176724056	154869	LGA	Del	somatic
5	177661902	177747691	85789	LGA	Dupl	somatic
5	178557142	178785054	227912	II_trimester	Dupl	somatic or paternally inherited*
6	19045560	19048773	3213	I_trimester	Del	somatic or paternally inherited*
6	22677767	22701495	23728	II_trimester	Dupl	somatic or paternally inherited*
6	29094695	29168841	74146	LGA	Del	maternally inherited
6	31219869	31228971	9102	GD	Del	paternally inherited
6	31276609	31277987	1378	GD	Del	somatic
6	31276609	31277987	1378	II_trimester	Del	somatic or paternally inherited*
6	31281770	31285291	3521	SGA	Del	somatic
6	31360254	31454364	94110	PE	Dupl	paternally inherited
6	31682029	31706473	24444	II_trimester	Dupl	somatic or paternally inherited*
6	31682029	31706473	24444	II_trimester	Dupl	somatic or paternally inherited*
6	31892483	32070837	178354	normal term	Dupl	somatic
6	31895215	32044850	149635	LGA	Dupl	somatic
6	31919577	32017539	97962	PE	Dupl	somatic
6	31974848	31997053	22205	II_trimester	Del	somatic or paternally inherited*
6	31974848	31997053	22205	I_trimester	Del	somatic or paternally inherited*
6	32193219	32246156	52937	II_trimester	Del	somatic or paternally inherited*
6	32501715	32519004	17289	GD	Del	somatic
6	32501715	32519004	17289	I_trimester	Del	somatic or paternally inherited*
6	32638315	32640336	2021	GD	Del	somatic
6	33588146	33791515	203369	PE	Dupl	somatic
6	33601132	33794258	193126	PE	Dupl	somatic
6	33601132	33794258	193126	II_trimester	Dupl	somatic or paternally inherited*
6	33613344	33794258	180914	II_trimester	Dupl	somatic or paternally inherited*
6	33659793	33791515	131722	II_trimester	Dupl	somatic or paternally inherited*
6	43911551	44046143	134592	LGA	Dupl	somatic
6	45295124	45349509	54385	II_trimester	Dupl	somatic or paternally inherited*
6	55776584	56253790	477206	normal term	Del	paternally inherited
6	67017494	67044431	26937	PE	Del	paternally inherited
6	67017494	67044431	26937	I_trimester	Del	somatic or paternally inherited*
6	67095924	67270731	174807	II_trimester	Dupl	somatic or paternally inherited*
6	67621368	67656128	34760	LGA	Dupl	somatic
6	67621368	67656128	34760	II_trimester	Dupl	somatic or paternally inherited*
6	70919443	70923469	4026	II_trimester	Dupl	somatic or paternally inherited*
6	75825181	75840120	14939	PE	Dupl	somatic
6	75826134	75840120	13986	normal term	Dupl	somatic
6	75826134	75840120	13986	II_trimester	Dupl	somatic or paternally inherited*
6	77439969	77448731	8762	SGA	Del	somatic
6	77439969	77448731	8762	PE	Del	somatic
6	77439969	77448731	8762	PE	Del	somatic
6	77439969	77448731	8762	GD	Del	somatic
6	77439969	77448731	8762	GD	Del	somatic
6	77439969	77448731	8762	I_trimester	Del	somatic or paternally inherited*

Chr	Start	End	Length	Group	Type	Inherited/somatic
6	78972930	79029367	56437	normal term	Del	somatic
6	78972930	79029367	56437	II_trimester	Del	somatic or paternally inherited*
6	78972930	79029367	56437	II_trimester	Del	somatic or paternally inherited*
6	78972930	79029367	56437	II_trimester	Del	somatic or paternally inherited*
6	78972930	79029367	56437	I_trimester	Del	somatic or paternally inherited*
6	78975090	79029367	54277	normal term	Dupl	somatic
6	78975090	79047537	72447	normal term	Dupl	somatic
6	78979398	79042990	63592	normal term	Dupl	somatic
6	78979398	79078738	99340	LGA	Dupl	somatic
6	78979398	79029367	49969	I_trimester	Dupl	somatic or paternally inherited*
6	90476276	90631504	155228	LGA	Del	somatic
6	93752014	93859659	107645	normal term	Dupl	somatic
6	93950566	94120638	170072	PE	Dupl	somatic
6	93950566	94133927	183361	normal term	Dupl	somatic
6	93950566	94130024	179458	GD	Dupl	somatic
6	93950566	94130024	179458	SGA	Dupl	somatic or paternally inherited*
6	93950566	94120638	170072	II_trimester	Dupl	somatic or paternally inherited*
6	93950566	94120638	170072	II_trimester	Dupl	somatic or paternally inherited*
6	93950566	94120638	170072	I_trimester	Dupl	somatic or paternally inherited*
6	93956769	94130024	173255	II_trimester	Dupl	somatic or paternally inherited*
6	93959361	94130024	170663	II_trimester	Dupl	parental data not available
6	93959361	94117546	158185	SGA	Dupl	somatic
6	93959361	94120638	161277	SGA	Dupl	somatic
6	93959361	94117546	158185	GD	Dupl	somatic
6	93959361	94117546	158185	GD	Dupl	somatic
6	93959361	94130024	170663	II_trimester	Dupl	somatic or paternally inherited*
6	93961571	94117546	155975	normal term	Dupl	somatic
6	93961571	94117546	155975	normal term	Dupl	somatic
6	93961571	94120638	159067	LGA	Dupl	somatic
6	93961571	94120638	159067	GD	Dupl	somatic
6	93965265	94120638	155373	normal term	Dupl	somatic
6	93965265	94120638	155373	II_trimester	Dupl	somatic or paternally inherited*
6	101891573	101953555	61982	normal term	Dupl	somatic
6	101891573	101990259	98686	SGA	Dupl	somatic
6	101927337	101959098	31761	normal term	Dupl	somatic
6	107328319	107351798	23479	normal term	Del	somatic
6	110969521	111415640	446119	LGA	Del	somatic
6	111081133	111394638	313505	PE	Del	somatic
6	112135225	112203035	67810	I_trimester	Dupl	somatic or paternally inherited*
6	112152560	112170222	17662	I_trimester	Dupl	somatic or paternally inherited*
6	133732404	133847876	115472	PE	Dupl	somatic
6	133763905	133817456	53551	normal term	Dupl	somatic
6	134516019	134627600	111581	LGA	Del	somatic
6	139603744	139606731	2987	I_trimester	Del	somatic or paternally inherited*
6	146204066	146271476	67410	normal term	Dupl	somatic
6	146204066	146271476	67410	II_trimester	Dupl	somatic or paternally inherited*
6	147551381	147730324	178943	II_trimester	Dupl	somatic or paternally inherited*

Chr	Start	End	Length	Group	Type	Inherited/somatic
6	147566285	147723577	157292	normal term	Dupl	somatic
6	147568513	147709180	140667	GD	Dupl	somatic
6	147568513	147709180	140667	I_trimester	Dupl	somatic or paternally inherited*
6	147635547	147709180	73633	SGA	Dupl	somatic
6	149913867	150102785	188918	normal term	Del	somatic
6	160779134	160892241	113107	PE	Dupl	somatic
6	160784836	160911053	126217	LGA	Dupl	somatic
6	160790352	160854138	63786	PE	Dupl	somatic
6	160795738	160854138	58400	GD	Dupl	somatic
6	160795738	160872150	76412	II_trimester	Dupl	somatic or paternally inherited*
6	160796565	160854138	57573	II_trimester	Dupl	parental data not available
6	160802238	160897872	95634	normal term	Dupl	somatic
6	160802238	160865055	62817	II_trimester	Dupl	somatic or paternally inherited*
6	160810124	160897872	87748	I_trimester	Dupl	somatic or paternally inherited*
6	160810124	160865055	54931	I_trimester	Dupl	somatic or paternally inherited*
6	160812187	160865055	52868	normal term	Dupl	somatic
6	160812187	160854138	41951	II_trimester	Dupl	somatic or paternally inherited*
6	164186315	164214737	28422	I_trimester	Dupl	parental data not available
6	169334481	169387386	52905	normal term	Dupl	somatic
6	170070723	170140184	69461	II_trimester	Dupl	parental data not available
6	170070723	170140184	69461	I_trimester	Dupl	somatic or paternally inherited*
6	170099397	170140184	40787	I_trimester	Dupl	parental data not available
6	170099397	170162537	63140	SGA	Dupl	somatic
6	170101841	170140184	38343	I_trimester	Dupl	somatic or paternally inherited*
7	1473468	1536717	63249	normal term	Dupl	somatic
7	1882227	2212758	330531	normal term	Dupl	somatic
7	2649673	2703652	53979	LGA	Dupl	somatic
7	6316798	6422555	105757	normal term	Del	somatic
7	6339313	6447990	108677	II_trimester	Del	somatic or paternally inherited*
7	9128070	9229882	101812	normal term	Del	maternally inherited
7	11677088	11753281	76193	normal term	Dupl	somatic
7	11677840	11759464	81624	SGA	Dupl	somatic
7	11677840	11753281	75441	GD	Dupl	somatic
7	11677840	11753281	75441	LGA	Dupl	somatic or paternally inherited*
7	11678246	11759464	81218	GD	Dupl	somatic
7	11678246	11753281	75035	II_trimester	Dupl	somatic or paternally inherited*
7	12543120	12547917	4797	SGA	Del	somatic
7	13185882	13459064	273182	normal term	Dupl	somatic
7	13185882	13522169	336287	GD	Dupl	somatic
7	13190920	13511472	320552	normal term	Dupl	somatic
7	13429017	13445967	16950	PE	Dupl	somatic
7	13433240	13450842	17602	normal term	Dupl	somatic
7	13433240	13445967	12727	GD	Dupl	somatic
7	13439084	13520443	81359	LGA	Dupl	somatic
7	17425895	17458078	32183	normal term	Del	maternally inherited
7	23539546	23637416	97870	LGA	Del	somatic
7	27118974	27243221	124247	normal term	Dupl	somatic

Chr	Start	End	Length	Group	Type	Inherited/somatic
7	39962863	40314164	351301	LGA	Del	somatic
7	48310166	48318810	8644	II_trimester	Dupl	parental data not available
7	48310166	48318810	8644	PE	Dupl	somatic
7	48310166	48318810	8644	normal term	Dupl	somatic
7	48310166	48318810	8644	SGA	Dupl	somatic
7	48310166	48318810	8644	SGA	Dupl	somatic
7	48310166	48318810	8644	LGA	Dupl	somatic
7	48310166	48318810	8644	GD	Dupl	somatic
7	48310166	48318810	8644	GD	Dupl	somatic
7	48310166	48318810	8644	II_trimester	Dupl	somatic or paternally inherited*
7	48310166	48318810	8644	II_trimester	Dupl	somatic or paternally inherited*
7	57453615	57621951	168336	PE	Dupl	maternally inherited
7	57520410	57642390	121980	GD	Dupl	somatic
7	57520410	57787663	267253	II_trimester	Dupl	somatic or paternally inherited*
7	57520410	57642390	121980	II_trimester	Dupl	somatic or paternally inherited*
7	61994170	62203030	208860	I_trimester	Del	maternally inherited
7	61994170	62458262	464092	normal term	Del	somatic
7	61994170	62531632	537462	LGA	Del	somatic
7	61994170	62527743	533573	PE	Del	somatic
7	61994170	62458262	464092	PE	Del	somatic
7	61994170	62531632	537462	I_trimester	Del	somatic or paternally inherited*
7	62154874	62159926	5052	LGA	Del	somatic
7	62154874	62159926	5052	I_trimester	Del	somatic or paternally inherited*
7	62326772	62705018	378246	PE	Dupl	paternally inherited
7	65449716	65546062	96346	LGA	Del	somatic
7	65449716	65546062	96346	II_trimester	Del	somatic or paternally inherited*
7	80211423	80307224	95801	II_trimester	Dupl	parental data not available
7	80211423	80373728	162305	I_trimester	Dupl	somatic or paternally inherited*
7	80223996	80307224	83228	GD	Dupl	somatic
7	80709513	80739951	30438	II_trimester	Dupl	somatic or paternally inherited*
7	81737327	81824403	87076	II_trimester	Dupl	somatic or paternally inherited*
7	88560427	88582676	22249	normal term	Dupl	somatic
7	88560427	88582676	22249	PE	Dupl	somatic
7	88560427	88596821	36394	LGA	Dupl	somatic or paternally inherited*
7	88568549	88580316	11767	LGA	Dupl	somatic
7	89859977	89873767	13790	II_trimester	Dupl	somatic or paternally inherited*
7	97399028	97400870	1842	PE	Del	somatic
7	104786147	105205917	419770	normal term	Del	somatic
7	104896470	105076977	180507	II_trimester	Del	somatic or paternally inherited*
7	104972800	105073788	100988	normal term	Del	somatic
7	105036690	105291444	254754	PE	Del	somatic
7	109441794	109451230	9436	PE	Del	somatic
7	109441794	109451230	9436	normal term	Del	somatic
7	109441794	109451230	9436	normal term	Del	somatic
7	109441794	109451230	9436	PE	Del	somatic
7	109441794	109451230	9436	II_trimester	Del	somatic or paternally inherited*
7	109455705	109477177	21472	PE	Dupl	somatic

Chr	Start	End	Length	Group	Type	Inherited/somatic
7	109455705	109477177	21472	normal term	Dupl	somatic
7	109455705	109477177	21472	II_trimester	Dupl	somatic or paternally inherited*
7	110183622	110184985	1363	LGA	Del	somatic
7	110183622	110184985	1363	GD	Del	somatic
7	110183622	110184985	1363	II_trimester	Del	somatic or paternally inherited*
7	117994519	118160303	165784	SGA	Dupl	somatic
7	117994519	118160303	165784	LGA	Dupl	somatic
7	118263380	118572753	309373	normal term	Dupl	somatic
7	122785918	122894149	108231	PE	Dupl	somatic
7	122793182	122869067	75885	normal term	Dupl	somatic
7	122793182	122869067	75885	II_trimester	Dupl	somatic or paternally inherited*
7	122798636	122869067	70431	II_trimester	Dupl	parental data not available
7	125812615	125828396	15781	SGA	Del	somatic
7	128470837	128554093	83256	LGA	Dupl	somatic
7	129555547	129761082	205535	PE	Del	somatic
7	129579325	129676786	97461	LGA	Del	somatic
7	129598336	129710136	111800	I_trimester	Del	somatic or paternally inherited*
7	135214060	135238834	24774	II_trimester	Del	somatic or paternally inherited*
7	136980174	136994013	13839	LGA	Dupl	somatic
7	138066098	138079079	12981	SGA	Del	somatic
7	138066098	138082575	16477	PE	Del	somatic
7	138066098	138079079	12981	II_trimester	Del	somatic or paternally inherited*
7	141772628	141793419	20791	GD	Del	maternally inherited
7	141772628	141793419	20791	LGA	Del	somatic
7	141772628	141793419	20791	GD	Del	somatic
7	149410638	149583170	172532	normal term	Dupl	somatic
7	149420152	149563550	143398	LGA	Dupl	somatic
7	151062348	151185655	123307	LGA	Dupl	somatic
7	154201075	154203454	2379	GD	Dupl	somatic
7	155246891	155269627	22736	LGA	Dupl	somatic
8	1892069	1959336	67267	LGA	Dupl	somatic
8	2142267	2158537	16270	I_trimester	Del	maternally inherited
8	2781277	2833794	52517	SGA	Dupl	somatic
8	2781277	2833794	52517	PE	Dupl	somatic
8	2781277	2833794	52517	GD	Dupl	somatic
8	2781277	2827657	46380	I_trimester	Dupl	somatic or paternally inherited*
8	2784418	2827657	43239	II_trimester	Dupl	parental data not available
8	2784418	2827657	43239	normal term	Dupl	somatic
8	2784418	2891804	107386	normal term	Dupl	somatic
8	2784418	2827657	43239	SGA	Dupl	somatic
8	2784418	2833794	49376	PE	Dupl	somatic
8	2796810	2827657	30847	II_trimester	Dupl	somatic or paternally inherited*
8	2797776	2827657	29881	normal term	Dupl	somatic
8	2797776	2827657	29881	II_trimester	Dupl	somatic or paternally inherited*
8	2808265	2827657	19392	normal term	Dupl	somatic
8	2808265	2827657	19392	normal term	Dupl	somatic
8	2808265	2827657	19392	GD	Dupl	somatic

Chr	Start	End	Length	Group	Type	Inherited/somatic
8	2808265	2833794	25529	II_trimester	Dupl	somatic or paternally inherited*
8	2808265	2827657	19392	II_trimester	Dupl	somatic or paternally inherited*
8	2811241	2833794	22553	I_trimester	Dupl	parental data not available
8	3786543	3789272	2729	GD	Del	somatic
8	3984758	3987799	3041	I_trimester	Del	somatic or paternally inherited*
8	4272581	4285663	13082	normal term	Dupl	somatic
8	4272581	4278256	5675	LGA	Dupl	somatic
8	4272581	4285663	13082	LGA	Dupl	somatic
8	4272581	4290386	17805	II_trimester	Dupl	somatic or paternally inherited*
8	4272581	4285663	13082	I_trimester	Dupl	somatic or paternally inherited*
8	4803156	4819101	15945	II_trimester	Dupl	parental data not available
8	5535501	5547288	11787	SGA	Dupl	somatic
8	5542087	5551530	9443	normal term	Dupl	somatic
8	5563282	5587923	24641	PE	Del	maternally inherited
8	5599399	5605087	5688	SGA	Del	paternally inherited
8	5599399	5605087	5688	GD	Del	paternally inherited
8	5599399	5605087	5688	I_trimester	Del	maternally inherited
8	5599399	5607214	7815	normal term	Del	somatic
8	5599399	5605087	5688	SGA	Del	somatic
8	5599399	5605087	5688	I_trimester	Del	somatic or paternally inherited*
8	5601007	5605087	4080	LGA	Del	maternally inherited
8	5601007	5604495	3488	GD	Del	paternally inherited
8	6178302	6204395	26093	LGA	Del	somatic
8	8583108	8587589	4481	GD	Del	maternally inherited
8	13390938	13531381	140443	GD	Dupl	paternally inherited
8	15015155	15095084	79929	normal term	Dupl	somatic
8	15402936	15410250	7314	I_trimester	Del	maternally inherited
8	15949771	16021467	71696	normal term	Del	paternally inherited
8	16925693	16978400	52707	SGA	Dupl	somatic
8	16925693	16978400	52707	GD	Dupl	somatic
8	17580791	17581699	908	normal term	Del	maternally inherited
8	17580791	17581699	908	LGA	Del	maternally/paternally inherited
8	17580791	17581699	908	normal term	Del	somatic
8	18852474	18858239	5765	normal term	Del	somatic
8	30535659	30581500	45841	II_trimester	Del	somatic or paternally inherited*
8	38190769	38240007	49238	I_trimester	Dupl	parental data not available
8	51031221	51033517	2296	I_trimester	Del	parental data not available
8	51031221	51033517	2296	LGA	Del	somatic
8	51031221	51033517	2296	LGA	Del	somatic
8	51031221	51033517	2296	GD	Del	somatic
8	51125276	52619866	1494590	I_trimester	Dupl	somatic or paternally inherited*
8	55688171	55865884	177713	PE	Dupl	somatic
8	55693447	55828934	135487	LGA	Dupl	somatic
8	60486037	60565984	79947	SGA	Dupl	somatic
8	66285716	66597341	311625	LGA	Del	somatic
8	66396331	66597341	201010	normal term	Del	somatic
8	67465588	67934729	469141	normal term	Del	somatic

Chr	Start	End	Length	Group	Type	Inherited/somatic
8	67590460	67677587	87127	LGA	Del	somatic
8	67594777	67677587	82810	LGA	Del	somatic
8	67623174	67677587	54413	normal term	Del	somatic
8	69444341	69516939	72598	LGA	Dupl	somatic
8	72215549	72217689	2140	GD	Del	somatic
8	72215549	72217689	2140	II_trimester	Del	somatic or paternally inherited*
8	72215549	72217689	2140	II_trimester	Del	somatic or paternally inherited*
8	73169918	73172443	2525	GD	Del	somatic
8	73169918	73172443	2525	II_trimester	Del	somatic or paternally inherited*
8	75925763	76037567	111804	normal term	Dupl	somatic
8	78141371	78637869	496498	normal term	Dupl	somatic
8	78170272	78551701	381429	SGA	Dupl	somatic
8	78214641	78633267	418626	I_trimester	Dupl	somatic or paternally inherited*
8	78352373	78637869	285496	II_trimester	Dupl	parental data not available
8	81101996	81389791	287795	PE	Del	somatic
8	81149186	81358791	209605	normal term	Del	somatic
8	83271395	83293872	22477	PE	Del	maternally inherited
8	84077412	84097208	19796	normal term	Dupl	somatic
8	84077412	84097208	19796	PE	Dupl	somatic
8	84077412	84097208	19796	GD	Dupl	somatic
8	85257540	85265997	8457	PE	Del	somatic
8	89119304	89157918	38614	II_trimester	Dupl	somatic or paternally inherited*
8	92107070	92183658	76588	normal term	Del	maternally inherited
8	92128840	92183658	54818	SGA	Del	maternally inherited
8	92391870	92479701	87831	normal term	Dupl	somatic
8	95809165	95820969	11804	II_trimester	Del	somatic or paternally inherited*
8	105957540	105981846	24306	LGA	Dupl	somatic
8	113219539	114227020	1007481	II_trimester	Dupl	parental data not available
8	113234556	114227020	992464	II_trimester	Dupl	somatic or paternally inherited*
8	113235742	114227020	991278	PE	Dupl	somatic
8	113411888	114194727	782839	normal term	Dupl	somatic
8	113411888	114199799	787911	LGA	Dupl	somatic
8	113411888	114227020	815132	GD	Dupl	somatic
8	113903821	114227020	323199	II_trimester	Dupl	somatic or paternally inherited*
8	113960534	114196344	235810	LGA	Dupl	somatic
8	115641238	115642408	1170	normal term	Del	somatic
8	115641238	115642408	1170	II_trimester	Del	somatic or paternally inherited*
8	115641238	115642408	1170	II_trimester	Del	somatic or paternally inherited*
8	115641238	115642408	1170	I_trimester	Del	somatic or paternally inherited*
8	116537833	117099512	561679	I_trimester	Dupl	somatic or paternally inherited*
8	127150370	127157289	6919	normal term	Del	somatic
8	135771473	135847442	75969	II_trimester	Dupl	parental data not available
8	136539132	136667194	128062	PE	Dupl	somatic
8	137682484	137857327	174843	GD	Del	paternally inherited
8	137697834	137857327	159493	normal term	Del	maternally inherited
8	144460569	144687092	226523	normal term	Dupl	somatic
8	144936032	145018354	82322	I_trimester	Dupl	somatic or paternally inherited*

Chr	Start	End	Length	Group	Type	Inherited/somatic
8	144974963	145067467	92504	I_trimester	Dupl	parental data not available
8	144974963	145059425	84462	I_trimester	Dupl	somatic or paternally inherited*
8	144976654	145067467	90813	normal term	Dupl	somatic
8	144976654	145059425	82771	SGA	Dupl	somatic
8	144976654	145018354	41700	LGA	Dupl	somatic
9	395025	707463	312438	II_trimester	Dupl	maternally inherited
9	2148213	2151194	2981	II_trimester	Del	somatic or paternally inherited*
9	2148213	2151194	2981	I_trimester	Del	somatic or paternally inherited*
9	5306824	5335470	28646	GD	Del	paternally inherited
9	6701130	6705824	4694	normal term	Del	somatic
9	6701130	6705824	4694	LGA	Del	somatic
9	9292356	9338113	45757	normal term	Dupl	somatic
9	10354430	10369161	14731	SGA	Dupl	somatic
9	10543899	10649660	105761	II_trimester	Dupl	parental data not available
9	10595208	10649660	54452	II_trimester	Dupl	somatic or paternally inherited*
9	10595208	10649660	54452	II_trimester	Dupl	somatic or paternally inherited*
9	11127388	11434678	307290	normal term	Dupl	somatic
9	11274798	11434678	159880	normal term	Dupl	somatic
9	11434678	11463799	29121	PE	Del	paternally inherited
9	12112566	12216756	104190	normal term	Dupl	somatic
9	12578438	12716172	137734	normal term	Dupl	somatic
9	14585052	14679206	94154	GD	Dupl	somatic
9	14589694	14679206	89512	normal term	Dupl	somatic
9	14589694	14692676	102982	normal term	Dupl	somatic
9	14590805	14677538	86733	normal term	Dupl	somatic
9	14590805	14677538	86733	II_trimester	Dupl	somatic or paternally inherited*
9	17400701	17503073	102372	normal term	Dupl	somatic
9	17400701	17502205	101504	I_trimester	Dupl	somatic or paternally inherited*
9	17457854	17502205	44351	II_trimester	Dupl	parental data not available
9	19010867	19133732	122865	LGA	Del	somatic
9	19030014	19303086	273072	PE	Del	somatic
9	19045502	19419528	374026	LGA	Del	somatic
9	19132051	19253268	121217	normal term	Del	somatic
9	19138990	19211852	72862	PE	Del	somatic
9	19138990	19206930	67940	PE	Del	somatic
9	19138990	19223304	84314	GD	Del	somatic
9	19142014	19206930	64916	GD	Del	somatic
9	19142014	19206930	64916	GD	Del	somatic
9	19142014	19253268	111254	SGA	Del	somatic or paternally inherited*
9	19142014	19258183	116169	II_trimester	Del	somatic or paternally inherited*
9	24573717	24616032	42315	II_trimester	Dupl	somatic or paternally inherited*
9	24754015	24795767	41752	SGA	Del	paternally inherited
9	24922485	24999327	76842	II_trimester	Dupl	somatic or paternally inherited*
9	26567999	26572537	4538	PE	Del	somatic
9	29584528	29606866	22338	LGA	Dupl	somatic
9	33818257	34237480	419223	normal term	Del	somatic
9	73907625	73909871	2246	normal term	Dupl	paternally inherited

Chr	Start	End	Length	Group	Type	Inherited/somatic
9	75519946	75523871	3925	SGA	Dupl	somatic
9	86488586	86571798	83212	LGA	Del	somatic
9	127764305	128099037	334732	PE	Del	somatic
9	127784990	128116817	331827	PE	Del	somatic
9	132649662	132830460	180798	LGA	Del	somatic
9	132655864	132849126	193262	normal term	Del	somatic
9	132655864	132849126	193262	LGA	Del	somatic
9	139220876	139303300	82424	LGA	Dupl	somatic
9	139240630	139303300	62670	PE	Dupl	somatic
9	139240630	139303300	62670	PE	Dupl	somatic
9	139240630	139303300	62670	II_trimester	Dupl	somatic or paternally inherited*
9	139240630	139303300	62670	II_trimester	Dupl	somatic or paternally inherited*
9	139240630	139306467	65837	II_trimester	Dupl	somatic or paternally inherited*
10	4627791	4672037	44246	SGA	Dupl	somatic
10	12292343	12369860	77517	LGA	Del	somatic
10	13056587	13058458	1871	SGA	Del	maternally inherited
10	15070615	15117114	46499	LGA	Del	somatic
10	15071618	15117114	45496	normal term	Del	somatic
10	20842031	20857365	15334	SGA	Del	somatic
10	20850624	20857365	6741	LGA	Del	maternally inherited
10	20850624	20857365	6741	normal term	Del	somatic
10	20850624	20857365	6741	SGA	Del	somatic
10	20850624	20857365	6741	PE	Del	somatic
10	20850624	20857365	6741	PE	Del	somatic
10	20850624	20857365	6741	GD	Del	somatic
10	20850624	20857365	6741	SGA	Del	somatic or paternally inherited*
10	20850624	20857365	6741	II_trimester	Del	somatic or paternally inherited*
10	20850624	20857365	6741	II_trimester	Del	somatic or paternally inherited*
10	20850624	20857365	6741	II_trimester	Del	somatic or paternally inherited*
10	20850624	20857365	6741	II_trimester	Del	somatic or paternally inherited*
10	20850624	20857365	6741	II_trimester	Del	somatic or paternally inherited*
10	20850624	20857365	6741	I_trimester	Del	somatic or paternally inherited*
10	21701220	21740640	39420	LGA	Del	somatic
10	22209221	22336151	126930	II_trimester	Dupl	somatic or paternally inherited*
10	27075698	27266501	190803	LGA	Del	somatic
10	37232329	37671202	438873	I_trimester	Dupl	somatic or paternally inherited*
10	37236853	37274522	37669	LGA	Dupl	somatic
10	37239564	37283349	43785	SGA	Dupl	somatic
10	45118949	45446917	327968	SGA	Dupl	maternally inherited
10	47543322	47568296	24974	I_trimester	Dupl	maternally inherited
10	47543322	47703869	160547	II_trimester	Dupl	somatic or paternally inherited*
10	53385459	53404012	18553	II_trimester	Del	somatic or paternally inherited*
10	53657647	53682130	24483	II_trimester	Del	somatic or paternally inherited*
10	54016061	54016781	720	SGA	Del	somatic
10	54016061	54016781	720	SGA	Del	somatic
10	54016061	54016781	720	I_trimester	Del	somatic or paternally inherited*
10	55735462	55779914	44452	SGA	Del	maternally inherited

Chr	Start	End	Length	Group	Type	Inherited/somatic
10	56971092	57012515	41423	normal term	Dupl	somatic
10	59210800	59211698	898	PE	Del	somatic
10	59210800	59211698	898	PE	Del	somatic
10	60839167	61035570	196403	normal term	Dupl	somatic
10	62212334	62320330	107996	II_trimester	Dupl	somatic or paternally inherited*
10	62235443	62320330	84887	GD	Dupl	somatic
10	62259224	62320330	61106	I_trimester	Dupl	somatic or paternally inherited*
10	63116273	63204366	88093	PE	Dupl	somatic
10	63149922	63187103	37181	II_trimester	Dupl	somatic or paternally inherited*
10	63157465	63187103	29638	II_trimester	Dupl	parental data not available
10	63157465	63187103	29638	I_trimester	Dupl	parental data not available
10	63157465	63204366	46901	normal term	Dupl	somatic
10	63157465	63204366	46901	normal term	Dupl	somatic
10	63157465	63183583	26118	normal term	Dupl	somatic
10	63157465	63187103	29638	SGA	Dupl	somatic
10	63157465	63183583	26118	GD	Dupl	somatic
10	63157465	63187103	29638	GD	Dupl	somatic
10	63157465	63204366	46901	GD	Dupl	somatic
10	63157465	63183583	26118	GD	Dupl	somatic
10	63157465	63187103	29638	II_trimester	Dupl	somatic or paternally inherited*
10	63157465	63187103	29638	II_trimester	Dupl	somatic or paternally inherited*
10	63157465	63204366	46901	I_trimester	Dupl	somatic or paternally inherited*
10	63166773	63221167	54394	normal term	Dupl	somatic
10	64819996	64919511	99515	II_trimester	Del	somatic or paternally inherited*
10	66884125	66904588	20463	GD	Dupl	somatic
10	66885915	66909326	23411	II_trimester	Dupl	parental data not available
10	66885915	66909326	23411	normal term	Dupl	somatic
10	66885915	66913425	27510	SGA	Dupl	somatic
10	66885915	66909326	23411	GD	Dupl	somatic
10	68065751	68102333	36582	SGA	Del	maternally inherited
10	68072084	68114481	42397	I_trimester	Del	maternally inherited
10	68078481	68114481	36000	LGA	Del	maternally inherited
10	68078481	68114481	36000	II_trimester	Del	maternally inherited
10	68845232	68898306	53074	II_trimester	Dupl	parental data not available
10	68845232	68888491	43259	normal term	Dupl	somatic
10	68845232	68898306	53074	SGA	Dupl	somatic
10	68845232	68888491	43259	PE	Dupl	somatic
10	68845232	68888491	43259	GD	Dupl	somatic
10	68845232	68898306	53074	GD	Dupl	somatic
10	68845232	68898306	53074	II_trimester	Dupl	somatic or paternally inherited*
10	68845232	68898306	53074	II_trimester	Dupl	somatic or paternally inherited*
10	68845232	68898306	53074	II_trimester	Dupl	somatic or paternally inherited*
10	68845232	68898306	53074	I_trimester	Dupl	somatic or paternally inherited*
10	70061336	70613280	551944	LGA	Del	somatic
10	70127696	70319626	191930	normal term	Del	somatic
10	70127696	70595425	467729	II_trimester	Del	somatic or paternally inherited*
10	70174706	70616973	442267	normal term	Del	somatic

Chr	Start	End	Length	Group	Type	Inherited/somatic
10	71641240	71652128	10888	LGA	Dupl	somatic
10	74317852	74442258	124406	II_trimester	Del	somatic or paternally inherited*
10	83944705	83959178	14473	PE	Del	maternally inherited
10	88768657	88799610	30953	II_trimester	Del	somatic or paternally inherited*
10	88786593	88813420	26827	LGA	Del	somatic
10	95426888	95459817	32929	I_trimester	Dupl	somatic or paternally inherited*
10	96499710	96564326	64616	I_trimester	Del	maternally inherited
10	96557335	96594311	36976	II_trimester	Del	somatic or paternally inherited*
10	98410170	98684650	274480	LGA	Del	somatic
10	98410901	98657516	246615	LGA	Del	somatic
10	98508321	98684650	176329	PE	Del	somatic
10	98513814	98684650	170836	PE	Del	somatic
10	98513814	98604466	90652	normal term	Del	somatic
10	98513814	98684650	170836	LGA	Del	somatic
10	98513814	98657516	143702	II_trimester	Del	somatic or paternally inherited*
10	98513814	98657516	143702	I_trimester	Del	somatic or paternally inherited*
10	98521516	98684650	163134	II_trimester	Del	somatic or paternally inherited*
10	99003375	99059645	56270	LGA	Del	somatic
10	102111045	102209160	98115	II_trimester	Del	somatic or paternally inherited*
10	102152297	102209160	56863	LGA	Del	somatic
10	102152297	102209160	56863	LGA	Del	somatic
10	115660297	115680935	20638	GD	Dupl	somatic
10	119714635	119813953	99318	normal term	Dupl	somatic
10	120850164	120880910	30746	normal term	Del	somatic
10	123642539	123801422	158883	LGA	Del	somatic
10	124178977	124220681	41704	LGA	Dupl	somatic or paternally inherited*
10	130458098	130835311	377213	normal term	Dupl	somatic
10	133943800	133966434	22634	II_trimester	Dupl	somatic or paternally inherited*
10	133946326	134102055	155729	LGA	Dupl	somatic
10	134529972	134693532	163560	normal term	Dupl	paternally inherited
10	135234393	135378802	144409	I_trimester	Dupl	parental data not available
10	135234393	135508269	273876	I_trimester	Dupl	somatic or paternally inherited*
10	135252347	135377448	125101	normal term	Dupl	maternally inherited
10	135252347	135508269	255922	PE	Dupl	maternally inherited
10	135252347	135508269	255922	II_trimester	Dupl	maternally inherited
10	135252347	135378802	126455	I_trimester	Dupl	maternally inherited
11	382912	424290	41378	normal term	Dupl	somatic
11	2612560	2743858	131298	normal term	Dupl	somatic
11	3242222	3243426	1204	GD	Del	somatic
11	3698842	3837204	138362	LGA	Del	somatic
11	3849710	4168342	318632	normal term	Del	somatic
11	6340705	6355016	14311	normal term	Del	somatic
11	9215691	9516186	300495	PE	Del	somatic
11	9226514	9516186	289672	PE	Del	somatic
11	9226514	9594827	368313	PE	Del	somatic
11	9258146	9593426	335280	SGA	Del	somatic or paternally inherited*
11	17111027	17394596	283569	LGA	Del	somatic

Chr	Start	End	Length	Group	Type	Inherited/somatic
11	17111027	17308130	197103	PE	Del	somatic
11	18949220	18956690	7470	PE	Del	somatic
11	22391419	22400813	9394	SGA	Dupl	somatic
11	24412620	24551109	138489	I_trimester	Dupl	somatic or paternally inherited*
11	24419480	24551109	131629	II_trimester	Dupl	somatic or paternally inherited*
11	31595748	31729792	134044	I_trimester	Dupl	parental data not available
11	34458229	34460861	2632	SGA	Dupl	somatic
11	37080974	37150239	69265	LGA	Dupl	somatic
11	37083616	37150239	66623	PE	Dupl	somatic
11	48647107	49146066	498959	PE	Del	somatic
11	48650491	48942781	292290	PE	Del	somatic
11	48650491	48942781	292290	LGA	Del	somatic
11	48650491	48942781	292290	LGA	Del	somatic
11	48650491	48942781	292290	PE	Del	somatic
11	48650491	48987538	337047	PE	Del	somatic
11	48651903	48942781	290878	II_trimester	Del	parental data not available
11	48651903	48987538	335635	normal term	Del	somatic
11	48651903	49010832	358929	normal term	Del	somatic
11	48651903	48942781	290878	normal term	Del	somatic
11	48651903	48942781	290878	normal term	Del	somatic
11	48651903	48942781	290878	normal term	Del	somatic
11	48651903	48942781	290878	LGA	Del	somatic
11	48651903	48942781	290878	PE	Del	somatic
11	48651903	49010832	358929	GD	Del	somatic
11	48651903	48942781	290878	II_trimester	Del	somatic or paternally inherited*
11	48651903	48942781	290878	I_trimester	Del	somatic or paternally inherited*
11	48651903	48942781	290878	I_trimester	Del	somatic or paternally inherited*
11	48651903	48942781	290878	I_trimester	Del	somatic or paternally inherited*
11	48694871	48942781	247910	normal term	Del	somatic
11	48694871	49010832	315961	SGA	Del	somatic
11	48694871	48942781	247910	SGA	Del	somatic
11	48694871	48942781	247910	II_trimester	Del	somatic or paternally inherited*
11	48694871	48942781	247910	II_trimester	Del	somatic or paternally inherited*
11	48694871	48942781	247910	I_trimester	Del	somatic or paternally inherited*
11	48747611	48942781	195170	normal term	Del	somatic
11	48747611	48942781	195170	PE	Del	somatic
11	48747611	48942781	195170	II_trimester	Del	somatic or paternally inherited*
11	48747611	48942781	195170	I_trimester	Del	somatic or paternally inherited*
11	48754534	48942781	188247	LGA	Del	somatic
11	48760364	48942781	182417	GD	Del	somatic
11	48864169	48942781	78612	I_trimester	Del	somatic or paternally inherited*
11	48987538	50057854	1070316	LGA	Dupl	somatic
11	55360213	55465912	105699	II_trimester	Del	somatic or paternally inherited*
11	55365761	55427700	61939	normal term	Del	somatic
11	55365761	55451045	85284	normal term	Del	somatic
11	55365761	55451045	85284	PE	Del	somatic
11	55365761	55427700	61939	GD	Del	somatic

Chr	Start	End	Length	Group	Type	Inherited/somatic
11	55431897	55451045	19148	GD	Del	somatic
11	59204920	59241747	36827	PE	Del	paternally inherited
11	63341096	63723238	382142	PE	Del	somatic
11	63343322	63579191	235869	LGA	Del	somatic
11	63550922	63570347	19425	II_trimester	Del	somatic or paternally inherited*
11	66615838	66626234	10396	SGA	Dupl	somatic
11	66832528	67011765	179237	normal term	Del	somatic
11	70077881	70118489	40608	II_trimester	Del	somatic or paternally inherited*
11	73109635	73576748	467113	normal term	Del	somatic
11	73378142	73581908	203766	PE	Del	somatic
11	73383382	73576748	193366	II_trimester	Del	somatic or paternally inherited*
11	81503992	81517261	13269	GD	Del	maternally inherited
11	81503992	81517261	13269	II_trimester	Del	somatic or paternally inherited*
11	81556771	81813575	256804	normal term	Dupl	somatic
11	86931924	87033619	101695	SGA	Dupl	somatic
11	88696662	88794180	97518	SGA	Dupl	somatic
11	88696662	88794180	97518	SGA	Dupl	somatic
11	88696662	88794180	97518	LGA	Dupl	somatic
11	88696662	88744425	47763	PE	Dupl	somatic
11	88696662	88794180	97518	PE	Dupl	somatic
11	88696662	88794180	97518	GD	Dupl	somatic
11	88696662	88753844	57182	GD	Dupl	somatic
11	88696662	88744425	47763	SGA	Dupl	somatic or paternally inherited*
11	88696662	88794180	97518	LGA	Dupl	somatic or paternally inherited*
11	88696662	88794180	97518	II_trimester	Dupl	somatic or paternally inherited*
11	88696662	88794180	97518	II_trimester	Dupl	somatic or paternally inherited*
11	88696662	88794180	97518	II_trimester	Dupl	somatic or paternally inherited*
11	88696662	88794180	97518	II_trimester	Dupl	somatic or paternally inherited*
11	88696662	88747548	50886	I_trimester	Dupl	somatic or paternally inherited*
11	88696662	88794180	97518	I_trimester	Dupl	somatic or paternally inherited*
11	90975749	91024468	48719	II_trimester	Dupl	parental data not available
11	90975749	91019763	44014	II_trimester	Dupl	somatic or paternally inherited*
11	97861301	98621717	760416	PE	Del	paternally inherited
11	99377407	99588130	210723	SGA	Dupl	paternally inherited
11	99525126	99566782	41656	GD	Del	maternally inherited
11	101319531	101470545	151014	II_trimester	Dupl	somatic or paternally inherited*
11	107617517	107821944	204427	LGA	Del	somatic
11	107663102	107772104	109002	LGA	Del	somatic
11	109111782	109156782	45000	GD	Del	paternally inherited
11	120557343	120630452	73109	SGA	Del	maternally inherited
11	120558084	120630452	72368	GD	Del	maternally inherited
11	122700789	122749214	48425	GD	Del	maternally inherited
11	122936483	123017181	80698	LGA	Del	somatic
11	131626702	131645435	18733	PE	Del	somatic
11	131645435	131658127	12692	SGA	Dupl	somatic
11	131645435	131665483	20048	II_trimester	Dupl	somatic or paternally inherited*
11	131651470	131660373	8903	II_trimester	Dupl	somatic or paternally inherited*

Chr	Start	End	Length	Group	Type	Inherited/somatic
11	134603256	134604874	1618	SGA	Del	somatic
11	134603256	134604874	1618	PE	Del	somatic
12	1028314	1133150	104836	normal term	Del	somatic
12	2245636	2252924	7288	II_trimester	Del	maternally inherited
12	6243616	6259552	15936	LGA	Del	somatic
12	6243616	6244157	541	LGA	Del	somatic
12	8003757	8125745	121988	II_trimester	Del	somatic or paternally inherited*
12	10262275	10410360	148085	LGA	Del	somatic
12	12423092	12483764	60672	GD	Del	somatic
12	16250913	16480949	230036	GD	Del	maternally inherited
12	20524202	20551037	26835	I_trimester	Dupl	somatic or paternally inherited*
12	20961322	21032242	70920	SGA	Dupl	somatic
12	20996286	21014178	17892	GD	Dupl	somatic
12	21482399	21498093	15694	II_trimester	Dupl	parental data not available
12	21482399	21498093	15694	normal term	Dupl	somatic
12	21482399	21498093	15694	GD	Dupl	somatic
12	21482399	21498093	15694	II_trimester	Dupl	somatic or paternally inherited*
12	21482399	21498093	15694	II_trimester	Dupl	somatic or paternally inherited*
12	21484512	21497891	13379	normal term	Dupl	somatic
12	21555582	21571978	16396	II_trimester	Dupl	somatic or paternally inherited*
12	22607819	22682681	74862	II_trimester	Dupl	parental data not available
12	22607819	22682681	74862	normal term	Dupl	somatic
12	22607819	22682681	74862	normal term	Dupl	somatic
12	22607819	22666531	58712	SGA	Dupl	somatic
12	22607819	22666531	58712	SGA	Dupl	somatic
12	22607819	22682681	74862	SGA	Dupl	somatic
12	22607819	22682681	74862	LGA	Dupl	somatic
12	22607819	22647856	40037	GD	Dupl	somatic
12	22607819	22682681	74862	II_trimester	Dupl	somatic or paternally inherited*
12	22607819	22682681	74862	II_trimester	Dupl	somatic or paternally inherited*
12	22607819	22682681	74862	I_trimester	Dupl	somatic or paternally inherited*
12	22607819	22682681	74862	I_trimester	Dupl	somatic or paternally inherited*
12	22618605	22682681	64076	PE	Dupl	somatic
12	22618605	22682681	64076	normal term	Dupl	somatic
12	22618605	22682681	64076	normal term	Dupl	somatic
12	22618605	22682681	64076	GD	Dupl	somatic
12	22618605	22682681	64076	II_trimester	Dupl	somatic or paternally inherited*
12	22618605	22682681	64076	I_trimester	Dupl	somatic or paternally inherited*
12	24964383	24970708	6325	LGA	Dupl	somatic
12	24964383	24970708	6325	LGA	Dupl	somatic
12	27648411	27654546	6135	I_trimester	Del	somatic or paternally inherited*
12	31125834	31407303	281469	normal term	Dupl	paternally inherited
12	31266287	31409579	143292	PE	Dupl	maternally inherited
12	31266287	31409579	143292	LGA	Dupl	maternally inherited
12	31266287	31409579	143292	PE	Dupl	maternally inherited
12	31266287	31407303	141016	GD	Dupl	paternally inherited
12	31266287	31408696	142409	normal term	Dupl	somatic

Chr	Start	End	Length	Group	Type	Inherited/somatic
12	31266287	31409778	143491	PE	Dupl	somatic
12	31266287	31409579	143292	LGA	Dupl	somatic or paternally inherited*
12	39124891	39168186	43295	I_trimester	Dupl	somatic or paternally inherited*
12	39967134	40046749	79615	II_trimester	Dupl	parental data not available
12	39967134	40053423	86289	I_trimester	Dupl	somatic or paternally inherited*
12	40875351	40875963	612	SGA	Del	somatic
12	44835241	44936388	101147	normal term	Dupl	somatic
12	52846534	52859985	13451	I_trimester	Del	maternally inherited
12	54319727	54428011	108284	normal term	Dupl	somatic
12	55150448	55214145	63697	normal term	Del	somatic
12	56271676	56339545	67869	normal term	Del	somatic
12	56761845	56777105	15260	LGA	Del	somatic
12	59935926	59942122	6196	normal term	Del	somatic
12	59935926	59942122	6196	LGA	Del	somatic
12	59935926	59942122	6196	PE	Del	somatic
12	59935926	59942122	6196	PE	Del	somatic
12	59935926	59942122	6196	II_trimester	Del	somatic or paternally inherited*
12	59935926	59942122	6196	II_trimester	Del	somatic or paternally inherited*
12	59935926	59942122	6196	II_trimester	Del	somatic or paternally inherited*
12	61300873	61321575	20702	PE	Dupl	somatic
12	70874726	70877257	2531	SGA	Del	somatic
12	72276649	72331317	54668	normal term	Dupl	somatic
12	76421253	76452090	30837	II_trimester	Dupl	somatic or paternally inherited*
12	76439477	76452090	12613	SGA	Dupl	somatic
12	79432940	79484133	51193	normal term	Dupl	somatic
12	79441370	79473658	32288	normal term	Dupl	somatic
12	81007138	81092260	85122	PE	Dupl	somatic
12	81007138	81117172	110034	LGA	Dupl	somatic
12	81007138	81117172	110034	GD	Dupl	somatic
12	81007138	81103143	96005	GD	Dupl	somatic
12	81007138	81092260	85122	GD	Dupl	somatic
12	81007138	81113829	106691	SGA	Dupl	somatic or paternally inherited*
12	81007138	81113829	106691	II_trimester	Dupl	somatic or paternally inherited*
12	81007138	81092260	85122	II_trimester	Dupl	somatic or paternally inherited*
12	81007138	81092260	85122	II_trimester	Dupl	somatic or paternally inherited*
12	81009228	81091063	81835	LGA	Dupl	somatic
12	81009228	81117172	107944	PE	Dupl	somatic
12	81009228	81092260	83032	II_trimester	Dupl	somatic or paternally inherited*
12	82068777	82147486	78709	II_trimester	Dupl	parental data not available
12	91285124	91519855	234731	LGA	Dupl	somatic
12	91366648	91451940	85292	II_trimester	Dupl	somatic or paternally inherited*
12	91495837	91519855	24018	normal term	Dupl	somatic
12	91495837	91519855	24018	PE	Dupl	somatic
12	91495837	91519855	24018	PE	Dupl	somatic
12	91495837	91519855	24018	GD	Dupl	somatic
12	91495837	91509148	13311	LGA	Dupl	somatic or paternally inherited*
12	91495837	91519855	24018	II_trimester	Dupl	somatic or paternally inherited*

Chr	Start	End	Length	Group	Type	Inherited/somatic
12	93265078	93384142	119064	LGA	Del	somatic
12	93279438	93352785	73347	II_trimester	Del	somatic or paternally inherited*
12	101797976	101962012	164036	II_trimester	Del	somatic or paternally inherited*
12	101820622	101949222	128600	II_trimester	Del	somatic or paternally inherited*
12	101820622	101962012	141390	II_trimester	Del	somatic or paternally inherited*
12	102214030	102312229	98199	II_trimester	Del	somatic or paternally inherited*
12	124786088	124987620	201532	normal term	Dupl	somatic
12	124786088	124986454	200366	II_trimester	Dupl	somatic or paternally inherited*
12	124790517	124987620	197103	LGA	Dupl	somatic
12	131494029	131907138	413109	SGA	Dupl	somatic or paternally inherited*
13	20309714	20657188	347474	normal term	Del	somatic
13	20309857	20596565	286708	LGA	Del	somatic
13	21118720	21131210	12490	normal term	Del	somatic
13	22720397	22766634	46237	normal term	Dupl	somatic
13	23349733	23515553	165820	LGA	Dupl	paternally inherited
13	34140340	34143544	3204	GD	Del	somatic
13	35125130	35718203	593073	LGA	Del	somatic
13	45811273	45864198	52925	GD	Dupl	paternally inherited
13	58593657	58599031	5374	PE	Del	paternally inherited
13	58593657	58599031	5374	PE	Del	somatic
13	58593657	58599031	5374	II_trimester	Del	somatic or paternally inherited*
13	63982375	64005461	23086	LGA	Dupl	somatic
13	67770568	67799329	28761	II_trimester	Dupl	somatic or paternally inherited*
13	69252995	69267981	14986	normal term	Del	maternally inherited
13	69276739	69456954	180215	SGA	Dupl	somatic
13	70711212	70794045	82833	normal term	Dupl	somatic
13	70711212	70794045	82833	SGA	Dupl	somatic
13	70712321	70794045	81724	GD	Dupl	somatic
13	70723292	70763597	40305	normal term	Dupl	somatic
13	70744882	70773398	28516	I_trimester	Del	maternally inherited
13	71275838	71353149	77311	II_trimester	Dupl	somatic or paternally inherited*
13	71296379	71353149	56770	normal term	Dupl	somatic
13	83187066	83228160	41094	SGA	Dupl	somatic
13	84103707	84157926	54219	I_trimester	Del	maternally inherited
13	84111773	84157927	46154	I_trimester	Del	maternally inherited
13	85526856	85604405	77549	SGA	Dupl	somatic
13	90355058	90490883	135825	II_trimester	Dupl	maternally inherited
13	90707864	90735758	27894	II_trimester	Dupl	somatic or paternally inherited*
13	99753285	99840875	87590	LGA	Del	somatic
13	100615239	100648099	32860	LGA	Dupl	somatic
13	100666633	100776268	109635	LGA	Del	somatic
13	102472119	102538302	66183	normal term	Dupl	somatic
14	19327823	20676314	1348491	PE	Dupl	somatic
14	20213937	20420338	206401	II_trimester	Dupl	somatic or paternally inherited*
14	20534909	20549093	14184	normal term	Del	somatic
14	25278154	25333115	54961	normal term	Dupl	somatic
14	29762149	30024303	262154	I_trimester	Dupl	somatic or paternally inherited*

Chr	Start	End	Length	Group	Type	Inherited/somatic
14	34953379	34996507	43128	II_trimester	Del	parental data not available
14	35766029	35795544	29515	LGA	Del	somatic
14	40162494	40491964	329470	LGA	Del	somatic
14	40364644	40461852	97208	I_trimester	Del	somatic or paternally inherited*
14	41476279	41667839	191560	LGA	Del	somatic
14	44507151	44613782	106631	normal term	Dupl	somatic
14	47181164	47396215	215051	II_trimester	Dupl	parental data not available
14	47181164	47408344	227180	SGA	Dupl	somatic
14	47181164	47408344	227180	II_trimester	Dupl	somatic or paternally inherited*
14	47181164	47409115	227951	II_trimester	Dupl	somatic or paternally inherited*
14	47188267	47408344	220077	normal term	Dupl	somatic
14	47249450	47409115	159665	normal term	Dupl	somatic
14	47310983	47408344	97361	normal term	Dupl	somatic
14	47310983	47409115	98132	PE	Dupl	somatic
14	47320923	47409115	88192	normal term	Dupl	somatic
14	47320923	47409115	88192	LGA	Dupl	somatic
14	50109177	50199883	90706	normal term	Del	somatic
14	54425241	54527202	101961	LGA	Del	somatic
14	56103882	56173687	69805	II_trimester	Dupl	parental data not available
14	56103882	56166871	62989	normal term	Dupl	somatic
14	56103882	56150151	46269	normal term	Dupl	somatic
14	58860893	58921398	60505	II_trimester	Del	somatic or paternally inherited*
14	62557934	62613179	55245	I_trimester	Dupl	somatic or paternally inherited*
14	62650341	62734866	84525	SGA	Del	maternally inherited
14	63926572	64474188	547616	normal term	Del	somatic
14	84142036	84223764	81728	normal term	Dupl	somatic
14	84142036	84223764	81728	PE	Dupl	somatic
14	84142036	84223764	81728	II_trimester	Dupl	somatic or paternally inherited*
14	84159255	84223764	64509	normal term	Dupl	somatic
14	84159255	84223764	64509	II_trimester	Dupl	somatic or paternally inherited*
14	86451688	86481388	29700	I_trimester	Del	somatic or paternally inherited*
14	86458414	86481388	22974	GD	Del	paternally inherited
14	93395086	93506806	111720	normal term	Dupl	somatic
14	93398849	93609385	210536	II_trimester	Dupl	somatic or paternally inherited*
14	96742603	96858571	115968	II_trimester	Dupl	parental data not available
14	99977347	100064674	87327	LGA	Dupl	somatic
14	105473229	107287663	1814434	normal term	Dupl	somatic
14	105617042	105685865	68823	normal term	Dupl	somatic
14	105617042	107287663	1670621	PE	Dupl	somatic
14	105660766	105685865	25099	LGA	Dupl	somatic
14	105805055	107287663	1482608	LGA	Dupl	somatic
14	105828768	107287663	1458895	II_trimester	Dupl	maternally inherited
14	105970176	107287663	1317487	normal term	Dupl	somatic
14	106047905	107287663	1239758	I_trimester	Dupl	somatic or paternally inherited*
14	106326623	106949307	622684	LGA	Dupl	somatic
14	106326623	106949307	622684	LGA	Dupl	somatic
14	106326623	106949307	622684	II_trimester	Dupl	somatic or paternally inherited*

Chr	Start	End	Length	Group	Type	Inherited/somatic
14	106326623	106949307	622684	I_trimester	Dupl	somatic or paternally inherited*
14	106326623	106949307	622684	I_trimester	Dupl	somatic or paternally inherited*
14	106350394	106949307	598913	II_trimester	Dupl	somatic or paternally inherited*
14	106350394	106949307	598913	II_trimester	Dupl	somatic or paternally inherited*
15	24050258	24061497	11239	I_trimester	Del	somatic or paternally inherited*
15	24594816	24668007	73191	PE	Dupl	paternally inherited
15	24619900	24668007	48107	GD	Dupl	maternally inherited
15	24619900	24714849	94949	normal term	Dupl	somatic
15	25174628	25188893	14265	normal term	Del	somatic
15	25176516	25188893	12377	normal term	Del	somatic
15	25330884	25494107	163223	normal term	Dupl	somatic
15	25339336	25413557	74221	normal term	Dupl	somatic
15	25345694	25420977	75283	II_trimester	Dupl	parental data not available
15	25420977	25430242	9265	I_trimester	Del	maternally inherited
15	25420977	25430242	9265	normal term	Del	somatic
15	30899133	31089526	190393	PE	Del	maternally inherited
15	35764098	35849809	85711	I_trimester	Dupl	somatic or paternally inherited*
15	35787824	35849809	61985	II_trimester	Dupl	somatic or paternally inherited*
15	35787824	35849809	61985	II_trimester	Dupl	somatic or paternally inherited*
15	37437248	37501850	64602	SGA	Del	paternally inherited
15	41288278	41739356	451078	LGA	Del	somatic
15	41444953	41774423	329470	LGA	Del	somatic
15	42684455	42961680	277225	II_trimester	Del	somatic or paternally inherited*
15	42758737	42961680	202943	normal term	Del	somatic
15	42758737	42950297	191560	LGA	Del	somatic
15	48401875	48521916	120041	II_trimester	Dupl	parental data not available
15	48416359	48481665	65306	normal term	Dupl	somatic
15	50806469	51162261	355792	PE	Del	somatic
15	50944589	51162261	217672	normal term	Del	somatic
15	55707699	55809660	101961	LGA	Del	somatic
15	59862200	59926946	64746	II_trimester	Del	somatic or paternally inherited*
15	65739682	65833174	93492	LGA	Del	somatic
15	74968873	75007799	38926	normal term	Del	somatic
15	87209767	87225334	15567	normal term	Dupl	somatic
15	87210674	87225334	14660	normal term	Dupl	somatic
15	87832130	87863715	31585	PE	Del	maternally inherited
15	89459378	89537952	78574	LGA	Del	somatic
15	97815710	97830540	14830	SGA	Del	somatic
15	100389182	100421490	32308	II_trimester	Dupl	parental data not available
15	100389182	100415574	26392	SGA	Dupl	somatic
15	100389182	100415574	26392	LGA	Dupl	somatic
15	100389182	100445766	56584	PE	Dupl	somatic
15	100389182	100421490	32308	GD	Dupl	somatic
15	100389182	100415574	26392	II_trimester	Dupl	somatic or paternally inherited*
15	100389182	100421490	32308	II_trimester	Dupl	somatic or paternally inherited*
15	100389182	100415574	26392	II_trimester	Dupl	somatic or paternally inherited*
15	102366849	102461162	94313	normal term	Dupl	somatic

Chr	Start	End	Length	Group	Type	Inherited/somatic
15	102366849	102461162	94313	LGA	Dupl	somatic
15	102366849	102461162	94313	PE	Dupl	somatic
15	102366849	102461162	94313	II_trimester	Dupl	somatic or paternally inherited*
15	102366849	102461162	94313	I_trimester	Dupl	somatic or paternally inherited*
15	102376761	102461162	84401	I_trimester	Dupl	somatic or paternally inherited*
16	88164	136257	48093	normal term	Dupl	somatic
16	88164	163666	75502	II_trimester	Dupl	somatic or paternally inherited*
16	570623	917766	347143	normal term	Dupl	somatic
16	572044	855716	283672	II_trimester	Dupl	somatic or paternally inherited*
16	575312	848755	273443	LGA	Dupl	somatic
16	1889714	1934183	44469	normal term	Del	somatic
16	1889714	1934183	44469	LGA	Del	somatic
16	10149904	10164598	14694	PE	Del	paternally inherited
16	10315838	10591887	276049	normal term	Del	somatic
16	12679096	12730650	51554	normal term	Del	paternally inherited
16	12686917	12737141	50224	II_trimester	Del	parental data not available
16	14724589	14771788	47199	LGA	Del	somatic
16	14724589	14771788	47199	PE	Del	somatic
16	18920881	19085297	164416	PE	Del	somatic
16	18920881	19173725	252844	II_trimester	Del	somatic or paternally inherited*
16	18929829	19109992	180163	SGA	Del	somatic
16	18982685	18994925	12240	LGA	Del	somatic
16	19950501	19955468	4967	PE	Del	somatic
16	20521796	20532514	10718	normal term	Del	maternally inherited
16	24508117	24728783	220666	II_trimester	Del	somatic or paternally inherited*
16	24588283	24740128	151845	LGA	Del	somatic
16	28484556	28528623	44067	II_trimester	Del	parental data not available
16	28484556	28528623	44067	LGA	Del	somatic
16	30132387	30192561	60174	LGA	Del	somatic
16	30132387	30198151	65764	II_trimester	Del	somatic or paternally inherited*
16	30415599	30518095	102496	LGA	Del	somatic
16	34241109	34766518	525409	PE	Dupl	somatic
16	47276820	47583872	307052	I_trimester	Dupl	parental data not available
16	50089192	50252235	163043	LGA	Del	somatic
16	55843251	55867738	24487	normal term	Dupl	somatic
16	58589187	58821534	232347	normal term	Del	somatic
16	58599527	58864314	264787	LGA	Del	somatic
16	58629468	58741850	112382	LGA	Del	somatic
16	68127278	68175423	48145	LGA	Del	somatic
16	70285901	70464818	178917	LGA	Del	somatic
16	70303579	70471827	168248	normal term	Del	somatic
16	71292172	71319646	27474	SGA	Dupl	somatic
16	71697752	72022941	325189	LGA	Del	somatic
16	71697752	72078906	381154	LGA	Del	somatic
16	72723613	72785303	61690	GD	Del	paternally inherited
16	78077430	78224186	146756	II_trimester	Del	somatic or paternally inherited*
16	88116130	88570383	454253	II_trimester	Dupl	somatic or paternally inherited*

Chr	Start	End	Length	Group	Type	Inherited/somatic
17	3505233	3564068	58835	II_trimester	Del	maternally inherited
17	4179295	4406973	227678	normal term	Del	somatic
17	16032006	16422727	390721	I_trimester	Del	somatic or paternally inherited*
17	16036593	16178778	142185	normal term	Del	somatic
17	16036593	16422727	386134	II_trimester	Del	somatic or paternally inherited*
17	16058110	16263213	205103	LGA	Del	somatic
17	16058110	16187802	129692	LGA	Del	somatic
17	16058110	16355043	296933	SGA	Del	somatic or paternally inherited*
17	16058110	16246016	187906	II_trimester	Del	somatic or paternally inherited*
17	16127206	16246016	118810	II_trimester	Del	somatic or paternally inherited*
17	17155873	17249887	94014	II_trimester	Del	somatic or paternally inherited*
17	18677329	18851012	173683	LGA	Del	somatic
17	18699071	18830123	131052	normal term	Del	somatic
17	22034501	22142172	107671	SGA	Dupl	somatic
17	27081160	27187636	106476	LGA	Del	somatic or paternally inherited*
17	27081160	27187636	106476	II_trimester	Del	somatic or paternally inherited*
17	27084364	27187636	103272	normal term	Del	somatic
17	27084364	27187636	103272	PE	Del	somatic
17	27084364	27255191	170827	PE	Del	somatic
17	27091951	27187636	95685	normal term	Del	somatic
17	27091951	27187636	95685	SGA	Del	somatic
17	27091951	27187636	95685	SGA	Del	somatic or paternally inherited*
17	27123816	27182944	59128	PE	Del	somatic
17	27123816	27194634	70818	normal term	Del	somatic
17	27123816	27187636	63820	LGA	Del	somatic
17	27123816	27187636	63820	LGA	Del	somatic
17	27123816	27187636	63820	II_trimester	Del	somatic or paternally inherited*
17	27611880	27891864	279984	normal term	Del	somatic
17	28967865	29521832	553967	PE	Del	somatic
17	33684035	33769883	85848	I_trimester	Del	somatic or paternally inherited*
17	37403979	37609119	205140	normal term	Del	somatic
17	37436711	37682657	245946	II_trimester	Del	somatic or paternally inherited*
17	38374131	38446564	72433	LGA	Del	somatic
17	38386114	38460374	74260	LGA	Del	somatic
17	44165803	44352131	186328	LGA	Dupl	maternally inherited
17	45583375	45764692	181317	normal term	Del	somatic
17	45583375	45730501	147126	LGA	Del	somatic
17	56585871	57691502	1105631	PE	Del	somatic
17	57783934	58024274	240340	normal term	Del	somatic
17	57783934	58024324	240390	LGA	Del	somatic
17	58218881	58809767	590886	LGA	Del	somatic
17	65762341	65821166	58825	II_trimester	Del	somatic or paternally inherited*
17	74740399	74820856	80457	LGA	Del	somatic
17	74740399	74828784	88385	LGA	Del	somatic
17	74774824	74828784	53960	normal term	Del	somatic
17	77361176	77404093	42917	LGA	Dupl	paternally inherited
17	77361176	77394037	32861	PE	Dupl	somatic

Chr	Start	End	Length	Group	Type	Inherited/somatic
18	9922512	9965338	42826	II_trimester	Dupl	parental data not available
18	12053552	12132247	78695	II_trimester	Dupl	parental data not available
18	12053552	12132247	78695	normal term	Dupl	somatic
18	12053552	12132247	78695	SGA	Dupl	somatic or paternally inherited*
18	12056850	12132247	75397	II_trimester	Dupl	somatic or paternally inherited*
18	12083314	12132247	48933	normal term	Dupl	somatic
18	12083314	12132247	48933	GD	Dupl	somatic
18	12083314	12132247	48933	II_trimester	Dupl	somatic or paternally inherited*
18	12096249	12132247	35998	PE	Dupl	somatic
18	12096249	12132247	35998	II_trimester	Dupl	somatic or paternally inherited*
18	12096249	12132247	35998	II_trimester	Dupl	somatic or paternally inherited*
18	20919574	21080674	161100	normal term	Del	somatic
18	23759474	23840837	81363	II_trimester	Del	somatic or paternally inherited*
18	24454351	24519923	65572	LGA	Dupl	somatic
18	24493117	24498544	5427	normal term	Dupl	somatic
18	24493117	24498689	5572	II_trimester	Dupl	somatic or paternally inherited*
18	24493424	24498427	5003	normal term	Dupl	somatic
18	26140557	26174416	33859	SGA	Dupl	somatic
18	29488804	29687013	198209	PE	Del	somatic
18	29527884	29711260	183376	normal term	Del	somatic
18	29554990	29687013	132023	LGA	Del	somatic
18	38573994	38695982	121988	normal term	Dupl	somatic
18	38573994	38630544	56550	SGA	Dupl	somatic
18	38573994	38632365	58371	GD	Dupl	somatic
18	38573994	38630544	56550	I_trimester	Dupl	somatic or paternally inherited*
18	40365619	40488279	122660	PE	Dupl	somatic
18	40475051	40488279	13228	II_trimester	Dupl	somatic or paternally inherited*
18	40475218	40488279	13061	SGA	Dupl	somatic
18	40945960	41083535	137575	I_trimester	Dupl	somatic or paternally inherited*
18	63729570	63730950	1380	normal term	Del	somatic
18	63729570	63730950	1380	PE	Del	somatic
18	63729570	63730950	1380	LGA	Del	somatic or paternally inherited*
18	64158664	64210748	52084	II_trimester	Dupl	parental data not available
18	64166846	64245402	78556	II_trimester	Dupl	somatic or paternally inherited*
18	64166846	64210748	43902	II_trimester	Dupl	somatic or paternally inherited*
18	64171511	64175267	3756	SGA	Dupl	somatic
18	65252640	65692392	439752	LGA	Dupl	paternally inherited
18	65537177	65608116	70939	SGA	Dupl	somatic
18	66747568	66755508	7940	LGA	Del	paternally inherited
18	66747568	66755508	7940	I_trimester	Del	maternally inherited
18	75389907	75455105	65198	normal term	Dupl	somatic
18	75408509	75455105	46596	normal term	Dupl	somatic
18	75408509	75455105	46596	I_trimester	Dupl	somatic or paternally inherited*
18	77173979	77251061	77082	LGA	Dupl	somatic
18	77914537	77926197	11660	normal term	Dupl	somatic
18	77914537	77931598	17061	GD	Dupl	somatic
19	4718201	4848593	130392	SGA	Del	somatic

Chr	Start	End	Length	Group	Type	Inherited/somatic
19	9119242	9380978	261736	PE	Del	somatic
19	15570931	15645620	74689	II_trimester	Del	somatic or paternally inherited*
19	20621828	20729120	107292	SGA	Del	maternally inherited
19	20621828	20715228	93400	PE	Del	paternally inherited
19	20621828	20715228	93400	SGA	Del	somatic
19	20621828	20715228	93400	II_trimester	Del	somatic or paternally inherited*
19	22121293	22158992	37699	II_trimester	Dupl	somatic or paternally inherited*
19	22143339	22175566	32227	I_trimester	Dupl	somatic or paternally inherited*
19	27763440	28079145	315705	I_trimester	Del	somatic or paternally inherited*
19	33872410	33921542	49132	normal term	Dupl	somatic
19	35661787	35665514	3727	II_trimester	Del	somatic or paternally inherited*
19	35856925	35861695	4770	LGA	Del	somatic
19	35856925	35861695	4770	II_trimester	Del	somatic or paternally inherited*
19	41341588	41384577	42989	normal term	Del	somatic
19	41350894	41383827	32933	PE	Del	paternally inherited
19	41350894	41384577	33683	I_trimester	Del	somatic or paternally inherited*
19	43328006	43866823	538817	normal term	Dupl	somatic
19	43371294	43699208	327914	I_trimester	Del	maternally inherited
19	43372386	43770566	398180	normal term	Del	maternally inherited
19	43372386	43539189	166803	II_trimester	Del	somatic or paternally inherited*
19	43663086	43845593	182507	I_trimester	Del	maternally inherited
19	49084056	49092429	8373	PE	Del	somatic
19	54253212	54303712	50500	I_trimester	Del	somatic or paternally inherited*
20	4906003	5046930	140927	II_trimester	Del	somatic or paternally inherited*
20	5020237	5046930	26693	LGA	Del	somatic
20	7288480	7326701	38221	normal term	Dupl	somatic
20	7288480	7326701	38221	LGA	Dupl	somatic
20	7288480	7326701	38221	PE	Dupl	somatic
20	7288480	7326701	38221	II_trimester	Dupl	somatic or paternally inherited*
20	9069657	9078936	9279	normal term	Dupl	somatic
20	11044604	11061145	16541	normal term	Dupl	somatic
20	12807016	12901895	94879	SGA	Dupl	somatic
20	14568758	14576448	7690	II_trimester	Dupl	somatic or paternally inherited*
20	29804293	29874664	70371	II_trimester	Del	parental data not available
20	29804293	29820178	15885	normal term	Del	somatic
20	29804293	29874664	70371	GD	Del	somatic
20	29804293	29874664	70371	II_trimester	Del	somatic or paternally inherited*
20	29804293	29843182	38889	II_trimester	Del	somatic or paternally inherited*
20	34378692	34501092	122400	LGA	Del	somatic
20	34378692	34499368	120676	II_trimester	Del	somatic or paternally inherited*
20	45726192	46208604	482412	II_trimester	Del	somatic or paternally inherited*
20	45877226	46208604	331378	PE	Del	somatic
20	46000938	46208604	207666	normal term	Del	somatic
20	46000938	46208604	207666	PE	Del	somatic
20	46000938	46186571	185633	II_trimester	Del	somatic or paternally inherited*
20	46000938	46190401	189463	II_trimester	Del	somatic or paternally inherited*
20	52648368	52654547	6179	I_trimester	Del	somatic or paternally inherited*

Chr	Start	End	Length	Group	Type	Inherited/somatic
20	55902423	55940426	38003	normal term	Dupl	somatic
20	55904180	55940426	36246	normal term	Dupl	somatic
20	55904180	55940426	36246	II_trimester	Dupl	somatic or paternally inherited*
20	59113601	59122693	9092	GD	Del	somatic
20	62038276	62053754	15478	normal term	Dupl	somatic
20	62038276	62053754	15478	LGA	Dupl	somatic
20	62038276	62065610	27334	GD	Dupl	somatic
20	62038276	62078487	40211	GD	Dupl	somatic
20	62038276	62053754	15478	II_trimester	Dupl	somatic or paternally inherited*
20	62038276	62053754	15478	II_trimester	Dupl	somatic or paternally inherited*
21	17618519	17640790	22271	II_trimester	Dupl	somatic or paternally inherited*
21	17624130	17659790	35660	GD	Dupl	somatic
21	20296849	20342267	45418	normal term	Dupl	somatic
21	22821643	22922109	100466	II_trimester	Dupl	somatic or paternally inherited*
21	23655900	23661835	5935	II_trimester	Del	somatic or paternally inherited*
21	27163338	27216357	53019	SGA	Del	somatic
21	37348480	37452918	104438	II_trimester	Del	somatic or paternally inherited*
22	18886915	19008108	121193	normal term	Del	maternally inherited
22	19223352	19337633	114281	LGA	Del	somatic
22	19223352	19362142	138790	LGA	Del	somatic
22	19228736	19310812	82076	normal term	Del	somatic
22	25632266	25899939	267673	normal term	Dupl	somatic
22	29087040	29258551	171511	normal term	Del	somatic
22	36409990	36525981	115991	LGA	Del	somatic
22	36409990	36525981	115991	PE	Del	somatic
22	36409990	36525981	115991	PE	Del	somatic
22	36427483	36525981	98498	II_trimester	Del	somatic or paternally inherited*
22	36433816	36525981	92165	II_trimester	Del	somatic or paternally inherited*
22	36438470	36525981	87511	II_trimester	Del	somatic or paternally inherited*
22	40880692	41610059	729367	LGA	Del	somatic
22	46598306	46608917	10611	II_trimester	Del	somatic or paternally inherited*
22	50793661	50853626	59965	normal term	Del	somatic

b. Parental CNVs

Chr	Start	End	Length	Group	Type	Mother/father
1	1385211	1439671	54460	SGA	Del	father
1	1385211	1439671	54460	LGA	Del	mother
1	1619541	1664124	44583	LGA	Del	father
1	1627987	1664124	36137	I_trimester	Del	mother
1	2352146	2366316	14170	GD	Dupl	father
1	2358944	2369498	10554	SGA	Dupl	father
1	3411413	3431235	19822	PE	Dupl	mother
1	12880153	12913896	33743	LGA	Del	mother
1	15783771	15796808	13037	normal term	Del	mother
1	15794011	15814186	20175	normal term	Del	mother
1	25598276	25655538	57262	GD	Del	father

Chr	Start	End	Length	Group	Type	Mother/father
1	25598276	25655538	57262	II_trimester	Del	mother
1	25598276	25655538	57262	I_trimester	Del	mother
1	36367780	36387654	19874	GD	Del	father
1	41343608	41378076	34468	LGA	Dupl	mother
1	41343608	41378076	34468	PE	Dupl	father
1	45636892	45645069	8177	I_trimester	Del	mother
1	49928063	49990335	62272	LGA	Del	father
1	50863202	50874830	11628	SGA	Del	mother
1	59024435	59086221	61786	SGA	Del	mother
1	73011443	73086201	74758	LGA	Del	father
1	73959879	73993804	33925	SGA	Dupl	father
1	94867056	95134903	267847	normal term	Dupl	mother
1	97435890	97454547	18657	PE	Del	mother
1	102667388	102846147	178759	SGA	Del	mother
1	104129880	104211046	81166	normal term	Del	mother
1	104129880	104211046	81166	SGA	Del	mother
1	104129880	104211046	81166	LGA	Del	mother
1	104129880	104211046	81166	I_trimester	Del	mother
1	104153865	104211046	57181	normal term	Del	mother
1	104153865	104155944	2079	SGA	Del	father
1	104153865	104211046	57181	LGA	Del	father
1	104153865	104211046	57181	I_trimester	Del	mother
1	111926153	111934304	8151	PE	Del	mother
1	111926153	111944073	17920	GD	Del	mother
1	145625979	145762959	136980	SGA	Del	mother
1	145625979	145762959	136980	PE	Del	father
1	149039930	149201987	162057	normal term	Del	father
1	149039930	149201987	162057	normal term	Del	father
1	149039930	149201987	162057	LGA	Del	father
1	149039930	149201987	162057	LGA	Del	mother
1	149039930	149201987	162057	GD	Del	mother
1	149039930	149201987	162057	GD	Del	father
1	149060682	149201987	141305	GD	Del	father
1	158753406	158876921	123515	LGA	Del	father
1	161492587	161501525	8938	SGA	Del	father
1	191833819	191887735	53916	normal term	Del	mother
1	193648643	193684171	35528	GD	Del	mother
1	196823300	196892322	69022	SGA	Del	father
1	196823300	196887457	64157	LGA	Del	father
1	196823300	196862569	39269	GD	Del	mother
1	212999701	213011812	12111	SGA	Del	father
1	212999701	213011812	12111	I_trimester	Del	mother
1	231709404	231863204	153800	normal term	Dupl	father
1	231709404	231813134	103730	GD	Dupl	father
1	231711489	231813134	101645	GD	Dupl	mother
1	236984562	237128380	143818	LGA	Del	mother
1	239714774	239785165	70391	GD	Dupl	father

Chr	Start	End	Length	Group	Type	Mother/father
1	245636915	245647726	10811	normal term	Del	father
1	245636915	245647726	10811	SGA	Del	mother
1	245636915	245642219	5304	PE	Del	father
2	18674	189972	171298	GD	Dupl	mother
2	2864210	2916955	52745	normal term	Dupl	mother
2	4677905	5958997	1281092	PE	Dupl	father
2	13486778	13536772	49994	GD	Dupl	mother
2	14355721	14385992	30271	I_trimester	Dupl	mother
2	35470727	35597319	126592	GD	Del	father
2	35819686	36085059	265373	SGA	Del	father
2	39518512	39588797	70285	LGA	Dupl	mother
2	49857938	49865140	7202	PE	Del	father
2	50268228	50276061	7833	GD	Del	mother
2	51345474	51411126	65652	I_trimester	Del	mother
2	51926599	51926904	305	PE	Del	father
2	51926599	51926904	305	PE	Del	mother
2	51926599	51926904	305	normal term	Del	father
2	51926599	51926904	305	normal term	Del	father
2	51926599	51926904	305	normal term	Del	mother
2	51926599	51926904	305	normal term	Del	father
2	51926599	51926904	305	normal term	Del	father
2	51926599	51926904	305	LGA	Del	mother
2	51926599	51926904	305	LGA	Del	mother
2	51926599	51926904	305	LGA	Del	mother
2	51926599	51926904	305	LGA	Del	mother
2	51926599	51926904	305	PE	Del	mother
2	51926599	51926904	305	GD	Del	mother
2	51926599	51926904	305	GD	Del	father
2	51926599	51926904	305	GD	Del	mother
2	51926599	51926904	305	GD	Del	father
2	51926599	51926904	305	II_trimester	Del	mother
2	64695893	64703286	7393	LGA	Del	mother
2	76941049	76949101	8052	LGA	Del	mother
2	89427986	90240473	812487	PE	Dupl	father
2	89427986	90240473	812487	LGA	Dupl	mother
2	89427986	91820358	2392372	PE	Dupl	mother
2	90010895	90240473	229578	GD	Dupl	mother
2	90094257	90109261	15004	normal term	Del	father
2	90094257	90109261	15004	LGA	Del	father
2	90094257	90109261	15004	PE	Del	father
2	100055506	100062163	6657	I_trimester	Dupl	mother
2	100055763	100062163	6400	LGA	Dupl	father
2	100055763	100062163	6400	PE	Dupl	father
2	123818296	123878592	60296	I_trimester	Dupl	mother
2	135972093	136231459	259366	GD	Dupl	mother
2	142073760	142078003	4243	PE	Del	father
2	142999394	143043285	43891	SGA	Del	mother

Chr	Start	End	Length	Group	Type	Mother/father
2	154163944	154199490	35546	SGA	Dupl	father
2	154701808	155131619	429811	GD	Dupl	mother
2	160321788	160567537	245749	PE	Dupl	mother
2	184797826	184802609	4783	normal term	Del	father
2	184797826	184802609	4783	normal term	Del	father
2	184797826	184802609	4783	GD	Del	father
2	184797826	184802609	4783	II_trimester	Del	mother
2	184797826	184802609	4783	I_trimester	Del	mother
2	184797826	184802609	4783	I_trimester	Del	mother
2	188435643	188530578	94935	SGA	Del	mother
2	201843500	201971024	127524	normal term	Dupl	father
2	209034801	209050447	15646	SGA	Del	father
2	213173172	213192268	19096	SGA	Del	father
2	213183696	213192268	8572	normal term	Del	father
2	213183696	213192268	8572	normal term	Del	mother
2	213183696	213192268	8572	LGA	Del	mother
2	213183696	213190493	6797	GD	Del	father
2	213187034	213191389	4355	LGA	Del	father
2	213187034	213191389	4355	II_trimester	Del	mother
2	213188733	213190493	1760	normal term	Del	father
2	213635052	213657426	22374	LGA	Del	father
2	223563574	223588671	25097	GD	Del	father
2	223798501	223823141	24640	PE	Dupl	father
2	228243310	228258288	14978	PE	Dupl	mother
2	241558936	241576626	17690	SGA	Dupl	mother
2	241558936	241576626	17690	GD	Dupl	mother
2	242812080	243048760	236680	LGA	Del	mother
2	242873800	243048760	174960	normal term	Del	mother
2	242873800	243048760	174960	GD	Del	father
2	242917734	243048760	131026	LGA	Del	father
2	242917734	243048760	131026	PE	Del	father
2	242917734	243048760	131026	II_trimester	Del	mother
2	242919764	243048760	128996	GD	Del	father
2	242926381	243048760	122379	normal term	Del	father
3	61495	126209	64714	I_trimester	Dupl	mother
3	857325	1063826	206501	GD	Del	mother
3	1782524	1783885	1361	GD	Del	father
3	5391554	5438374	46820	normal term	Del	mother
3	8829872	8857963	28091	SGA	Dupl	father
3	20577473	20583674	6201	normal term	Dupl	father
3	22283291	22285562	2271	normal term	Del	mother
3	22283291	22285562	2271	SGA	Del	mother
3	28810588	28816082	5494	GD	Del	father
3	35826707	35902885	76178	PE	Dupl	mother
3	37979882	37986249	6367	PE	Del	father
3	37979882	37986249	6367	GD	Del	mother
3	37979882	37986249	6367	GD	Del	father

Chr	Start	End	Length	Group	Type	Mother/father
3	37979882	37986249	6367	II_trimester	Del	mother
3	37979882	37986249	6367	I_trimester	Del	mother
3	53033295	53035044	1749	normal term	Del	father
3	53033295	53035044	1749	normal term	Del	father
3	53033295	53035044	1749	LGA	Del	father
3	53033295	53035044	1749	LGA	Del	mother
3	53033295	53035044	1749	PE	Del	mother
3	53033295	53035044	1749	PE	Del	mother
3	62958569	62993083	34514	SGA	Del	father
3	62958569	62993083	34514	LGA	Del	father
3	65117947	65118762	815	normal term	Del	father
3	65191847	65214533	22686	normal term	Del	mother
3	65191847	65215804	23957	normal term	Del	father
3	65191847	65214533	22686	normal term	Del	mother
3	65191847	65214533	22686	SGA	Del	mother
3	65191847	65206583	14736	LGA	Del	mother
3	65191847	65212596	20749	PE	Del	mother
3	65191847	65214533	22686	PE	Del	father
3	65191847	65215804	23957	PE	Del	mother
3	65191847	65214533	22686	GD	Del	mother
3	65191847	65214533	22686	GD	Del	mother
3	65191847	65206583	14736	GD	Del	mother
3	65191847	65215804	23957	GD	Del	mother
3	65191847	65214533	22686	II_trimester	Del	mother
3	65191847	65214533	22686	II_trimester	Del	mother
3	65191847	65206583	14736	II_trimester	Del	mother
3	65191847	65214533	22686	I_trimester	Del	mother
3	65206583	65212596	6013	LGA	Dupl	father
3	66584922	66629765	44843	SGA	Dupl	father
3	75428675	75605157	176482	PE	Del	mother
3	75428675	75726925	298250	normal term	Del	father
3	75428675	75469493	40818	normal term	Del	mother
3	75428675	75726925	298250	PE	Del	mother
3	75428675	75605157	176482	GD	Del	mother
3	75428675	75726925	298250	GD	Del	father
3	89384566	89417171	32605	GD	Del	father
3	89384566	89417171	32605	I_trimester	Del	mother
3	96303888	96319964	16076	GD	Del	father
3	100351696	100437892	86196	PE	Dupl	father
3	100351696	100437892	86196	GD	Dupl	mother
3	128391789	128412024	20235	SGA	Dupl	mother
3	128391789	128412024	20235	SGA	Dupl	father
3	136149139	136260722	111583	I_trimester	Dupl	mother
3	148964692	148967335	2643	PE	Del	mother
3	151514590	151574286	59696	SGA	Del	mother
3	151514590	151546041	31451	GD	Del	mother
3	151514590	151546041	31451	II_trimester	Del	mother

Chr	Start	End	Length	Group	Type	Mother/father
3	151676322	151681018	4696	II_trimester	Del	mother
3	162131408	162142475	11067	normal term	Del	father
3	162131408	162142475	11067	SGA	Del	father
3	162131408	162142475	11067	SGA	Del	mother
3	162131408	162142475	11067	LGA	Del	mother
3	162131408	162142475	11067	LGA	Del	father
3	162131408	162142475	11067	PE	Del	mother
3	162131408	162142475	11067	PE	Del	mother
3	162131408	162142475	11067	GD	Del	mother
3	162131408	162142475	11067	GD	Del	father
3	162131408	162138826	7418	II_trimester	Del	mother
3	162132155	162142475	10320	SGA	Dupl	mother
3	162918387	163178778	260391	I_trimester	Del	mother
3	163178778	163263886	85108	normal term	Dupl	father
3	163661532	163664285	2753	SGA	Del	mother
3	165041116	165073006	31890	LGA	Del	father
3	165041116	165073006	31890	PE	Del	father
3	165041116	165073006	31890	PE	Del	mother
3	165041116	165073006	31890	I_trimester	Del	mother
3	173239453	173301037	61584	PE	Dupl	father
3	173239453	173309839	70386	LGA	Dupl	mother
3	175893082	175907342	14260	I_trimester	Del	mother
3	186386776	186411076	24300	normal term	Del	mother
3	186411076	186494422	83346	normal term	Dupl	mother
3	192305208	192312270	7062	GD	Del	father
3	194226486	194245931	19445	GD	Del	father
4	71566	134851	63285	II_trimester	Dupl	mother
4	2764088	2770772	6684	I_trimester	Del	mother
4	10398427	10400156	1729	normal term	Del	mother
4	16413099	16428830	15731	SGA	Del	mother
4	16413099	16428830	15731	GD	Del	father
4	22934689	23563103	628414	GD	Dupl	mother
4	25033008	25505932	472924	GD	Dupl	mother
4	35149660	35151237	1577	SGA	Del	mother
4	42697553	42708098	10545	II_trimester	Del	mother
4	42697553	42708098	10545	I_trimester	Del	mother
4	44976703	45002987	26284	I_trimester	Dupl	mother
4	45817327	45863268	45941	PE	Dupl	father
4	64066404	64080016	13612	II_trimester	Del	mother
4	64138341	64150666	12325	SGA	Del	mother
4	64138341	64150666	12325	GD	Del	father
4	64138341	64150666	12325	GD	Del	mother
4	64138341	64150666	12325	II_trimester	Del	mother
4	66584922	66629765	44843	normal term	Dupl	father
4	80160465	80598713	438248	normal term	Dupl	father
4	80871932	80943688	71756	SGA	Del	mother
4	86210281	86432381	222100	PE	Dupl	father

Chr	Start	End	Length	Group	Type	Mother/father
4	93786775	93948103	161328	GD	Del	father
4	97308914	97391366	82452	LGA	Del	mother
4	101568956	101616281	47325	LGA	Del	mother
4	108065738	108081536	15798	PE	Del	father
4	111079337	111187683	108346	SGA	Dupl	mother
4	121393821	121457685	63864	GD	Del	mother
4	122285263	122289863	4600	GD	Del	father
4	122324112	122333765	9653	normal term	Del	mother
4	129774375	129927804	153429	SGA	Dupl	mother
4	138103313	138190394	87081	II_trimester	Del	mother
4	154581208	154585922	4714	GD	Del	mother
4	158430547	158479507	48960	normal term	Del	father
4	161062144	161071059	8915	SGA	Del	father
4	161062144	161071059	8915	GD	Del	mother
4	161062144	161071059	8915	GD	Del	mother
4	161590091	161646978	56887	GD	Dupl	father
4	161945302	162009525	64223	LGA	Dupl	father
4	161952228	162009525	57297	normal term	Dupl	father
4	161952228	162003624	51396	SGA	Dupl	mother
4	161952228	162003624	51396	LGA	Dupl	mother
4	161952228	162003624	51396	GD	Dupl	mother
4	161952228	162003624	51396	GD	Dupl	father
4	161952228	162003624	51396	I_trimester	Dupl	mother
4	165576740	165587000	10260	PE	Del	father
4	165576740	165587000	10260	GD	Del	father
4	186400228	186414626	14398	GD	Del	father
4	189133207	189158163	24956	GD	Del	mother
4	190438606	190915650	477044	GD	Dupl	father
5	788646	851046	62400	SGA	Del	father
5	788646	840717	52071	PE	Del	father
5	788646	821726	33080	PE	Del	mother
5	788646	825338	36692	GD	Del	mother
5	7169851	7191074	21223	normal term	Del	father
5	7169851	7191074	21223	SGA	Del	mother
5	7178644	7191074	12430	PE	Del	mother
5	7179551	7191074	11523	LGA	Del	father
5	8708019	8744557	36538	normal term	Del	mother
5	8708019	8744557	36538	LGA	Del	mother
5	8708019	8744557	36538	PE	Del	mother
5	8708019	8744557	36538	GD	Del	mother
5	8708019	8744557	36538	I_trimester	Del	mother
5	8715360	8744557	29197	PE	Del	father
5	8715360	8744557	29197	I_trimester	Del	mother
5	8728874	8744557	15683	LGA	Del	father
5	9902403	9924597	22194	SGA	Del	mother
5	9902403	9924597	22194	PE	Del	mother
5	9902403	9924597	22194	GD	Del	mother

Chr	Start	End	Length	Group	Type	Mother/father
5	18819946	18913137	93191	normal term	Dupl	mother
5	32101400	32159517	58117	normal term	Dupl	mother
5	32101400	32159517	58117	GD	Dupl	mother
5	32107381	32159517	52136	normal term	Dupl	mother
5	32107381	32159517	52136	GD	Dupl	father
5	32107381	32159517	52136	I_trimester	Dupl	mother
5	68757774	68828296	70522	GD	Dupl	mother
5	70305696	70307464	1768	normal term	Del	father
5	70305696	70307464	1768	LGA	Del	mother
5	70305696	70307464	1768	GD	Del	father
5	70305696	70307464	1768	GD	Del	father
5	70305696	70307464	1768	I_trimester	Del	mother
5	94264817	94316007	51190	GD	Dupl	mother
5	97047653	97099320	51667	normal term	Del	father
5	97048466	97108559	60093	normal term	Del	mother
5	97048466	97108559	60093	SGA	Del	mother
5	97048466	97099320	50854	LGA	Del	father
5	97048466	97108559	60093	LGA	Del	mother
5	97048466	97108559	60093	LGA	Del	mother
5	97048466	97108559	60093	GD	Del	mother
5	97048466	97111523	63057	II_trimester	Del	mother
5	97048466	97108559	60093	II_trimester	Del	mother
5	97048466	97108559	60093	I_trimester	Del	mother
5	97933888	97939616	5728	I_trimester	Del	mother
5	98779530	98841255	61725	LGA	Del	father
5	99597529	99614601	17072	LGA	Del	mother
5	104069562	104406952	337390	LGA	Del	father
5	106223536	106260687	37151	SGA	Del	father
5	109363175	109384306	21131	SGA	Del	father
5	109363175	109375480	12305	SGA	Del	mother
5	113160490	113171928	11438	LGA	Del	father
5	113874136	113986223	112087	PE	Del	mother
5	117701468	117707373	5905	I_trimester	Del	mother
5	155477866	155484237	6371	SGA	Del	mother
5	155477866	155484237	6371	LGA	Del	mother
5	155477866	155484237	6371	LGA	Del	mother
5	177391049	177400550	9501	LGA	Del	mother
6	14839778	14845799	6021	PE	Del	mother
6	19045560	19048773	3213	normal term	Del	mother
6	19045560	19048773	3213	PE	Del	mother
6	27632581	27660508	27927	LGA	Dupl	father
6	29094696	29161435	66739	LGA	Del	mother
6	29869110	29896421	27311	SGA	Del	father
6	31221039	31228972	7933	GD	Del	father
6	31338528	31341357	2829	II_trimester	Del	mother
6	31355119	31453640	98521	PE	Dupl	father
6	32498834	32519005	20171	SGA	Del	mother

Chr	Start	End	Length	Group	Type	Mother/father
6	48329809	48352572	22763	II_trimester	Del	mother
6	55776585	56253790	477205	normal term	Del	father
6	55819362	55846527	27165	I_trimester	Del	mother
6	55828727	55846527	17800	normal term	Del	father
6	65828853	65910220	81367	normal term	Del	father
6	66554082	66622346	68264	I_trimester	Dupl	mother
6	66593133	66622346	29213	I_trimester	Dupl	mother
6	67017494	67044431	14341	normal term	Del	mother
6	67017494	67047294	29800	normal term	Del	mother
6	67017494	67039740	22246	PE	Del	father
6	67017494	67047294	29800	PE	Del	mother
6	67017494	67047294	29800	GD	Del	father
6	67017494	67047294	29800	GD	Del	mother
6	84553478	84698353	144875	normal term	Dupl	father
6	84553478	84698353	144875	LGA	Dupl	father
6	110396540	110411065	14525	LGA	Del	father
6	112955668	112968423	12755	SGA	Dupl	father
6	124404539	124474595	70056	normal term	Dupl	mother
6	124416847	124474595	57748	SGA	Dupl	father
6	129093272	129125184	31912	I_trimester	Dupl	mother
6	138909908	138929434	19526	PE	Del	mother
6	147670086	147723577	53491	SGA	Dupl	father
6	147685480	147723577	38097	I_trimester	Dupl	mother
6	151618235	151621611	3376	PE	Del	mother
6	154323909	154341558	17649	GD	Del	mother
6	161352698	161566909	214211	II_trimester	Dupl	mother
7	44935	63494	18559	GD	Dupl	mother
7	3609712	3627799	18087	I_trimester	Del	mother
7	9128070	9229882	101812	normal term	Del	mother
7	9636392	9847642	211250	GD	Del	mother
7	9722307	9805492	83185	normal term	Dupl	father
7	10819155	10945193	126038	GD	Del	father
7	12418602	12422721	4119	PE	Del	father
7	17425895	17453479	27584	normal term	Del	mother
7	17425895	17453479	27584	GD	Del	mother
7	19206436	19211709	5273	PE	Del	mother
7	19206436	19211709	5273	GD	Del	mother
7	21940960	22011292	70332	normal term	Dupl	father
7	33166070	33185898	19828	GD	Dupl	mother
7	43930775	43935301	4526	I_trimester	Del	mother
7	52129322	52131514	2192	PE	Dupl	father
7	52733291	52743803	10512	normal term	Dupl	mother
7	52733291	52743803	10512	LGA	Dupl	mother
7	52733291	52743803	10512	PE	Dupl	mother
7	52733291	52743803	10512	GD	Dupl	father
7	53198269	53206225	7956	SGA	Del	mother
7	57481104	57621951	140847	PE	Dupl	mother

Chr	Start	End	Length	Group	Type	Mother/father
7	61747794	62358419	610625	SGA	Dupl	mother
7	61747794	62838532	1090738	PE	Dupl	father
7	61994170	62729224	735054	PE	Dupl	mother
7	62035570	62047108	11538	I_trimester	Del	mother
7	76134203	76144560	10357	LGA	Dupl	father
7	76143705	76637441	493736	normal term	Dupl	father
7	76474914	76637441	162527	I_trimester	Dupl	mother
7	88588037	88596054	8017	normal term	Del	father
7	92055272	92069164	13892	GD	Del	mother
7	97933601	98157619	224018	I_trimester	Dupl	mother
7	108058574	108066326	7752	normal term	Del	father
7	109455705	109477177	21472	normal term	Dupl	father
7	109455705	109477177	21472	SGA	Dupl	father
7	111025948	111073710	47762	PE	Del	mother
7	111069946	111166165	96219	I_trimester	Dupl	mother
7	111251449	111288373	36924	PE	Del	mother
7	111433419	111630024	196605	I_trimester	Del	mother
7	141774290	141793419	19129	normal term	Del	father
7	141774290	141793419	19129	normal term	Del	father
7	141774290	141793419	19129	SGA	Del	father
7	141774290	141793419	19129	PE	Del	mother
7	141774290	141782731	8441	GD	Del	father
7	141774290	141793419	19129	GD	Del	father
7	141774290	141793419	19129	GD	Del	mother
7	142137119	142152369	15250	GD	Del	mother
7	145794079	145796758	2679	PE	Del	father
7	147117454	147145576	28122	SGA	Dupl	father
7	150553475	150560322	6847	normal term	Del	mother
7	151590285	151599503	9218	LGA	Del	father
7	151873853	152277531	403678	LGA	Dupl	father
7	158845384	159119486	274102	normal term	Dupl	mother
8	2142267	2158537	16270	I_trimester	Del	mother
8	3786543	3789272	2729	LGA	Del	father
8	5563282	5587923	24641	PE	Del	mother
8	5599399	5605087	5688	SGA	Del	father
8	5599399	5605087	5688	SGA	Del	mother
8	5599399	5605087	5688	LGA	Del	father
8	5599399	5608082	8683	PE	Del	mother
8	5599399	5607663	8264	PE	Del	father
8	5599399	5605087	5688	GD	Del	mother
8	5599399	5607797	8398	GD	Del	father
8	5599399	5605087	5688	GD	Del	father
8	5599399	5605087	5688	GD	Del	mother
8	5599399	5605087	5688	GD	Del	father
8	5599399	5605087	5688	I_trimester	Del	mother
8	5599399	5605087	5688	I_trimester	Del	mother
8	5601191	5605087	3896	normal term	Del	father

Chr	Start	End	Length	Group	Type	Mother/father
8	5601191	5605087	3896	GD	Del	father
8	8583109	8587589	4480	PE	Del	father
8	8583109	8587589	4480	GD	Del	mother
8	13380879	13536002	155123	GD	Dupl	father
8	15395144	15410250	15106	I_trimester	Del	mother
8	15899688	15939182	39494	PE	Dupl	mother
8	15948235	16025964	77729	normal term	Del	father
8	15948235	16025964	77729	LGA	Del	father
8	16262509	16268502	5993	normal term	Del	father
8	16262509	16268502	5993	PE	Del	mother
8	17580791	17581454	663	normal term	Del	father
8	17580791	17582137	1346	normal term	Del	mother
8	17580791	17581700	909	normal term	Del	mother
8	17580791	17582137	1346	SGA	Del	mother
8	17580791	17581454	663	LGA	Del	mother
8	17580791	17582137	1346	PE	Del	father
8	17580791	17581700	909	GD	Del	father
8	17580791	17582137	1346	II_trimester	Del	mother
8	20952060	21151009	198949	PE	Del	father
8	24604793	25028671	423878	GD	Dupl	father
8	40184872	40189549	4677	SGA	Del	father
8	40184872	40189549	4677	GD	Del	father
8	51227228	51230812	3584	GD	Del	father
8	83271395	83293872	22477	SGA	Del	mother
8	83271395	83293872	22477	PE	Del	mother
8	84876695	84890104	13409	GD	Dupl	mother
8	86923375	86990451	67076	GD	Del	father
8	90215649	90259170	43521	LGA	Del	father
8	92128840	92181214	52374	normal term	Del	mother
8	92128840	92198211	69371	normal term	Del	mother
8	92128840	92183658	54818	SGA	Del	mother
8	92275768	92295114	19346	SGA	Del	mother
8	92275768	92295114	19346	SGA	Del	mother
8	96959819	96969356	9537	LGA	Del	father
8	137679805	137857327	177522	GD	Del	father
8	137688230	137857327	169097	normal term	Del	mother
9	46587	274108	227521	II_trimester	Dupl	mother
9	354334	355793	1459	normal term	Del	mother
9	395250	707463	312213	II_trimester	Dupl	mother
9	1447011	1450428	3417	LGA	Del	mother
9	3628376	3747376	119000	LGA	Del	mother
9	5306824	5335470	28646	GD	Del	father
9	8582229	8610513	28284	LGA	Dupl	father
9	11414180	11463799	49619	PE	Del	father
9	12046842	12163583	116741	GD	Del	father
9	24502737	24515111	12374	normal term	Del	father
9	24502737	24515111	12374	SGA	Del	mother

Chr	Start	End	Length	Group	Type	Mother/father
9	24502737	24515111	12374	PE	Del	father
9	24754015	24785247	31232	SGA	Del	father
9	28309788	28342179	32391	GD	Del	mother
9	29919239	29928182	8943	GD	Dupl	father
9	30181745	30222006	40261	SGA	Dupl	father
9	72100118	72115711	15593	SGA	Del	mother
9	73907625	73909871	2246	normal term	Dupl	father
9	73907625	73909871	2246	normal term	Dupl	mother
9	86203246	86206343	3097	SGA	Del	mother
9	86203246	86206343	3097	LGA	Del	father
9	86203246	86206343	3097	LGA	Del	father
9	86203246	86206343	3097	PE	Del	father
9	86203246	86206343	3097	PE	Del	mother
9	86203246	86206343	3097	PE	Del	father
9	86203246	86206343	3097	II_trimester	Del	mother
9	94396059	94398847	2788	II_trimester	Del	mother
9	115938318	116029174	90856	normal term	Dupl	father
10	13056587	13060410	3823	SGA	Del	mother
10	13056587	13060410	3823	SGA	Del	father
10	13056587	13058458	1871	II_trimester	Del	mother
10	20842031	20857365	15334	LGA	Del	mother
10	20842031	20857365	15334	GD	Del	mother
10	36909064	36949132	40068	LGA	Dupl	father
10	42682133	42827951	145818	PE	Del	father
10	45209441	45359483	150042	SGA	Dupl	mother
10	46283686	47703869	1420183	GD	Dupl	mother
10	47049547	47940417	890870	PE	Dupl	mother
10	47049547	47940417	890870	II_trimester	Dupl	mother
10	47543322	47940417	397095	PE	Dupl	mother
10	47543322	47940417	397095	LGA	Dupl	mother
10	47543322	47703869	160547	II_trimester	Dupl	mother
10	47543322	47703869	160547	I_trimester	Dupl	mother
10	53207634	53211449	3815	GD	Del	father
10	55735463	55779915	44452	SGA	Del	mother
10	57401749	57460416	58667	PE	Del	mother
10	58768319	58916759	148440	GD	Del	father
10	68078481	68114481	36000	SGA	Del	mother
10	68078481	68114481	36000	LGA	Del	mother
10	68078481	68114481	36000	LGA	Del	father
10	68078481	68113595	35114	GD	Del	father
10	68078481	68114481	36000	II_trimester	Del	mother
10	68078481	68114481	36000	I_trimester	Del	mother
10	78457588	78459211	1623	II_trimester	Del	mother
10	82879719	82890180	10461	LGA	Del	father
10	82879719	82890180	10461	GD	Del	father
10	82879719	82890180	10461	II_trimester	Del	mother
10	82879719	82890180	10461	II_trimester	Del	mother

Chr	Start	End	Length	Group	Type	Mother/father
10	82879719	82892288	12569	II_trimester	Del	mother
10	83468658	83485025	16367	I_trimester	Del	mother
10	83944705	83959179	14474	SGA	Del	father
10	83944705	83959179	14474	PE	Del	mother
10	87954602	87959049	4447	GD	Del	mother
10	96499710	96563757	64047	I_trimester	Del	mother
10	107618048	107681447	63399	SGA	Del	mother
10	107984320	108015496	31176	normal term	Del	mother
10	107984320	108015496	31176	LGA	Del	father
10	114846649	114847723	1074	normal term	Del	mother
10	122079594	122094271	14677	II_trimester	Del	mother
10	134589631	134669463	79832	normal term	Dupl	father
10	135217175	135369532	152357	normal term	Dupl	mother
10	135252347	135378802	126455	PE	Dupl	mother
10	135252347	135378802	126455	II_trimester	Dupl	mother
10	135252347	135378802	126455	I_trimester	Dupl	mother
11	264391	396308	131917	SGA	Dupl	mother
11	394005	419906	25901	SGA	Dupl	mother
11	8959020	8964938	5918	PE	Del	father
11	18135642	18138153	2511	normal term	Del	mother
11	18949220	18956690	7470	GD	Del	father
11	25712300	25720706	8406	LGA	Del	father
11	25712300	25720706	8406	I_trimester	Del	mother
11	34458230	34460711	2481	GD	Dupl	father
11	34458230	34460704	2474	GD	Dupl	father
11	37768477	37891672	123195	I_trimester	Dupl	mother
11	42151229	42185849	34620	PE	Dupl	mother
11	49977336	50668400	691064	PE	Dupl	mother
11	59204920	59244608	39688	PE	Del	father
11	67373669	67588012	214343	I_trimester	Dupl	mother
11	73638858	73708521	69663	PE	Dupl	father
11	81498152	81517261	19109	GD	Del	mother
11	81503992	81517261	13269	SGA	Del	father
11	81503992	81526573	22581	SGA	Del	mother
11	81503992	81517261	13269	LGA	Del	mother
11	81503992	81517261	13269	LGA	Del	mother
11	81503992	81517261	13269	LGA	Del	father
11	81503992	81517261	13269	PE	Del	mother
11	81503992	81517261	13269	GD	Del	mother
11	81505463	81517261	11798	GD	Del	mother
11	81505463	81517261	11798	II_trimester	Del	mother
11	82858667	82876270	17603	normal term	Dupl	father
11	82865518	82876270	10752	PE	Dupl	father
11	88372708	88380551	7843	normal term	Del	mother
11	96192004	96243894	51890	normal term	Dupl	father
11	97852498	98523074	670576	PE	Del	father
11	99377407	99574689	197282	SGA	Dupl	father

Chr	Start	End	Length	Group	Type	Mother/father
11	99525126	99566782	41656	GD	Del	mother
11	104366089	104372318	6229	SGA	Del	mother
11	104754500	104761271	6771	LGA	Del	mother
11	107653081	107683596	30515	GD	Dupl	father
11	107659024	107670228	11204	GD	Dupl	father
11	109111782	109156782	45000	GD	Del	father
11	120557343	120630452	73109	SGA	Del	mother
11	120557343	120630452	73109	GD	Del	mother
11	122686128	122749214	63086	GD	Del	mother
11	134347974	134720173	372199	LGA	Dupl	father
12	2245636	2252924	7288	normal term	Del	mother
12	2245636	2252924	7288	LGA	Del	mother
12	2245636	2252924	7288	PE	Del	father
12	2245636	2252924	7288	GD	Del	father
12	2245636	2252924	7288	II_trimester	Del	mother
12	2245636	2252924	7288	II_trimester	Del	mother
12	2245636	2252924	7288	I_trimester	Del	mother
12	2245636	2252924	7288	I_trimester	Del	mother
12	7930171	8146162	215991	LGA	Dupl	mother
12	7930171	8102386	172215	PE	Dupl	father
12	7993316	8125745	132429	I_trimester	Dupl	mother
12	8000912	8114429	113517	LGA	Dupl	father
12	8003758	8123306	119548	II_trimester	Del	mother
12	16002622	16018886	16264	SGA	Del	mother
12	16295061	16480949	185888	GD	Del	mother
12	21293334	21298702	5368	GD	Dupl	father
12	25648676	25654445	5769	PE	Del	mother
12	31259372	31415204	155832	normal term	Dupl	father
12	31259372	31406907	147535	SGA	Dupl	father
12	31259372	31409778	150406	LGA	Dupl	father
12	31259372	31409579	150207	PE	Dupl	mother
12	31259372	31409778	150406	GD	Dupl	mother
12	31266287	31408696	142409	PE	Dupl	mother
12	31266287	31406907	140620	SGA	Dupl	father
12	52845952	52859985	14033	SGA	Del	mother
12	52845952	52862614	16662	I_trimester	Del	mother
12	62269717	62272569	2852	normal term	Del	mother
12	63942649	64118558	175909	PE	Dupl	mother
12	70679864	70681814	1950	normal term	Del	father
12	70680393	70681814	1421	II_trimester	Del	mother
12	70680393	70681814	1421	I_trimester	Del	mother
12	80155600	80160230	4630	GD	Dupl	father
12	83171936	83206342	34406	SGA	Del	mother
12	100002686	100008773	6087	II_trimester	Del	mother
12	109552849	109712475	159626	normal term	Dupl	father
12	125260645	125321461	60816	PE	Dupl	father
12	129226773	129266496	39723	SGA	Dupl	mother

Chr	Start	End	Length	Group	Type	Mother/father
12	129228874	129234104	5230	PE	Del	mother
12	129228874	129234104	5230	I_trimester	Del	mother
12	131730735	131825125	94390	GD	Del	father
13	20470071	20509959	39888	LGA	Dupl	mother
13	23357136	23515553	158417	LGA	Dupl	father
13	23546238	23553518	7280	normal term	Del	father
13	27015106	27017442	2336	I_trimester	Del	mother
13	45634138	45910139	276001	GD	Dupl	father
13	58593657	58599031	5374	PE	Del	father
13	58593657	58599031	5374	II_trimester	Del	mother
13	64343819	64387507	43688	GD	Dupl	mother
13	69240121	69267981	27860	SGA	Del	mother
13	69240121	69267981	27860	PE	Del	mother
13	69247022	69267981	20959	normal term	Del	mother
13	69247022	69267981	20959	SGA	Del	mother
13	69247022	69276739	29717	LGA	Del	father
13	69247022	69267981	20959	PE	Del	mother
13	69247022	69267981	20959	GD	Del	mother
13	69247022	69267981	20959	II_trimester	Del	mother
13	69247022	69267981	20959	II_trimester	Del	mother
13	69247022	69267981	20959	I_trimester	Del	mother
13	70565868	70578513	12645	I_trimester	Del	mother
13	70744882	70778217	33335	I_trimester	Del	mother
13	80691352	80696432	5080	PE	Dupl	father
13	81919202	81952150	32948	LGA	Dupl	father
13	83788742	83791615	2873	PE	Del	father
13	83788742	83793975	5233	GD	Del	father
13	84103707	84157927	54220	GD	Del	father
13	84103707	84157927	54220	GD	Del	mother
13	84103707	84157927	54220	I_trimester	Del	mother
13	84103707	84157927	54220	I_trimester	Del	mother
13	85868323	85904426	36103	PE	Dupl	mother
13	89770384	89840239	69855	SGA	Del	mother
13	90387429	90490883	103454	II_trimester	Dupl	mother
13	95933660	96035883	102223	LGA	Dupl	mother
13	95933660	95983387	49727	GD	Dupl	mother
13	95999739	96035883	36144	GD	Dupl	mother
13	103689469	103699213	9744	LGA	Dupl	mother
13	106326623	106866163	539540	II_trimester	Dupl	mother
14	19327823	20426742	1098919	LGA	Dupl	father
14	19327823	20626291	1298468	I_trimester	Dupl	mother
14	21371774	21393289	21515	LGA	Del	mother
14	22376027	22737977	361950	normal term	Del	father
14	22719680	22795126	75446	LGA	Del	father
14	24453366	24484060	30694	SGA	Del	mother
14	24453366	24484060	30694	SGA	Del	father
14	24453366	24484060	30694	LGA	Del	mother

Chr	Start	End	Length	Group	Type	Mother/father
14	24453366	24484060	30694	LGA	Del	father
14	35110512	35160860	50348	LGA	Del	father
14	40855032	40858428	3396	PE	Del	father
14	41853509	41884448	30939	normal term	Del	father
14	46749460	46759568	10108	LGA	Del	mother
14	58771632	58799361	27729	I_trimester	Dupl	mother
14	62654698	62734866	80168	SGA	Del	mother
14	86284754	86310164	25410	LGA	Del	father
14	86287347	86310164	22817	SGA	Del	father
14	86287347	86332917	45570	SGA	Del	mother
14	86287347	86310164	22817	PE	Del	father
14	86287347	86310164	22817	GD	Del	mother
14	86458414	86481388	22974	GD	Del	father
14	86460915	86481388	20473	SGA	Del	mother
14	88399488	88419358	19870	II_trimester	Del	mother
14	90758891	90802050	43159	SGA	Dupl	father
14	90788934	90802050	13116	normal term	Dupl	father
14	90788934	90802050	13116	SGA	Dupl	father
14	90788934	90802050	13116	PE	Dupl	father
14	90788934	90802050	13116	GD	Dupl	mother
14	90788934	90802050	13116	GD	Dupl	father
14	105951611	107287663	1336052	GD	Dupl	mother
14	105998544	107287663	1289119	II_trimester	Dupl	mother
14	106326623	106866163	539540	normal term	Dupl	mother
15	20612840	22652330	2039490	LGA	Dupl	father
15	23077857	23114374	36517	SGA	Del	mother
15	23084443	23114374	29931	PE	Del	mother
15	24055488	24061497	6009	normal term	Del	mother
15	24055488	24061497	6009	LGA	Del	mother
15	24055488	24061497	6009	II_trimester	Del	mother
15	24357212	24472002	114790	GD	Del	father
15	24594816	24742652	147836	PE	Dupl	father
15	24594816	24668007	73191	LGA	Dupl	mother
15	24594816	24668007	73191	GD	Dupl	mother
15	24606732	24668007	61275	II_trimester	Dupl	mother
15	25420978	25430243	9265	I_trimester	Del	mother
15	30369914	31089526	719612	PE	Del	mother
15	32509892	32514341	4449	GD	Dupl	father
15	34760648	34800595	39947	normal term	Del	father
15	34760648	34800595	39947	GD	Del	mother
15	34760648	34800595	39947	II_trimester	Del	mother
15	34760648	34800595	39947	I_trimester	Del	mother
15	35509148	35562582	53434	SGA	Del	mother
15	36034333	36036487	2154	II_trimester	Del	mother
15	37437248	37501850	64602	SGA	Del	father
15	42159110	42192040	32930	PE	Dupl	father
15	43893818	43939642	45824	normal term	Del	father

Chr	Start	End	Length	Group	Type	Mother/father
15	43893818	43939642	45824	PE	Del	mother
15	53518466	53593018	74552	LGA	Dupl	mother
15	61687640	61692518	4878	GD	Del	mother
15	69696249	69735319	39070	LGA	Dupl	mother
15	81977465	82088114	110649	PE	Dupl	father
15	86511690	86521483	9793	SGA	Del	father
15	86511690	86521483	9793	SGA	Del	mother
15	87831146	87870024	38878	PE	Del	mother
15	102338427	102366849	28422	II_trimester	Dupl	mother
16	10149904	10164599	14695	PE	Del	father
16	12686917	12730650	43733	normal term	Del	father
16	15154898	15499057	344159	PE	Del	father
16	20521796	20532514	10718	normal term	Del	mother
16	20916286	20986506	70220	LGA	Del	mother
16	22435811	22702769	266958	SGA	Del	mother
16	28603889	28620752	16863	PE	Dupl	father
16	28615243	28620752	5509	LGA	Dupl	mother
16	31220869	31226691	5822	SGA	Del	mother
16	32165782	33795687	1629905	PE	Del	mother
16	32165782	33557021	1391239	GD	Del	father
16	32608438	33557021	948583	LGA	Del	mother
16	32608438	32648969	40531	GD	Del	mother
16	34303923	34817176	513253	I_trimester	Dupl	mother
16	63520776	63582751	61975	I_trimester	Del	mother
16	72723613	72785303	61690	GD	Del	father
16	75571249	75580985	9736	normal term	Dupl	mother
16	79252324	79273682	21358	I_trimester	Del	mother
17	1116991	1239946	122955	PE	Del	mother
17	3506043	3582239	76196	II_trimester	Del	mother
17	6291337	6309355	18018	GD	Dupl	mother
17	6295959	6312267	16308	normal term	Dupl	father
17	6301562	6311397	9835	GD	Dupl	mother
17	19510081	19519444	9363	LGA	Del	father
17	30858969	30866889	7920	PE	Del	mother
17	33684035	33768199	84164	LGA	Del	father
17	33684035	33768199	84164	PE	Del	mother
17	33684035	33768199	84164	II_trimester	Del	mother
17	34456073	34476396	20323	GD	Dupl	mother
17	44131767	44580136	448369	GD	Dupl	mother
17	44161441	44580136	418695	LGA	Dupl	father
17	44161441	44368212	206771	LGA	Dupl	mother
17	44169808	44368212	198404	normal term	Dupl	mother
17	44169808	44580136	410328	PE	Dupl	mother
17	44169808	44580136	410328	normal term	Dupl	father
17	44169808	44368212	198404	SGA	Dupl	mother
17	44169808	44368212	198404	LGA	Dupl	mother
17	44169808	44580136	410328	LGA	Dupl	father

Chr	Start	End	Length	Group	Type	Mother/father
17	44169808	44580136	410328	GD	Dupl	father
17	44169808	44580136	410328	GD	Dupl	father
17	44169808	44580136	410328	GD	Dupl	father
17	44169808	44580136	410328	II_trimester	Dupl	mother
17	50949615	51314354	364739	PE	Del	mother
17	50949615	51314354	364739	GD	Del	father
17	54160465	54172277	11812	normal term	Del	father
17	54160465	54172277	11812	LGA	Del	mother
17	54160465	54172277	11812	PE	Del	mother
17	60109041	60137085	28044	normal term	Del	mother
17	64384076	64504644	120568	SGA	Del	father
17	64410915	64482151	71236	LGA	Del	father
17	66566063	66581506	15443	LGA	Del	mother
17	77365534	77392001	26467	GD	Dupl	father
17	77367698	77396482	28784	SGA	Dupl	father
17	77367698	77394037	26339	LGA	Dupl	father
17	77367698	77394037	26339	PE	Dupl	father
18	1725758	1841469	115711	SGA	Del	mother
18	1725758	1839339	113581	I_trimester	Del	mother
18	10433382	10439153	5771	LGA	Dupl	father
18	10913663	10919989	6326	normal term	Del	father
18	10915570	10924184	8614	LGA	Del	mother
18	27815320	27815968	648	PE	Del	father
18	27815320	27815968	648	PE	Del	father
18	30901989	31009751	107762	SGA	Dupl	mother
18	55935987	55937109	1122	II_trimester	Del	mother
18	58101865	58120273	18408	GD	Del	mother
18	63196448	63218786	22338	GD	Dupl	father
18	63849680	63866787	17107	PE	Dupl	father
18	63909072	63912698	3626	SGA	Del	mother
18	65248521	65690189	441668	LGA	Dupl	father
18	66747568	66755508	7940	normal term	Del	father
18	66747568	66755508	7940	LGA	Del	father
18	66747568	66755508	7940	LGA	Del	mother
18	66747568	66755508	7940	LGA	Del	father
18	66747568	66755508	7940	PE	Del	mother
18	66747568	66755508	7940	PE	Del	father
18	66747568	66755508	7940	I_trimester	Del	mother
18	67209141	67217271	8130	normal term	Del	father
18	67209141	67217271	8130	SGA	Del	mother
18	67209141	67217271	8130	LGA	Del	father
18	67209141	67217271	8130	PE	Del	father
19	1781879	1792997	11118	PE	Del	mother
19	5563342	5606259	42917	PE	Del	mother
19	6958134	7018869	60735	GD	Del	father
19	20626179	20707568	81389	normal term	Del	mother
19	20626179	20707568	81389	normal term	Del	father

Chr	Start	End	Length	Group	Type	Mother/father
19	20626179	20707568	81389	SGA	Del	father
19	20626179	20707568	81389	SGA	Del	mother
19	20626179	20707568	81389	SGA	Del	father
19	20626179	20707568	81389	PE	Del	mother
19	20626179	20707568	81389	PE	Del	father
19	20657781	20707568	49787	SGA	Del	father
19	20663314	20707568	44254	GD	Del	mother
19	20682055	20707568	25513	LGA	Del	mother
19	20691114	20707568	16454	normal term	Del	father
19	20691114	20707568	16454	normal term	Del	father
19	20691114	20707568	16454	normal term	Del	mother
19	20691114	20707568	16454	SGA	Del	father
19	20691114	20707568	16454	PE	Del	mother
19	20691114	20707568	16454	PE	Del	mother
19	20691114	20707568	16454	GD	Del	father
19	20691114	20707568	16454	GD	Del	mother
19	20691114	20707568	16454	GD	Del	father
19	20691114	20707568	16454	II_trimester	Del	mother
19	20691114	20707568	16454	II_trimester	Del	mother
19	20691114	20707568	16454	I_trimester	Del	mother
19	20691114	20707568	16454	I_trimester	Del	mother
19	20691114	20707568	16454	I_trimester	Del	mother
19	35587635	35597215	9580	GD	Del	mother
19	35587635	35600510	12875	II_trimester	Del	mother
19	41350895	41379148	28253	GD	Dupl	mother
19	41356751	41379148	22397	PE	Del	father
19	41356751	41379148	22397	LGA	Del	father
19	41356751	41379148	22397	GD	Del	father
19	43270565	43699208	428643	normal term	Del	mother
19	43328006	43556843	228837	PE	Del	mother
19	43328006	43537378	209372	normal term	Del	father
19	43328006	43559330	231324	normal term	Del	mother
19	43328006	43556843	228837	SGA	Del	mother
19	43371294	43699208	327914	I_trimester	Del	mother
19	43372386	43468660	96274	PE	Del	mother
19	43372386	43802290	429904	GD	Del	mother
19	43635684	43699208	63524	PE	Del	mother
19	43666697	43835770	169073	I_trimester	Del	mother
19	50526896	50905042	378146	normal term	Dupl	mother
19	51260989	51263510	2521	LGA	Dupl	mother
19	52273257	52617298	344041	GD	Dupl	mother
20	7957766	7962072	4306	PE	Dupl	father
20	12807016	12889314	82298	I_trimester	Dupl	mother
20	14794361	14931186	136825	I_trimester	Del	mother
20	31806588	31814348	7760	PE	Del	mother
20	37026342	37045533	19191	SGA	Dupl	father
20	44351421	44378173	26752	GD	Del	father

Chr	Start	End	Length	Group	Type	Mother/father
20	58956161	58962376	6215	PE	Dupl	father
20	61393873	61412466	18593	PE	Del	mother
21	10827533	10913441	85908	LGA	Del	mother
21	21916116	21918415	2299	normal term	Del	mother
21	24020223	24043069	22846	SGA	Del	father
21	35027142	35056247	29105	PE	Del	mother
21	44821867	44853859	31992	II_trimester	Dupl	mother
22	18844632	19008108	163476	normal term	Del	mother
22	22314463	22573637	259174	LGA	Dupl	father
22	25629682	25998204	368522	LGA	Dupl	father
22	25669569	25914593	245024	GD	Del	father
22	25832419	25843884	11465	normal term	Del	mother
22	25832419	25843884	11465	normal term	Del	mother
22	25832419	25843884	11465	SGA	Del	mother
22	25832419	25843884	11465	SGA	Del	mother
22	25832419	25843884	11465	SGA	Del	father
22	25832419	25843884	11465	LGA	Del	father
22	25832419	25843884	11465	PE	Del	mother
22	25832419	25843884	11465	PE	Del	mother
22	25832419	25843884	11465	II_trimester	Del	mother
22	39428919	39445556	16637	normal term	Del	mother
22	40049410	40065322	15912	normal term	Del	mother

* paternal genotype data not available. Chr, chromosome; SGA, small-for-gestational age; GD, gestational diabetes; PE, preeclampsia; LGA, large-for-gestational age; Dupl, duplication; Del, deletion.

Supplementary Table 4. Characteristics of maternal and paternal CNVs

Maternal blood DNA samples							
		**Normal term**	**SGA**	**LGA**	**PE**	**GD**	**Total**
**No of samples**		8	8	8	8	8	40
**No of CNVs per sample**	all	8.1 (8.0)	9.4 (8.0)	8.3 (8.5)	10.4 (10.5)	11.1 (10.0)	9.5 (9.0)
	dupl	1.9 (1.0)	1.6 (1.0)	2.3 (2.0)	2.4 (2.5)	3.9 (4.0)	2.4 (2.0)
	del	6.3 (7.0)	7.8 (7.0)	6.0 (6.0)	8.0 (7.5)	7.3 (7.0)	7.1 (7.0)
**Cumulative span of all CNVs per sample, Mb**	all	0.6 (0.6)	0.5 (0.4)	0.6 (0.3)	1.5 (1.2)	1.2 (0.7)	0.8 (0.5)
	dupl	0.3 (0.1)	0.2 (0.1)	0.3 (0.2)	0.8 (0.7)	0.8 (0.4)	0.5 (0.3)
	del	0.3 (0.2)	0.3 (0.3)	0.3 (0.3)	0.6 (0.4)	0.3 (0.3)	0.3 (0.3)
**CNV length across samples, kb**	all	69.2 (24.3)	55.9 (22.6)	76.2 (23.5)	140.2 (24.6)	104.5 (28.3)	88.4 (24.3)
	dupl	149.5 (83.3)	125.1 (107.8)	134.1 (70.3)	354.2 (142.4)	216.8 (58.1)	208.4 (82.8)
	del	45.1 (18.4)	41.4 (21.1)	54.5 (14.0)	76.6 (19.3)	44.4 (21.6)	47.3 (17.6)
**CNV size range, kb**	all	0.3; 539.5	1.3; 610.6	0.3; 948.6	0.3; 2,392.4	0.3; 1,420.2	0.3; 2,392.4
	dupl	2.2; 539.5	10.3; 610.6	2.5; 812.5	10.5; 2,392.4	9.8; 1,420.2	2.2; 2,392.4
	del	0.3; 428.6	1.3; 267.0	0.3; 948.6	0.3; 1,629.9	0.3; 429.9	0.3; 1,629.9
**Ratio (loss/gain)**		3.3 (7.0)	4.8 (7.0)	2.7 (3.0)	3.4 (3.0)	1.9 (1.8)	2.9 (3.5)
Paternal blood DNA samples							
		**Normal term**	**SGA**	**LGA**	**PE**	**GD**	**Total**
**No of samples**		8	7	7	8	8	38
**No of CNVs per sample**	all	9.0 (10.5)	7.4 (7.0)	10.1 (9.0)	8.5 (8.5)	11.4 (10.5)	9.3 (9.0)
	dupl	2.8 (2.5)	2.7 (3.0)	3.3 (4.0)	3.3 (3.5)	3.1 (3.0)	3.0 (3.0)
	del	6.3 (7.0)	4.7 (4.0)	6.9 (6.0)	5.3 (5.5)	8.3 (7.0)	6.3 (6.0)
**Cumulative span of all CNVs per sample, Mb**	all	0.7 (0.7)	0.3 (0.3)	1.2 (1.0)	0.8 (0.9)	1.0 (0.8)	0.8 (0.6)
	dupl	0.3 (0.3)	0.1 (0.1)	0.9 (0.9)	0.6 (0.3)	0.4 (0.2)	0.5 (0.2)
	del	0.3 (0.1)	0.2 (0.2)	0.3 (0.3)	0.3 (0.2)	0.6 (0.4)	0.3 (0.2)
**CNV length across samples, kb**	all	73.6 (19.1)	42.9 (27.7)	122.0 (31.9)	97.4 (17.0)	86.7 (19.9)	86.7 (22.8)
	dupl	123.8 (81.5)	51.4 (33.9)	288.2 (144.9)	171.5 (40.2)	122.2 (30.5)	155.2 (51.4)
	del	51.5 (12.1)	38.0 (21.1)	42.3 (23.9)	51.5 (9.3)	73.3 (17.8)	53.8 (16.5)
**CNV size range, kb**	all	0.3; 493.7	2.1; 265.4	1.7; 2,039.5	0.3; 1281.1	0.3; 1391.2	0.3; 2,039.5
	dupl	2.2; 493.7	10.6; 197.3	5.8; 2,039.5	2.2; 1281.1	2.5; 477.0	2.2; 2,039.5
	del	0.3; 477.2	2.1; 265.4	1.7; 337.4	0.3; 670.6	0.3; 1,391.2	0.3; 1,391.2
**Ratio (loss/gain)**		2.3 (2.8)	1.7 (1.3)	2.1 (1.5)	1.6 (1.6)	2.6 (2.3)	2.1 (2.0)

No, number; SGA, small-for-gestational age; GD, gestational diabetes; PE, preeclampsia; LGA, large-for-gestational age; all, duplication and deletion CNVs; dupl, duplication; del, deletion.

Supplementary Table 5. Pathway analysis of all somatic and inherited placental and parental CNVs

a. Placental somatic CNVs

Somatic duplications in the placental genome								
**category_ID**	**category_type**	**category_name**	**FDR_p-value**	**n_genes_in_category**	**n_genes_in_query**	**n_common_genes**	**%_genes_category**	**common_genes**
GO:0009952	BP	anterior/posterior pattern specification	1.34E-05	202	1186	21	10.4%	*SKI,HTT,HOXA1,HOXA2,HOXA3,HOXA4,HOXA5,HOXA6,HOXA7,HOXA10,HOXA11,RP11-834C11.14,HOXC13,HOXC11,HOXC10,HOXC6,HOXC9,HOXC8,HOXC5,MYF6,MYF5*
GO:0007156	BP	homophilic cell adhesion	3.48E-05	139	1186	17	12.2%	*ROBO2,FAT4,PCDHA1,PCDHA2,PCDHA3,PCDHA4,PCDHA5,PCDHA6,PCDHA7,PCDHA8,PCDHA9,PCDHA10,PCDHA11,PCDHA12,PCDHA13,PCDH15,CDH19*
GO:0048706	BP	embryonic skeletal system development	2.33E-04	122	1186	15	12.3%	*COL11A1,HOXA1,HOXA2,HOXA3,HOXA4,HOXA5,HOXA6,HOXA7,HOXA11,RP11-834C11.14,HOXC11,HOXC6,HOXC9,HOXC5,MYF5*
GO:0005509	MF	calcium ion binding	2.43E-04	688	1186	39	5.7%	*SDF4,PLCH2,MEGF6,PEF1,RYR2,NRXN1,LRP1B,GCA,TTN,ATP2B2,SLIT2,FAT4,FSTL5,EDIL3,MCTP1,PCDHA1,PCDHA2,PCDHA3,PCDHA4,PCDHA5,PCDHA6,PCDHA7,PCDHA8,PCDHA9,PCDHA10,PCDHA11,PCDHA12,PCDHA13,MICU3,PCDH15,C2CD5,NELL2,SYT1,JAG2,RAB11FIP3,RHOT2,RHBDL1,CDH19,PLCB4*
GO:0072562	CC	blood microparticle	7.35E-04	133	1186	15	11.3%	*CPSF3L,ALB,CFB,CFB,C4A,C4B,POTEG,IGHA2,IGHG4,IGHG2,IGHA1,IGHG1,IGHG3,IGHD,IGHM*
GO:0048704	BP	embryonic skeletal system morphogenesis	2.13E-03	91	1186	12	13.2%	*COL11A1,HOXA1,HOXA2,HOXA3,HOXA4,HOXA5,HOXA6,HOXA7,HOXA11,HOXC11,HOXC9,MYF5*
MI:hsa-miR-210	mi	MI:hsa-miR-210	3.90E-03	649	1186	34	5.2%	*C1ORF222,C1ORF86,PEX10,MEGF6,RP11-181G12.2,LINC00982,COL16A1,GPR17,DUSP28,CAPN10,GAK,FGFRL1,RNF212,LRPAP1,FGF10,UQCC2,IP6K3,GRIK2,STXBP5,ZNF467,TIGD5,CARD9,GRM5,OR4K14,IGHV3-13,IGHV3-16,IGHV3-23,IGHV3-35,IGHV3-38,IGHV3-66,IGHA2,FBXL16,PSG4,SPO11*
GO:0006956	BP	complement activation	3.00E-02	64	1186	9	14.1%	*C2,CFB,C4A,C4B,IGHG4,IGHG2,IGHG1,IGHG3*

Somatic deletions in the placental genome								
**category_ID**	**category_type**	**category_name**	**FDR_p-value**	**n_genes_in_category**	**n_genes_in_query**	**n_common_genes**	**%_genes_category**	**common_genes**
GO:0022402	BP	cell cycle process	3.49E-05	1163	1294	61	5.2%	*CDK11A,KHDRBS1,MACF1,NSL1,SPAST,BIRC6,MTA3,MSH2,ACTR2,SKIL,SENP2,TP63,PLK4,JMY,CDC23,CDC25C,KATNA1,LATS1,NUP43,CDK13,MPLKIP,RINT1,ZC3HC1,VCPIP1,EYA1,HAUS6,PPP6C,CDC123,DNA2,TACC2,NUP98,RAD52,KIAA0391,PSMA6,POLE2,BMP4,INO80,OIP5,NUSAP1,HAUS2,STARD9,MEIOB,PAPD5,IST1,TXNL4B,NCOR1,CENPV,TAOK1,CRLF3,MED1,CDC6,SEPT4,TEX14,RAD51C,TRIM37,SKA2,RPS6KB1,PPM1D,CLTCL1,CHEK2,EP300*
GO:0035639	MF	purine ribonucleoside triphosphate binding	4.60E-05	1855	1294	84	4.5%	*CDK11A,NMNAT1,KIF1B,CMPK1,EPRS,IARS2,NVL,KIF3C,RAB10,SPAST,NLRC4,MSH2,RAB1A,ACTR2,ULK4,VPRBP,RAD54L2,DNAH12,ARF4,EPHA3,RAP2B,PRKCI,PAK2,DCK,HSPA4L,PLK4,NAIP,PAPD4,SAR1B,DDX46,MDN1,CDK19,SGK1,KATNA1,LATS1,RAC1,CDK13,SRPK2,UBE2H,C8ORF44-SGK3,SGK3,MCMDC2,RRAGA,UBE2R2,KIF27,HSPA5,PRKG1,DNA2,GLUD1,RHOG,RRM1,PIK3C2A,ATL3,MARK2,RHOD,RAB6A,RAB39A,DGKA,PCCA,KIAA0391,PSMA6,INO80,STARD9,TRPM7,SMG1,MAPK3,SEPHS2,AARS,DDX19B,RP11-529K1.3,DDX19A,UBE2G1,ERAL1,TAOK1,ATAD5,CDC6,SEPT4,TEX14,RAD51C,DHX40,TUBD1,RPS6KB1,RIOK3,CHEK2*
GO:1902589	BP	single-organism organelle organization	1.15E-04	1738	1294	79	4.5%	*CDK11A,NSL1,ARID4B,TBCE,DPY30,SPAST,BIRC6,MSH2,ACTR2,TMSB10,TRIP12,VPRBP,EPHA3,DPPA2,PRKCI,YEATS2,PAK2,PLK4,JMY,JADE2,SAR1B,SEC24A,CDC23,CDC25C,NSD1,KATNA1,LATS1,NUP43,RAC1,CDK13,MPLKIP,ZC3HC1,VCPIP1,EYA1,HAUS6,ABI1,PRKG1,DNA2,TET1,TACC2,RHOG,KDM2A,RAB39A,RAD52,EEA1,POLE2,BMP4,SYNE2,INO80,CHP1,OIP5,NUSAP1,RTF1,HAUS2,STARD9,TRPM7,MEIOB,COQ7,CLN3,CLN3,MAPK3,PAPD5,TXNL4B,NCOR1,CENPV,TEFM,NF1,CDC6,TEX14,RAD51C,PPM1E,TRIM37,SKA2,TUBD1,PHF20,NCOA3*

								,CLTCL1,CHEK2,EP300
GO:0032550	MF	purine ribonucleoside binding	2.33E-04	1861	1294	82	4.4%	*CDK11A,NMNAT1,KIF1B,CMPK1,EPRS,IARS2,NVL,KIF3C,RAB10,SPAST,NLRC4,MSH2,RAB1A,ACTR2,ULK4,VPRBP,RAD54L2,DNAH12,ARF4,EPHA3,RAP2B,PRKCI,PAK2,DCK,HSPA4L,PLK4,NAIP,PAPD4,SAR1B,DDX46,MDN1,CDK19,SGK1,KATNA1,LATS1,RAC1,CDK13,SRPK2,UBE2H,C8ORF44-SGK3,SGK3,MCMDC2,RRAGA,UBE2R2,KIF27,HSPA5,PRKG1,DNA2,GLUD1,RHOG,RRM1,PIK3C2A,ATL3,MARK2,RHOD,RAB6A,RAB39A,DGKA,PCCA,INO80,STARD9,TRPM7,SMG1,MAPK3,SEPHS2,AARS,DDX19B,RP11-529K1.3,DDX19A,UBE2G1,ERAL1,TAOK1,ATAD5,CDC6,SEPT4,TEX14,RAD51C,DHX40,TUBD1,RPS6KB1,RIOK3,CHEK2*
GO:0007017	BP	microtubule-based process	1.87E-03	498	1294	32	6.4%	*KIF1B,MACF1,TBCE,LYST,KIF3C,MEMO1,SPAST,RAB1A,ULK4,DNAH12,EPHA3,PLK4,KATNA1,EYA1,HAUS6,KIF27,TACC2,RAB6A,INO80,CHP1,NUSAP1,HAUS2,STARD9,DYX1C1,CLN3,CLN3,NCOR1,TEX14,TRIM37,SKA2,TUBD1,CHEK2*
GO:0043231	CC	intracellular membrane-bounded organelle	2.14E-03	10046	1294	296	2.9%	*CDK11A,NMNAT1,KIF1B,SPEN,USP48,SRRM1,CLIC4,PTP4A2,KHDRBS1,KPNA6,ZBTB8B,ZBTB8A,ZBTB8OS,AKIRIN1,NDUFS5,MACF1,CMPK1,COA7,ECHDC2,SCP2,PPAP2B,TAF13,RABGAP1L,NSL1,TATDN3,FLVCR1,IARS2,DEGS1,NVL,CNIH4,TOMM20,RBM34,ARID4B,B3GALNT2,ZNF670,ZNF695,RAB10,MEMO1,DPY30,SPAST,SLC30A6,YIPF4,BIRC6,COX7A2L,KCNG3,MTA3,MSH2,RAB1A,MBD5,TRIP12,SH3BP5,TRAK1,QRICH1,VPRBP,RAD54L2,SFMBT1,HESX1,APPL1,PDE12,ARF4,DENND6A,EPHA3,DPPA2,DPPA4,RAP2B,PHC3,PRKCI,SKIL,TBL1XR1,YEATS2,SENP2,KNG1,TP63,LEPREL1,PAK2,SENP5,UTP3,GRSF1,MOB1B,DCK,HSPA4L,PLK4,MFSD8,LRBA,RPS3A,SH3D19,OCLN,GTF2H2C,SMN2,SMN1,DMGDH,JMY,PAPD4,ALDH7A1,PHAX,LMNB1,JADE2,SAR1B,SEC24A,CAMLG,DDX46,PCBD2,CDC23,GFRA3,CDC25C,ATP6V0E1,CREBRF,NSD1,CASP8AP2,MDN1,C6ORF203,GTF3C6,RPF2,SGK1,PCMT1,KATNA1,NUP43,FAM220A,RAC1,TRA2A,CDK13,*

								MPLKIP,SUGCT,SRPK2,RINT1,ZC3HC1,KLHDC10,MTUS1,SNTG1,ARMC1,MTFR1,PTTG3P,MYBL1,VCPIP1,C8ORF44-SGK3,C8ORF44,SGK3,TCF24,EYA1,TPD52,RRAGA,PLIN2,DENND4C,RPS6,ACER2,UBAP1,SCAI,RABEPK,HSPA5,GAPVD1,FNBP1,ABI1,PRKG1,HNRNPH3,RUFY2,DNA2,SLC25A16,TET1,CCAR1,STOX1,GLUD1,LCOR,ARHGAP19-SLIT1,ARHGAP19,ATE1,NSMCE4A,TACC2,NUP98,PGAP2,STIM1,RRM1,DENND5A,IPO7,ZNF143,PIK3C2A,NUCB2,CTD-2132H18.3,PLA2G16,ATL3,RTN3,MARK2,RCOR2,KDM2A,RAB6A,MRPL48,RAB39A,RAD52,ERC1,OLR1,GABARAPL1,MUC19,WIBG,EEA1,PSPC1,ZMYM5,ZMYM2,NBEA,PCCA,KIAA0391,PSMA6,POLE2,SGPP1,SYNE2,SNRPN,INO80,CHP1,OIP5,NUSAP1,NDUFAF1,RTF1,ZNF106,SNAP23,HAUS2,STARD9,SPPL2A,DYX1C1,DPP8,PTPLAD1,FAHD1,MEIOB,ATF7IP2,PARN,BFAR,SMG1,COQ7,CLN3,CLN3,MAPK3,ZNF771,PAPD5,CNOT1,GOT2,NFATC3,DDX19B,DDX19A,ST3GAL2,PHLPP2,AP1G1,ATXN1L,IST1,ZNF821,DHODH,TXNL4B,NCOR1,PIGL,CENPV,ERAL1,FLOT2,PHF12,NUFIP2,ATAD5,TEFM,ADAP2,NF1,MED1,CDC6,KPNB1,NPEPPS,MTMR4,SEPT4,RAD51C,PPM1E,TRIM37,YPEL2,PTRH2,VMP1,TUBD1,RPS6KB1,CA4,USP32,APPBP2,PPM1D,BCAS3,TRAPPC8,TICAM1,PLIN3,ZNF317,CYP2A6,CYP2A7,SULT2B1,PHF20,ZMYND8,NCOA3,CLTCL1,HIRA,CHEK2,HSCB,XBP1,RBFOX2,MKL1,SLC25A17,XPNPEP3,RBX1,EP300,L3MBTL2,PPP6R2
GO:0007067	BP	mitosis	3.04E-03	364	1294	26	7.1%	*CDK11A,NSL1,BIRC6,CDC23,CDC25C,KATNA1,LATS1,NUP43,CDK13,MPLKIP,ZC3HC1,VCPIP1,HAUS6,BMP4,INO80,OIP5,NUSAP1,HAUS2,PAPD5,TXNL4B,CENPV,CDC6,TEX14,SKA2,CLTCL1,CHEK2*

GO:0005829	CC	cytosol	4.51E-03	2664	1294	102	3.8%	*FBLIM1,SRRM1,CLIC4,CMPK1,NSL1,EPRS,BPNT1,GGPS1,LYST,KIF3C,MEMO1,NLRC4,RAB1A,ACTR2,APPL1,ARF4,RAP2B,PRKCI,TP63,PAK2,MOB1B,DCK,PLK4,RPS3A,SH3D19,OCLN,SMN2,SMN1,BHMT,ALDH7A1,PHAX,SAR1B,SEC24A,CDC23,CDC25C,RPL26L1,AMD1,SGK1,LATS1,NUP43,RAC1,ASL,C8ORF44-SGK3,SGK3,DENND4C,RPS6,UBAP1,PPP6C,GAPVD1,ABI1,PRKG1,PIK3AP1,ARHGAP19-SLIT1,ARHGAP19,NUP98,RHOG,RRM1,PIK3C2A,NUCB2,PLA2G16,RHOD,RAB6A,ERC1,DGKA,EEA1,ZMYM2,NBEA,PCCA,KIAA0391,PSMA6,CHP1,ZNF106,HAUS2,EDC3,FAHD1,PARN,SMG1,MAPK3,DCTPP1,CNOT1,NFATC3,AARS,PHLPP2,AP1G1,IST1,TAOK1,RNF135,WIPF2,CDC6,KPNB1,NPEPPS,MTMR4,TRIM37,SKA2,SMG8,PTRH2,RPS6KB1,DPP9,TICAM1,SULT2B1,ST13,RBX1*
GO:0005874	CC	microtubule	2.79E-02	362	1294	24	6.6%	*KIF1B,MACF1,WDR47,TBCE,KIF3C,SPAST,DNAH12,TBL1XR1,KATNA1,MTUS1,HAUS6,KIF27,STIM1,CLMP,GABARAPL1,INO80,NUSAP1,HAUS2,NCOR1,SKA2,TUBD1,APPBP2,CYP2A6,CYP2A7*

b. Placental inherited CNVs

Inherited duplications in the placental genome

No significant enrichment

Inherited deletions in the placental genome

**category_ID**	**category_type**	**category_name**	**FDR_p-value**	**n_genes_in_category**	**n_genes_in_query**	**n_common_genes**	**%_genes_category**	**common_genes**
GO:0007565	BP	female pregnancy	1.54E-06	174	117	9	5.2%	*RLN1,PSG1,PSG6,PSG7,PSG11,PSG2,PSG5,PSG4,PSG9*
GO:0016160	MF	amylase activity	8.06E-04	6	117	3	50.0%	*AMY2A,AMY1A,MGAM*
MI:hsa-miR-19a	mi	MI:hsa-miR-19a	1.29E-02	766	117	9	1.2%	*DAB1,TACSTD2,CFHR2,SFMBT1,PCDH15,PSG1,PSG6,PSG2,PSG9*
GO:0008389	MF	coumarin 7-hydroxylase activity	1.61E-02	2	117	2	100.0%	*CYP2A6,CYP2A7*
GO:0004970	MF	ionotropic glutamate receptor activity	3.22E-02	18	117	3	16.7%	*GRID2,GRIK4,GRIN2A*

GO:0005576	CC	extracellular region	3.27E-02	3655	117	23	0.6%	*CELA2A,CELA2B,TACSTD2,AMY2A,AMY1A,CFHR2,CFHR4,COL21A1,MSR1,MTUS1,RLN1,CCDC3,PCDH15,NRG3,PSG1,PSG6,PSG7,PSG11,PSG2,PSG5,PSG4,PSG9,DGCR6*
GO:0005234	MF	extracellular-glutamate-gated ion channel activity	3.81E-02	19	117	3	15.8%	*GRID2,GRIK4,GRIN2A*
GO:0046226	BP	coumarin catabolic process	4.82E-02	3	117	2	66.7%	*CYP2A6,CYP2A7*

c. Somatic CNVs: normal term pregnancy

Duplications specific to placentas from normal, uncomplicated term pregnancies

**category_ID**	**category_type**	**category_name**	**FDR_p-value**	**n_genes_in_category**	**n_genes_in_query**	**n_common_genes**	**%_genes_category**	**common_genes**
GO:0009952	BP	anterior/posterior pattern specification	2.81E-12	202	306	18	8.9%	*HTT,HOXA1,HOXA2,HOXA3,HOXA4,HOXA5,HOXA6,HOXA7,HOXA10,HOXA11,RP11-834C11.14,HOXC13,HOXC11,HOXC10,HOXC6,HOXC9,HOXC8,HOXC5*
GO:0048706	BP	embryonic skeletal system development	2.98E-09	122	306	13	10.7%	*HOXA1,HOXA2,HOXA3,HOXA4,HOXA5,HOXA6,HOXA7,HOXA11,RP11-834C11.14,HOXC11,HOXC6,HOXC9,HOXC5*
GO:0043565	MF	sequence-specific DNA binding	1.20E-07	724	306	24	3.3%	*HOXA1,HOXA2,HOXA3,HOXA4,HOXA5,HOXA6,HOXA7,HOXA9,RP1-170O19.20,HOXA10,HOXA11,HOXA13,MAFA,RP11-834C11.14,HOXC13,HOXC12,HOXC11,HOXC10,HOXC6,HOXC9,HOXC8,HOXC4,HOXC5,CEBPG*
GO:0048704	BP	embryonic skeletal system morphogenesis	9.15E-07	91	306	10	11.0%	*HOXA1,HOXA2,HOXA3,HOXA4,HOXA5,HOXA6,HOXA7,HOXA11,HOXC11,HOXC9*
GO:0003700	MF	sequence-specific DNA binding transcription factor activity	3.10E-04	1166	306	25	2.1%	*FBXW7,HOXA1,HOXA2,HOXA3,HOXA4,HOXA5,HOXA6,HOXA7,HOXA9,RP1-170O19.20,HOXA10,HOXA11,HOXA13,MAFA,RP11-834C11.14,HOXC13,HOXC12,HOXC11,HOXC10,HOXC6,HOXC9,HOXC8,HOXC4,HOXC5,CEBPG*
GO:0007565	BP	female pregnancy	4.83E-04	174	306	10	5.7%	*ADRA2C,PSG8,PSG1,PSG6,PSG7,PSG11,PSG2,PSG5,PSG4,PSG9*

Deletions specific to placentas from normal, uncomplicated term pregnancies								
**category_ID**	**category_ type**	**category_name**	**FDR_p-value**	**n_genes_in _category**	**n_genes_in _query**	**n_common _genes**	**%_genes _category**	***common_genes***
GO:0008389	MF	coumarin 7-hydroxylase activity	5.00E-02	2	265	2	100.0%	*CYP2A6,CYP2A7*

d. Somatic CNVs: complicated pregnancies

Duplications specific to placentas from pregnancies resulting in Small-for-Gestational Age (SGA) newborns

No significant enrichment

Deletions specific to placentas from pregnancies resulting in Small-for-Gestational Age (SGA) newborns

No significant enrichment

Duplications specific to placentas from pregnancies resulting in Large-for-Gestational Age (LGA) newborns								
**category_ID**	**category_ type**	**category_name**	**FDR_p-value**	**n_genes_in _category**	**n_genes_in _query**	**n_common _genes**	**%_genes_cate gory**	**common_genes**
MI:hsa-miR-615-5p	mi	MI:hsa-miR-615-5p	4.26E-02	794	129	10	1.3%	*TNFRSF18,SDF4,B3GALT6,FAM132A,PUSL1,TAS1R3,PEF1,WDR86,DPYSL4,BCAT1*
GO:0045979	BP	positive regulation of nucleoside metabolic process	4.90E-02	3	129	2	66.7%	*PID1,ARHGEF10*

Deletions specific to placentas from pregnancies resulting in Large-for-Gestational Age (LGA) newborns								
**category_ID**	**category_ type**	**category_name**	**FDR_p-value**	**n_genes_in _category**	**n_genes_in _query**	**n_common _genes**	**%_genes_cate gory**	**common_genes**
GO:0051453	BP	regulation of intracellular pH	6.37E-03	35	331	5	14.3%	*ATP6V0E1,RAB39A,CHP1,CLN3*
GO:1902589	BP	single-organism organelle organization	1.52E-02	1738	331	28	1.6%	*CDK11A,TMSB10,TRIP12,EPHA3,PRKCI,YEATS2,PLK4,NSD1,CDK13,MPLKIP,ABI1,TACC2,RAB39A,EEA1,BMP4,INO80,CHP1,OIP5,NUSAP1,RTF1,CLN3,CLN3,MAPK3,PAPD5,TXNL4B,CDC6,PHF20,EP300*
MI:hsa-miR-301a	mi	MI:hsa-miR-301a	2.62E-02	764	331	15	2.0%	*USP48,WDR47,METTL6,HSPA4L,C4ORF29,RPL26L1,SGK1,C9ORF64,EEA1,PCCA,NFATC3,PHF20,MCHR1,DNAJB7,EP300*

category_ID	category_type	category_name	FDR_p-value	n_genes_in_category	n_genes_in_query	n_common_genes	%_genes_category	*common_genes*
Duplications specific to placentas from preeclamptic (PE) pregnancies								
GO:0004984	MF	olfactory receptor activity	1.10E-16	420	161	17	4.0%	*OR11H12,OR4N2,OR4Q3,OR4M1,OR4K2,OR4K5,OR4K1,OR4K15,OR4Q2,OR4K14,OR4K13,OR4L1,OR4K17,OR4N5,OR11G2,OR11H2,OR4K3*
GO:0071944	CC	cell periphery	5.14E-03	4640	161	21	0.5%	*GCA,KCNH7,ITPR3,OR11H12,POTEG,OR4N2,OR4Q3,OR4M1,OR4K2,OR4K5,OR4K1,OR4K15,OR4Q2,OR4K14,OR4K13,OR4L1,OR4K17,OR4N5,OR11G2,OR11H2,OR4K3*

Deletions specific to placentas from preeclamptic (PE) pregnancies

No significant enrichment

Duplications specific to placentas from gestational diabetes (GD) pregnancies

category_ID	category_type	category_name	FDR_p-value	n_genes_in_category	n_genes_in_query	n_common_genes	%_genes_category	common_genes
GO:0005041	MF	low-density lipoprotein receptor activity	2.87E-02	10	32	2	20.0%	*LRP1B,CD36*

Deletions specific to placentas from gestational diabetes (GD) pregnancies

category_ID	category_type	category_name	FDR_p-value	n_genes_in_category	n_genes_in_query	n_common_genes	%_genes_category	common_genes
GO:0045747	BP	positive regulation of Notch signaling pathway	5.37E-03	22	9	2	9.1%	*TP63,EYA1*
GO:0048645	BP	organ formation	4.11E-02	60	9	2	3.3%	*TP63,EYA1*

e. Parental blood CNVs

Duplications in the parental genome

category_ID	category_type	category_name	FDR_p-value	n_genes_in_category	n_genes_in_query	n_common_genes	%_genes_category	common_genes
GO:0003823	MF	antigen binding	1.58E-05	90	1079	11	12.2%	*IGKV3-20,MICA,IGHA2,IGHE,IGHG4,IGHG2,IGHA1,IGHG1,IGHG3,IGHD,IGHM*
GO:0004984	MF	olfactory receptor activity	4.49E-05	420	1079	21	5.0%	*OR4C12,OR11H12,OR4N2,OR4Q3,OR4M1,OR4K2,OR4K5,OR4K1,OR11H2,OR4K3,OR4K15,OR4Q2,OR4K14,OR4K13,OR4L1,OR4K17,OR4N5,OR4M2,OR4N4,OR4F6,OR4F15*
GO:0042571	CC	immunoglobulin complex, circulating	1.88E-04	5	1079	4	80.0%	*IGKV3-20,IGHA2,IGHA1,IGHM*
GO:0072562	CC	blood microparticle	9.27E-04	133	1079	11	8.3%	*IGKV3-20,KNG1,POTEG,IGHA2,IGHG4,IGHG2,IGHA1,IGHG1,IGHG3,IGHD,IGHM*
GO:0009411	BP	response to UV	1.93E-02	117	1079	9	7.7%	*MAP4K3,REV1,ZRANB3,RAD18,MAP3K4,CAT,CRIP1,CRIP1,POLD1*
GO:0071944	CC	cell periphery	2.74E-02	4640	1079	82	1.8%	*PLCH2,F3,TSNAX-DISC1,DISC1,CHRM3,FAM110C,IGKV3-20,TM4SF20,GPR35,CAV3,GPR128,NLGN1,KNG1,JADE1,GOLPH3,OCLN,MICA,NKAIN2,STXBP5,RP11-361F15.2,BBS9,UPK3B,BAIAP2L1,CNTNAP2,VIPR2,KANK1,PTPRD,TRPM3,SLC31A1,FAM21C,SYT15,NPY4R,ANXA8L1,INPP5A,SPRN,NLRP6,IFITM5,IFITM2,IFITM1,IFITM3,PKP3,ANO9,CAT,OR4C12,ACY3,CNTN5,SLC2A3,SLCO1B1,SCARB1,ABCC4,SLC10A2,OR11H12,POTEG,OR4N2,OR4Q3,OR4M1,OR4K2,OR4K5,OR4K1,OR11H2,OR4K3,OR4K15,OR4Q2,OR4K14,OR4K13,OR4L1,OR4K17,OR4N5,IGHD,IGHM,POTEB,OR4M2,OR4N4,SPTBN5,EHD4,OR4F6,OR4F15,TMEM231,KCNC3,FPR1,FPR2,FPR3*

Deletions in the parental genome								
**category_ID**	**category_type**	**category_name**	**FDR_p-value**	**n_genes_in_category**	**n_genes_in_query**	**n_common_genes**	**%_genes_category**	**common_genes**
GO:0005231	MF	excitatory extracellular ligand-gated ion channel activity	1.21E-03	51	616	7	13.7%	*GRID2,GRID1,GRIK4,GRIN2A,SHPK,P2RX5-TAX1BP3,P2RX5*
GO:0005216	MF	ion channel activity	1.63E-03	373	616	16	4.3%	*GRID2,OPRM1,AOC1,SLC26A7,GRID1,GRIK4,CACNA1C,CHRFAM7A,CATSPER2,GRIN2A,SHPK,P2RX5-TAX1BP3,P2RX5,PIEZO2,TPTE,CACNA1I*
GO:0007215	BP	glutamate receptor signaling pathway	4.19E-03	61	616	7	11.5%	*NRXN1,GRID2,OPRM1,GRID1,GRM5,GRIK4,GRIN2A*
GO:0008066	MF	glutamate receptor activity	9.32E-03	26	616	5	19.2%	*GRID2,GRID1,GRM5,GRIK4,GRIN2A*
GO:0007565	BP	female pregnancy	1.65E-02	174	616	10	5.7%	*RLN1,PSG8,PSG1,PSG6,PSG7,PSG11,PSG2,PSG5,PSG4,PSG9*
GO:0070383	BP	DNA cytosine deamination	1.80E-02	5	616	3	60.0%	*APOBEC3D,APOBEC3F,APOBEC3G*
GO:0030534	BP	adult behavior	1.81E-02	139	616	9	6.5%	*NRXN1,FGF12,OPRM1,PCDH15,CACNA1C,EPS8,KLHL1,CTNS,NTSR1*
GO:0007626	BP	locomotory behavior	2.23E-02	180	616	10	5.6%	*FGF12,OPRM1,APBA1,PCDH15,GRM5,CACNA1C,EPS8,KLHL1,CTNS,NTSR1*
GO:0045869	BP	negative regulation of single stranded viral RNA replication via double stranded DNA intermediate	3.58E-02	6	616	3	50.0%	*APOBEC3D,APOBEC3F,APOBEC3G*
GO:0016160	MF	amylase activity	3.58E-02	6	616	3	50.0%	*AMY2A,AMY1A,MGAM*

BP, biological process; MF, molecular function; CC, cellular component; mi, miRBase microRNAs.

Supplementary Table 6. Functional profiling of placental CNVs with WebGestalt. The list of genes in somatic and inherited duplication and deletion CNVs was subjected to functional enrichment analysis using WebGestalt software[1] and included Gene Ontology (GO) functional category.

**Significantly overrepresented functional categories**		**Enrichment analysis**	
**GO:ID**	**Name**	**Pathway genes (%)**	**FDR *P*-value**
Somatic duplication CNVs			
0009952	anterior/posterior pattern specification	10.6	2.32×10^{-6}
0007156	homophilic cell adhesion	12.1	1.13×10^{-5}
0003002	regionalization	8.1	1.45×10^{-5}
0001501	skeletal system development	7.2	1.75×10^{-5}
0016337	cell-cell adhesion	6.9	3.44×10^{-5}
0003823	antigen binding	16.7	6.00×10^{-5}
0005509	calcium ion binding	5.4	1.00×10^{-4}
0006958	complement activation, classical pathway	18	3.00×10^{-4}
0006956	complement activation	15.6	3.00×10^{-4}
0072376	protein activation cascade	13.3	4.00×10^{-4}
Somatic deletion CNVs			
0006996	organelle organization	4.4	7.18×10^{-6}
0048285	organelle fission	7.7	2.06×10^{-5}
0000279	M phase	6.5	3.37×10^{-5}
0007067	mitosis	7.6	3.37×10^{-5}
0000280	nuclear division	7.6	3.37×10^{-5}
0000087	M phase of mitotic cell cycle	7.4	4.31×10^{-5}
0043227	membrane-bounded organelle	2.9	4.86×10^{-5}
0043231	intracellular membrane-bounded organelle	2.9	4.86×10^{-5}
0044424	intracellular part	2.7	5.69×10^{-5}
0005622	intracellular	2.7	5.69×10^{-5}
Inherited duplication CNVs			
no functional categories significantly enriched			
Inherited deletion CNVs			
0007565	female pregnancy	6.1	3.92×10^{-8}
0044706	multi-multicellular organism process	5.2	7.52×10^{-8}
0005576	extracellular region	0.9	3.58×10^{-5}
0005234	extracellular-glutamate-gated ion channel activity	15.8	4.00×10^{-4}
0004970	ionotropic glutamate receptor activity	16.7	4.00×10^{-4}
0016160	amylase activity	50	5.00×10^{-4}
0008066	glutamate receptor activity	11.5	5.00×10^{-4}
0007215	glutamate receptor signaling pathway	6.3	1.38×10^{-2}
0032501	multicellular organismal process	0.5	1.38×10^{-2}
0005231	excitatory extracellular ligand-gated ion channel activity	6.3	2.70×10^{-3}

Supplementary Table 7. Recurrent somatic CNVs detected in at least three placentas. Start and end positions indicate overlapping regions of all the carriers.

CNVR ID	Chr	Start pos	End pos	Chr band	Length	Type	No of carriers	Groups	Genes
CNVR_1	1	3423467	3531594	1p36.32	108127	Dupl	3	2 LGA,1 norm	*MEGF6,MIR551A*
CNVR_2	1	10009925	10120793	1p36.22	110868	Del	6	2 LGA, 2 PE, 1 GD, 1 norm	*NMNAT1,RBP7,UBE4B,RP11-84A14.4,MIR5697,RP11-807G9.2,AL590639.1,RP11-496H15.2,PGAM1P11*
CNVR_3	1	53180565	53218380	1p32.3	37815	Del	3	2 norm, 1 LGA	*ZYG11B*
CNVR_4	1	73386470	73482160	1p31.1	95690	Dupl	3	3 norm	-
CNVR_5	1	98292912	98316285	1p21.3	23373	Dupl	4	2 PE, 2 norm	*DPYD*
CNVR_6	1	103342391	103467615	1p21.1	125224	Dupl	5	2 SGA, 2 norm, 1 GD	*COL11A1*
CNVR_7	1	113026556	113040420	1p13.2	13864	Del	3	2 norm, 1 GD	*WNT2B*
CNVR_8	1	232575539	232584061	1q42.2	8522	Dupl	3	2 PE, 1 norm	*SIPA1L2*
CNVR_9	2	32148619	32611512	2p22.3	462893	Del	4	3 PE, 1 norm	*CTC-336P14.1,AL121655.1,RP11-563N4.1,DDX50P1,RNU6-647P,AL133245.2,MEMO1,BIRC6-AS1,DPY30,SPAST,SLC30A6,NLRC4,YIPF4,BIRC6*
CNVR_10	2	34699812	34726904	2p22.3	27092	Del	4	2 LGA, 1 SGA, 1 norm	*AC073218.1*
CNVR_11	2	38296889	38303094	2p22.2	6205	Dupl	3	2 PE, 1 GD	*CYP1B1-AS1,CYP1B1*
CNVR_12	2	42690479	42881534	2p21	191055	Del	3	1 LGA, 1 GD, 1 norm	*AC025750.6,AC025750.5,AC025750.7,Y_RNA,KCNG3,MTA3*
CNVR_13	2	51926599	51926904	2p16.3	305	Del/Dupl	1/2	2 norm, 1 GD	*AC007682.1*
CNVR_14	2	188388310	188545602	2q32.1	157292	Dupl	3	2 GD, 1 PE	*AC007319.1,TFPI*
CNVR_15	2	242795350	243048760	2q37.3	253410	Dupl	4	2 LGA, 2 PE	*AC131097.3,AC093642.3,AC093642.4,AC093642.6,RP11-341N2.1,AC093642.5,PDCD1,CXXC11,AC131097.4*
CNVR_16	3	51527117	51577966	3p21.2	50849	Del	11	4 LGA, 3 PE, 2 SGA, 2 norm	*VPRBP,RAD54L2*
CNVR_17	3	125897537	125973658	3q21.3	76121	Dupl	3	1 SGA, 1 GD, 1 norm	*ALDH1L1-AS2,ALDH1L1*
CNVR_18	3	162130691	162142475	3q26.1	11784	Del	3	2 PE, 1 SGA	-
CNVR_19	3	189738195	189739056	3q28	861	Del	8	3 norm, 2 PE, 2 SGA, 1 GD	*LEPREL1*
CNVR_20	4	32446724	32495165	4p15.1	48441	Dupl	6	3 norm, 2 SGA, 1 GD	-

CNVR ID	Chr	Start pos	End pos	Chr band	Length	Type	No of carriers	Groups	Genes
CNVR_21	4	137542931	137698875	4q28.3	155944	Dupl	6	3 norm, 1 SGA, 1 PE, 1 GD	*AC093875.1*
CNVR_22	4	146025645	146055117	4q31.21	29472	Dupl	5	2 SGA, 2 norm, 1 GD	*ABCE1,OTUD4*
CNVR_23	5	7885226	7920843	5p15.31	35617	Dupl	3	1 LGA, 1 PE, 1 norm	*MTRR*
CNVR_24	5	24779565	24806889	5p14.1	27324	Dupl	3	1 SGA, 1 GD, 1 norm	*RP11-12P19.1*
CNVR_25	5	25687036	25723615	5p14.1	36579	Dupl	4	2 GD, 1 SGA, 1 norm	*RNU6-374P*
CNVR_26	5	34107121	34190148	5p13.2	83027	Dupl	8	4 PE, 2 SGA, 1 LGA, 1 norm	*RP11-1250I15.3,RP11-1023L17.1,RP11-1084J3.4*
CNVR_27	5	39113637	39124016	5p13.1	10379	Dupl	3	2 norm, 1 SGA	*FYB*
CNVR_28	5	44513809	44517392	5p12	3583	Dupl	4	2 LGA, 1 GD, 1 norm	-
CNVR_29	5	125946011	126095011	5q23.2	149000	Del	4	2 LGA, 2 norm	*RP11-395C3.1,RP11-772E11.1,HSPE1P10,RP11-434D11.4,RNU6-752P,PHAX,C5ORF48*
CNVR_30	5	140165568	140246287	5q31.3	80719	Dupl	6	3 norm, 2 SGA, 1 PE	*PCDHA14,PCDHA1,PCDHA2,PCDHA3,PCDHA4,PCDHA5,PCDHA6,PCDHA7,PCDHA8,PCDHA9,PCDHA10,AC005609.1*
CNVR_31	5	142770134	142780485	5q31.3	10351	Dupl	4	2 PE, 1 LGA, 1 norm	*NR3C1*
CNVR_32	6	31919577	32017539	6p21.33	97962	Dupl	3	1 LGA, 1 PE, 1 norm	*MIR1236,C4A-AS1,CYP21A1P,TNXA,STK19P,C4B-AS1,CFB,CFB,NELFE,SKIV2L,DXO,STK19,C4A,AL645922.1,C4B,CYP21A2,TNXB*
CNVR_33	6	77439969	77448731	6q14.1	8762	Del	5	2 PE, 2 GD, 1 SGA	-
CNVR_34	6	78979398	79029367	6q14.1	49969	Del/Dupl	1/4	4 norm, 1 LGA	-
CNVR_35	6	93965265	94117546	6q16.1	152281	Dupl	12	4 GD, 4 norm, 2 SGA, 1 LGA, 1 PE	*EPHA7*
CNVR_36	6	101927337	101953555	6q16.3	26218	Dupl	3	2 norm, 1 SGA	*GRIK2*
CNVR_37	6	147635547	147709180	6q24.3	73633	Dupl	3	1 SGA, 1 GD, 1 norm	*STXBP5,RP11-361F15.2*
CNVR_38	6	160812187	160854138	6q25.3	41951	Dupl	6	2 PE, 2 norm, 1 LGA, 1 GD	*SLC22A3,AL591069.1*
CNVR_39	7	11678246	11753281	7p21.3	75035	Dupl	4	2 GD, 1 SGA, 1 norm	*THSD7A*
CNVR_40	7	13439084	13445967	7p21.3	6883	Dupl	7	3 norm, 2 GD, 1 LGA, 1 PE	*AC011288.2*
CNVR_41	7	48310166	48318810	7p12.3	8644	Dupl	7	2 SGA, 2 GD, 1 LGA, 1 PE, 1 norm	*ABCA13*
CNVR_42	7	62154874	62159926	7q11.21	5052	Del	5	2 LGA, 2 PE, 1 norm	-
CNVR_43	7	88568549	88580316	7q21.13	11767	Dupl	3	1 LGA, 1 PE, 1 norm	*ZNF804B*
CNVR_44	7	105036690	105073788	7q22.3	37098	Del	3	2 norm, 1 PE	*SRPK2*

CNVR ID	Chr	Start pos	End pos	Chr band	Length	Type	No of carriers	Groups	Genes
CNVR_45	7	109441794	109451230	7q31.1	9436	Del	4	2 PE, 2 norm	-
CNVR_46	8	2808265	2827657	8p23.2	19392	Dupl	11	5 norm, 2 SGA, 2 PE, 2 GD	*CSMD1*
CNVR_47	8	4272581	4278256	8p23.2	5675	Dupl	3	2 LGA, 1 norm	*CSMD1*
CNVR_48	8	51031221	51033517	8q11.21	2296	Del	3	2 LGA, 1 GD	*SNTG1*
CNVR_49	8	67623174	67677587	8q13.1	54413	Del	4	2 LGA, 2 norm	*C8ORF44-SGK3,SGK3*
CNVR_50	8	84077412	84097208	8q21.13	19796	Dupl	3	1 PE, 1 GD, 1 norm	-
CNVR_51	8	113960534	114194727	8q23.3	234193	Dupl	5	2 LGA, 1 PE, 1 GD, 1 norm	*CSMD3*
CNVR_52	8	144976654	145018354	8q24.3	41700	Dupl	3	1 SGA, 1 LGA, 1 norm	*PLEC*
CNVR_53	9	14590805	14677538	8p22.3	86733	Dupl	4	3 norm, 1 GD	*ZDHHC21,RP11-408A13.1*
CNVR_54	9	19142014	19206930	9p22.1	64916	Del	8	3 PE, 3 GD, 1 LGA, 1 norm	*PLIN2,RP11-146N23.1*
CNVR_55	9	132655864	132830460	9q34.11	174596	Del	3	2 LGA, 1 norm	*RP11-409K20.6,RP11-138E2.1,FNBP1,GPR107*
CNVR_56	9	139240630	139303300	9q34.3	62670	Dupl	3	2 PE, 1 LGA	*GPSM1,DNLZ,CARD9,SNAPC4,SDCCAG3*
CNVR_57	10	20850624	20857365	10p12.31	6741	Del	6	2 SGA, 2 PE, 1 GD, 1 norm	-
CNVR_58	10	63166773	63183583	10q21.2	16810	Dupl	10	4 norm, 4 GD, 1 SGA, 1 PE	*TMEM26*
CNVR_59	10	66885915	66904588	10q21.3	18673	Dupl	4	2 GD, 1 SGA, 1 norm	-
CNVR_60	10	68845232	68888491	10q21.3	43259	Dupl	5	2 GD, 1 SGA, 1 PE, 1 norm	*CTNNA3,LRRTM3*
CNVR_61	10	70174706	70319626	10q21.3	144920	Del	3	2 norm, 1 LGA	*RP11-9E13.4,RNA5SP319,RP11-524O24.2,Y_RNA,TMEM14D,DNA2,SLC25A16*
CNVR_62	10	98513814	98604466	10q24.1	90652	Del	6	3 LGA, 2 PE, 1 norm	*LCOR,MIR607*
CNVR_63	11	9226514	9516186	11p15.4	289672	Del	3	3 PE	*DENND5A,TMEM41B,IPO7,AC132192.1,ZNF143,RP11-5L12.1,RP11-682B13.2,SNORA23,CTD-2371O3.2*
CNVR_64	11	48760364	48942781	11p11.2-p11.12	182417	Del	21	7 norm, 6 PE, 4 LGA, 2 SGA, 2 GD	*RP11-56P9.5,RP11-56P9.10,RP11-56P9.6,RP11-56P9.4*
CNVR_65	11	55365761	55427700	11q11	61939	Del	4	2 norm, 1 PE, 1 GD	*OR4C11,OR4P4,OR4S2*
CNVR_66	11	88696662	88744425	11q14.3	47763	Dupl	7	2 SGA, 2 PE, 2 GD, 1 LGA	*GRM5*
CNVR_67	12	21484512	21497891	12p12.1	13379	Dupl	3	2 norm, 1 GD	*SLCO1A2*

CNVR ID	Chr	Start pos	End pos	Chr band	Length	Type	No of carriers	Groups	Genes
CNVR_68	12	22618605	22647856	12p12.1	29251	Dupl	11	4 norm, 3 SGA, 1 LGA, 2 GD, 1 PE	*C2CD5*
CNVR_69	12	59935926	59942122	12q14.1	6196	Del	4	2 PE, 1 LGA, 1 norm	-
CNVR_70	12	81009228	81091063	12q21.31	81835	Dupl	7	3 GD, 2 LGA, 2 PE	*PTPRQ*
CNVR_71	12	91495837	91519855	12q21.33	24018	Dupl	5	2 PE, 1 LGA, 1 GD, 1 norm	*LUM*
CNVR_72	13	70723292	70763597	13q21.33	40305	Dupl	4	2 norm, 1 SGA, 1 GD	-
CNVR_73	14	47320923	47408344	14q21.3	87421	Dupl	7	4 norm, 1 LGA, 1 SGA, 1 PE	*MDGA2*
CNVR_74	14	84159255	84223764	14q31.2	64509	Dupl	3	2 norm, 1 PE	*RNU6ATAC28P*
CNVR_75	14	106326623	106949307	14q32.33	622684	Dupl	6	3 LGA, 2 norm, 1 PE	*IGHD2-2,KIAA0125,IGHD1-1,IGHV6-1,IGHV1-2,IGHV1-3,IGHV4-4,IGHV2-5,IGHV3-7,IGHV1-8,IGHV3-9,IGHV3-11,IGHV3-13,IGHV3-15,IGHV3-16,IGHV1-18,IGHV3-20,IGHV3-21,IGHV3-23,IGHV1-24,IGHV2-26,IGHV4-28,IGHV3-30,IGHV4-31,IGHV3-33,IGHV4-34,IGHV3-35,IGHV3-38,IGHV4-39,IGHV3-43,IGHJ3P,IGHJ2P,IGHJ1P,AL122127.25,IGHVII-1-1,ADAM6,AB019441.29,IGHVIII-2-1,IGHVIII-5-1,IGHVIII-5-2,IGHV3-6,IGHV2-10,IGHVIII-11-1,IGHV1-12,IGHVIII-13-1,IGHV1-14,IGHVII-15-1,IGHVIII-16-1,IGHV1-17,SLC20A1P2,IGHV3-19,AB019440.50,AB019440.1,IGHVII-20-1,IGHV3-22,IGHVII-22-1,IGHVIII-22-2,HOMER2P2,LINC00226,IGHV3-25,IGHVIII-25-1,IGHVIII-26-1,IGHVII-26-2,IGHV7-27,IGHVII-28-1,IGHV3-29,IGHVII-30-1,IGHV3-30-2,IGHVII-31-1,IGHV3-32,IGHVII-33-1,IGHV3-33-2,IGHV7-34-1,IGHV3-36,IGHV3-37,IGHVIII-38-1,IGHV7-40,AB019438.66,IGHVII-40-1,IGHV3-41,HOMER2P1,IGHV3-42,IGHVII-43-1,IGHVIII-44,LINC00221,IGHVIV-44-1,IGHJ6,IGHJ5,IGHJ4,IGHJ3,IGHJ2,IGHJ1,IGHD7-27,IGHD1-26,IGHD6-25,IGHD5-24,IGHD4-23,IGHD3-22,IGHD2-21,IGHD1-20,IGHD6-19,IGHD5-18,IGHD4-17,IGHD3-16,IGHD2-*

									15,IGHD1-14,IGHD6-13,IGHD5-12,IGHD4-11,IGHD3-10,IGHD3-9,IGHD2-8,IGHD1-7,IGHD6-6,IGHD5-5,IGHD4-4,IGHD3-3
CNVR_76	15	100389182	100415574	15q26.3	26392	Dupl	4	1 SGA, 1 LGA, 1 PE, 1 GD	*CTD-2054N24.2*
CNVR_77	15	102366849	102461162	15q26.3	94313	Dupl	3	1 LGA, 1 PE, 1 norm	*OR4F14P,OR4F13P,OR4F28P,WBP1LP5,AC140725.7*
CNVR_78	16	18982685	18994925	16p12.3	12240	Del	3	1 SGA, 1 LGA, 1 PE	-
CNVR_79	16	58589187	58741850	16q21	152663	Del	3	2 LGA, 1 norm	*SNORA50,CTA-331F8.1,SNOU13,CNOT1,SLC38A7,GOT2*
CNVR_80	17	16058110	16178778	17p11.2	120668	Del	3	2 LGA, 1 norm	*RN7SL442P,NCOR1,PIGL*
CNVR_81	17	27123816	27182944	17q11.2	59128	Del	9	3 PE, 3 norm, 2 LGA, 1 SGA	*FAM222B,ERAL1,RPL31P58,Y_RNA,RP11-20B24.3*
CNVR_82	17	74774824	74820856	17q25.1-p25.2	46032	Del	3	2 LGA, 1 norm	*CTD-2246P4.1,MFSD11*
CNVR_83	18	12096249	12132247	18p11.21	35998	Dupl	4	2 norm, 1 PE, 1 GD	*RNU6-324P,RP11-815J4.4,ANKRD62*
CNVR_84	18	24493424	24498427	18q11.2	5003	Dupl	3	2 norm, 1 LGA	*AQP4-AS1,CHST9*
CNVR_85	18	29554990	29687013	18q12.1	132023	Del	3	1 LGA, 1 PE, 1 norm	*RP11-53I6.2,AC009831.1,RP11-53I6.1,RP11-53I6.3,SNOU13,RNF125,RNF138*
CNVR_86	18	38573994	38630544	18q12.3	56550	Dupl	3	1 SGA, 1 GD, 1 norm	-
CNVR_87	20	7288480	7326701	20p12.3	38221	Dupl	3	1 LGA, 1 PE, 1 norm	-
CNVR_88	20	46000938	46208604	20q13.2	207666	Del	3	2 PE, 1 norm	*RP1-148H17.1,RPL35AP,RNU6-497P,RNU6-563P,AL021394.1,NCOA3*
CNVR_89	20	62038276	62053754	20q13.33	15478	Dupl	4	2 GD, 1 LGA, 1 norm	*KCNQ2*
CNVR_90	22	19228736	19310812	22q11.21	82076	Del	3	2 LGA, 1 norm	*SNORA15,KRT18P62,CLTCL1*
CNVR_91	22	36409990	36525981	22q12.3	115991	Del	3	2 PE, 1 LGA	*RBFOX2*

CNVR, copy number variable region; Chr, chromosome, pos, position; No, number; Dupl, duplication; Del, deletion, norm, normal term; SGA, small-for-gestational age; GD, gestational diabetes; PE, preeclampsia; LGA, large-for-gestational age.

Supplementary Table 8. Genes identified in somatic CNVs in human placental genomes in this study and reported with decreased copy number in mouse placental TGCs[2]

Gene symbol	Name	Chr human	Chr mouse	Function	Reference
7SK	RNA, 7SK small nuclear	*	*	transcription regulation	3
CDH19	Cadherin 19	18	1	calcium-dependent cell adhesion	4
COL11A1	Collagen, type XI, alpha 1	1	3	skeletal morphogenesis	5
CSMD3	CUB and Sushi multiple domains 3	8	15	transmembrane receptor/adhesion	6
DPYD	Dihydropyrimidine dehydrogenase	1	3	pyrimidine metabolism	7
EPHA5	EPH receptor A5	4	5	axon guidance	8
EPHA7	EPH receptor A7	6	4	mediation of cortical neuronal maturation and function	9
GRIK2	Glutamate receptor, ionotropic, kainate 2	6	10	modulation of mossy fiber synaptic strength	10
PTPRD	Protein tyrosine phosphatase (PTP), receptor type, D	9	4	tumor suppression	11
ROBO2	Roundabout, axon guidance receptor, homolog 2 (Drosophila)	3	16	axon guidance	12
SLIT2	Slit homolog 2 (Drosophila)	4	5	axon guidance and neuronal migration	13
SNORA42	Small nucleolar RNA SNORA42/SNORA80 family	*	*	guidance of the sites of modification of uridines to pseudouridines	14
SNORA70	Small nucleolar RNA SNORA70	*	*	biogenesis (modification) of other small nuclear RNAs	15
U3	Small nucleolar RNA U3	*	*	very early pre-rRNA processing	16

* multiple genomic locations; TGC, trophoblast giant cells.

Supplementary Table 9. GO:0005041 'Low-density lipoprotein receptor activity' genes

Gene symbol	Name	Chr	Gene expression in placenta* RNA	Protein	Linked to diabetes	Linked to pregnancy/ placental function	References
CD36	CD36 antigen	5	+	-	+	+	17, 18
CXCL16	chemokine (C-X-C motif) ligand 16	11	+	n.d	+	+	19, 20, 21
LDLR	low density lipoprotein receptor	9	+	n.d	+	+	22, 23, 24
LRP10	low-density lipoprotein receptor-related protein 10	14	++	+++	-	-	
LRP1B	low density lipoprotein-related protein 1B (deleted in tumors)	2	-	n.d	-	-	
LRP6	low density lipoprotein receptor-related protein 6	6	+++	++	+	+	25, 26, 27
LRP8	low density lipoprotein receptor-related protein 8, apolipoprotein E receptor	4	+	n.d	-	+	28
OLR1	oxidized low density lipoprotein (lectin-like) receptor 1	6	+++	++	+	+	29, 30
STAB1	stabilin 1	14	++	++	+	+	31, 32
STAB2	stabilin 2	10	-	++	+	-	31

* Gene expression in placenta at RNA and protein levels according to The Human Protein Atlas; http://www.proteinatlas.org/. +, low expression; ++, medium expression; +++, high expression; n.d, not determined.

References

1. Zhang, B., Kirov, S. & Snoddy, J. WebGestalt: an integrated system for exploring gene sets in various biological contexts. *Nucleic Acids Res.* **33**, W741-748 (2005).
2. Hannibal, R. L. *et al.* Copy number variation is a fundamental aspect of the placental genome. *PLoS Genet.* **10**, e1004290 (2014).
3. Peterlin, B. M., Brogie, J. E. & Price, D. H. 7SK snRNA: a noncoding RNA that plays a major role in regulating eukaryotic transcription. *Wiley Interdiscip. Rev. RNA* **3**, 92-103 (2012).
4. Kools, P., Van Imschoot, G. & van Roy, F. Characterization of three novel human cadherin genes (CDH7, CDH19, and CDH20) clustered on chromosome 18q22-q23 and with high homology to chicken cadherin-7. *Genomics* **68**, 283-295 (2000).
5. Li, Y. et al. A fibrillar collagen gene, Col11a1, is essential for skeletal morphogenesis. *Cell* **80**, 423-430 (1995).
6. Floris, C. et al. Two patients with balanced translocations and autistic disorder: CSMD3 as a candidate gene for autism found in their common 8q23 breakpoint area. *Eur. J. Hum. Genet.* **16**, 696-704 (2008).
7. Chen, C. H. & Cheng, Y. C. The role of cytoplasmic deoxycytidine kinase in the mitochondrial effects of the anti-human immunodeficiency virus compound, 2',3'-dideoxycytidine. *J. Biol. Chem.* **267**, 2856-2859 (1992).
8. Stein, E. et al. A role for the Eph ligand ephrin-A3 in entorhino-hippocampal axon targeting. *J. Neurosci.* **19**, 8885-8893 (1999).
9. Clifford, M. A. et al. EphA7 signaling guides cortical dendritic development and spine maturation. *Proc. Natl. Acad. Sci. U.S.A.* **111**, 4994-4999 (2014).
10. Contractor, A., Swanson, G. & Heinemann, S. F. Kainate receptors are involved in short- and long-term plasticity at mossy fiber synapses in the hippocampus. *Neuron* **29**, 209-216 (2001).
11. Veeriah, S. et al. The tyrosine phosphatase PTPRD is a tumor suppressor that is frequently inactivated and mutated in glioblastoma and other human cancers. *Proc. Natl. Acad. Sci. U.S.A.* **106**, 9435-9440 (2009).
12. Fricke, C., Lee, J. S., Geiger-Rudolph, S., Bonhoeffer, F. & Chien, C. B. astray, a zebrafish roundabout homolog required for retinal axon guidance. *Science* **292**, 507-510 (2001).
13. Wu, J. Y. et al. The neuronal repellent Slit inhibits leukocyte chemotaxis induced by chemotactic factors. *Nature* **410**, 948-952 (2001).
14. Kiss, A. M., Jady, B. E., Bertrand, E. & Kiss, T. Human box H/ACA pseudouridylation guide RNA machinery. *Mol. Cell. Biol.* **24**, 5797-5807 (2004).
15. Ganot, P., Caizergues-Ferrer, M. & Kiss, T. The family of box ACA small nucleolar RNAs is defined by an evolutionarily conserved secondary structure and ubiquitous sequence elements essential for RNA accumulation. *Genes Dev.* **11**, 941-956 (1997).
16. Clery, A., Senty-Segault, V., Leclerc, F., Raue, H. A. & Branlant, C. Analysis of sequence and structural features that identify the B/C motif of U3 small nucleolar RNA as the recognition site for the Snu13p-Rrp9p protein pair. *Mol. Cell. Biol.* **27**, 1191-1206 (2007).
17. Handberg, A., Levin, K., Hojlund, K. & Beck-Nielsen, H. Identification of the oxidized low-density lipoprotein scavenger receptor CD36 in plasma: a novel marker of insulin resistance. *Circulation* **114**, 1169-1176 (2006).
18. Dube, E. et al. Modulation of fatty acid transport and metabolism by maternal obesity in the human full-term placenta. *Biol. Reprod.* **87**, 14, 1-11 (2012).

19 Gutwein, P. et al. CXCL16 and oxLDL are induced in the onset of diabetic nephropathy. *J. Cell Mol. Med.* **13**, 3809-3825 (2009).

20 Zhao, L. et al. Serum CXCL16 as a novel marker of renal injury in type 2 diabetes mellitus. *PloS One* **9**, e87786 (2014).

21 Huang, Y. et al. Chemokine CXCL16, a scavenger receptor, induces proliferation and invasion of first-trimester human trophoblast cells in an autocrine manner. *Hum. Reprod.* **21**, 1083-1091 (2006).

22 Cauchi, S. et al. Analysis of novel risk loci for type 2 diabetes in a general French population: the D.E.S.I.R. study. *J. Mol. Med.* **86**, 341-348 (2008).

23 Ethier-Chiasson, M. et al. Influence of maternal lipid profile on placental protein expression of LDLr and SR-BI. *Biochem. Biophys. Res. Commun.* **359**, 8-14 (2007).

24 Dube, E., Ethier-Chiasson, M. & Lafond, J. Modulation of cholesterol transport by insulin-treated gestational diabetes mellitus in human full-term placenta. *Biol. Reprod.* **88**, 16, 1-10 (2013).

25 Mani, A. et al. LRP6 mutation in a family with early coronary disease and metabolic risk factors. *Science* **315**, 1278-1282 (2007).

26 Singh, R. et al. LRP6 enhances glucose metabolism by promoting TCF7L2-dependent insulin receptor expression and IGF receptor stabilization in humans. *Cell Metab.* **17**, 197-209 (2013).

27 Pollheimer, J. et al. Activation of the canonical wingless/T-cell factor signaling pathway promotes invasive differentiation of human trophoblast. *Am. J. Path.* **168**, 1134-1147 (2006).

28 Wang, L. et al. Polymorphism in maternal LRP8 gene is associated with fetal growth. *Am. J. Hum. Gen.* **78**, 770-777 (2006).

29 Ethier-Chiasson, M. et al. Modulation of placental protein expression of OLR1: implication in pregnancy-related disorders or pathologies. *Reproduction* **136**, 491-502 (2008).

30 English, F. A. et al. Inhibition of lectin-like oxidized low-density lipoprotein-1 receptor protects against plasma-mediated vascular dysfunction associated with pre-eclampsia. *Am. J. Hypertens.* **26**, 279-286 (2013).

31 Tamura, Y. et al. FEEL-1 and FEEL-2 are endocytic receptors for advanced glycation end products. *J. Biol. Chem.* **278**, 12613-12617 (2003).

32 Kzhyshkowska, J. et al. Alternatively activated macrophages regulate extracellular levels of the hormone placental lactogen via receptor-mediated uptake and transcytosis. *J. Immunol.* **180**, 3028-3037 (2008).
